# CellNiche represents cellular microenvironments in atlas-scale spatial omics data with contrastive learning

**DOI:** 10.1038/s41467-026-71759-4

**Published:** 2026-04-22

**Authors:** Zhongming Liang, Bingxu Zhong, Mingqi Jiao, Yong Wang, Shiping Liu

**Affiliations:** 1 Key Laboratory of Systems Health Science of Zhejiang Province, School of Life Science, Hangzhou Institute for Advanced Study, University of Chinese Academy of Sciences, Hangzhou, China; 2 State Key Laboratory of Genome and Multi-omics Technologies, BGI Research, Hangzhou, China; 3 Key Laboratory of Spatial Omics of Zhejiang Province, BGI Research, Hangzhou, China; 4 SJTU-Yale Joint Center for Biostatistics and Data Science, Department of Bioinformatics and Biostatistics, School of Life Sciences and Biotechnology, Shanghai Jiao Tong University, Shanghai, China; 5 State Key Laboratory of Mathematical Sciences, Academy of Mathematics and Systems Science, Chinese Academy of Sciences, Beijing, China; 6 School of Mathematics, University of Chinese Academy of Sciences, Chinese Academy of Sciences, Beijing, China

**Keywords:** Computational models, Machine learning, Computer modelling, Computer science

## Abstract

Deciphering cellular microenvironments at atlas scale remains challenging because molecular identity, spatial context, and platform heterogeneity are tightly coupled. Here we present CellNiche, a scalable contrastive-learning framework that identifies and characterizes cellular microenvironments from spatial omics data using cell-centric spatial-proximity subgraphs. CellNiche combines spatial co-localization and molecular co-expression cues to learn microenvironment-aware embeddings. Across spatial omics datasets from multiple platforms (>10 million cells in total), scaling experiments show improved representations with more training data and competitive clustering and embedding-quality performance with efficient computation. In a multi-sample human non-small-cell lung cancer (NSCLC) cohort, CellNiche identifies conserved and sample-specific tumor and immune microenvironments and captures localized spatial transitions. In four independent mouse brain atlases, CellNiche integrates 293 slices into a unified virtual brain map for cross-atlas annotation transfer and spatial refinement.

## Abstract (web summary)

Deciphering cellular microenvironments at atlas scale remains challenging. Here, authors present a scalable contrastive learning framework using cell-centric subgraphs to map niches across platforms. They apply it to over 10 million cells to reveal spatial patterns in cancer and the brain.

## Introduction

In the single-cell omics era, projecting high-dimensional molecular profiles into low-dimensional embeddings (i.e., representation) has proven to be a highly effective strategy^1–4^. Recent large-scale single-cell modeling frameworks, such as Genformer^5^, scGPT^6^, and SCimilarity^7^, have extended this approach by leveraging larger datasets and powerful architectures, resulting in robust and noise-tolerant representations of cellular identity.

The function of a cell is determined by its molecular state (e.g., gene expression or protein profiles) and its interactions with neighboring cells. The interactions between diverse cell types shape the three-dimensional structure of tissues and underlie both physiological functions and potential pathological transitions. With the advent of spatial omics, the dual-modality nature of the data—molecular features and spatial coordinates—introduces new opportunities and challenges for understanding cellular microenvironments^8,9^. Unlike conventional single-cell analysis, a key conceptual challenge in spatial omics is computationally modeling spatial neighborhoods to reflect cell–cell interactions within tissues and further reflecting biological reality by accounting for both the molecular identity of cells and their spatial context. In addition, scaling these models to datasets containing dozens or even hundreds of spatial slices, while simultaneously identifying both shared and context-specific microenvironments, remains a significant computational bottleneck.

To capture and represent the cell neighborhood information, graph-based approaches, particularly those using graph neural networks (GNNs)^10–12^, have become mainstream. These methods combine hypothesis-driven (leveraging spatial coordinates as explicit structural constraints to define graph topology, where the model does not further modify the graph structure derived from physical coordinates) and data-driven (instead of relying on a predefined spatial graph structure based on physical coordinates, the model infers the interaction patterns through data) modeling. A major line of work follows the representation-learning paradigm from single-cell omics by coupling neighborhood aggregation with molecular feature reconstruction. For example, STAGATE^13^ introduces an encoder–decoder architecture based on graph attention networks (GATs)^12^, jointly modeling neighborhood context and reconstructing gene expression in a self-supervised manner, with the encoder outputs serving as the final embeddings. GraphST^14^ builds on this framework by introducing a contrastive objective between spatial embeddings and their perturbed counterparts to mitigate over-smoothing. In parallel, several methods pursue efficient neighborhood encoding by directly operating on spatial graphs with minimal or no trainable parameters. UTAG^15^ projects molecular features using PCA and performs parameter-free message passing for spatial smoothing, providing computational advantages but with limited capacity for learning complex microenvironmental representations. BANKSY^16^ leverages paired spatial kernels to encode transcriptomic “texture” in local neighborhoods—one based on the weighted mean expression of neighboring cells and the other based on an azimuthal Gabor filter—thereby enhancing spatial features in a lightweight manner. Cytocommunity^17^ emphasizes using phenotypic inputs to reduce noise from raw expression, and it directly uses cell-type annotations as molecular identity features, combining GNNs and graph pooling to map cell-level phenotypes to tissue domains. DECIPHER^18^ adopts a self-contrastive learning scheme in which augmented views of the same cell are brought closer while views of different cells are pushed apart, providing an alternative route to learn cell and spatial-context representations.

Meanwhile, inspired by the success of large-scale models in computer vision and natural language processing, purely data-driven transformer-based architectures have begun to emerge in spatial omics. Unlike GNNs, these models make minimal assumptions about spatial structure, typically incorporating coordinate embeddings only at the input stage and treating molecular features as the primary signal. Most adopt mask-reconstruction objectives. For example, CellPLM^19^ combines expression and spatial embeddings into an initial representation and trains the model by masking and reconstructing a subset of genes in selected cells. scGPT-spatial^20^ initializes its model weights from scGPT^6^ and further pretrains on spatial omics data using masked prediction objectives.

Taken together, GNN-based models are powerful in representing spatial structures but face scalability challenges when integrating heterogeneous inputs from atlas-scale datasets. Transformer-based models are powerful in the pretraining-fine-tune paradigm but have yet to be fully explored in the context of cellular microenvironments and often incur higher computational costs.

To address these limitations, we propose CellNiche, a targeted solution for robust and scalable microenvironment modeling in spatial omics. Our design is guided by two key principles: (1) spatial structure serves as an inductive prior. Hypothesis-driven modeling based on spatial adjacency is inherently suited for capturing local microenvironmental organization, providing an inductive prior that guides the learning process. However, unlike most existing methods that process entire spatial slices—leading to high computational and memory overhead—CellNiche adopts a cell-centric microenvironment mini-batch training strategy. This design not only aligns with the biological reality of local tissue organization but also ensures that the model remains scalable and efficient, regardless of dataset size. (2) CellNiche avoids redundant reconstruction of molecular identity within the spatial module by treating cellular identity features as predefined inputs (e.g., expression-derived features, learned embeddings, or standardized cell-type annotations), rather than reconstruction targets. This enables the model to focus on learning microenvironmental relationships under fixed molecular identities, while allowing batch-effect correction and identity harmonization to be performed flexibly upstream using established approaches. CellNiche then quantifies cell–cell relationships within each local niche using spatial co-localization frequencies and molecular similarity cues and optimizes context-aware embeddings via contrastive learning^21,22^.

The integrated design of the three key components—input module, encoder module, and loss function module—distinguishes CellNiche from existing approaches, making it particularly well-suited for the identification and representation of cellular microenvironments in atlas-scale, heterogeneous spatial omics datasets.

## Results

### CellNiche computationally reconstructs cell niches and represents cell by its molecular identity and neighboring cells

**Fig. 1:**
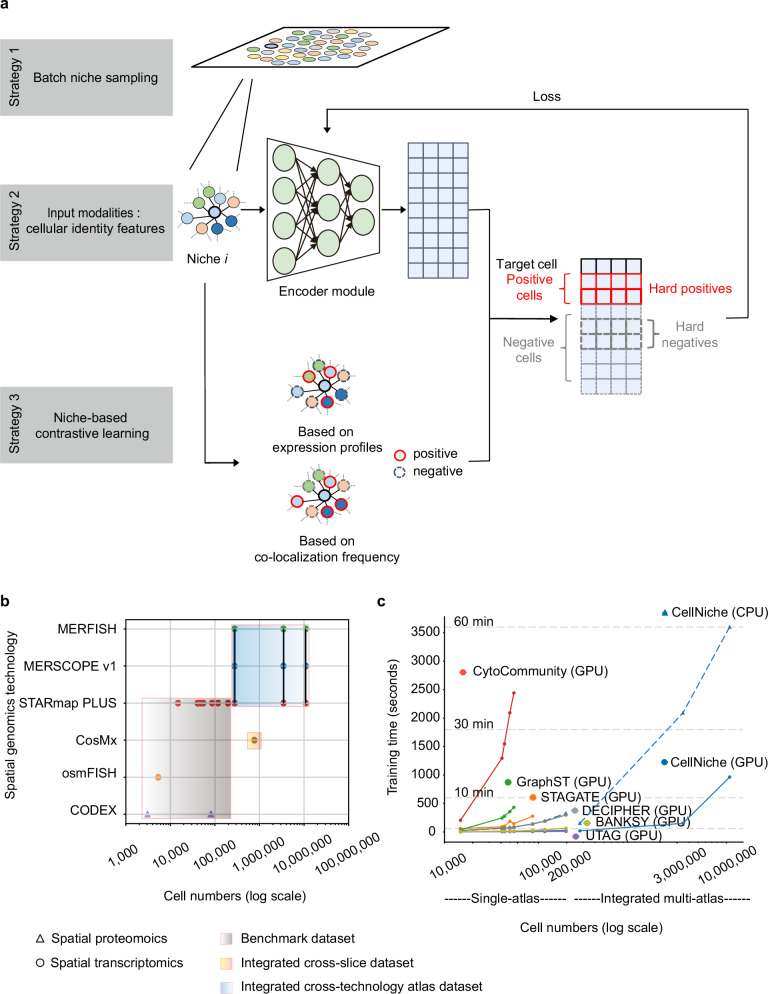
Overview of CellNiche. **a** Overview of CellNiche. CellNiche comprises three core components: the input module, the encoder module, and the loss function module. The input module focuses on cell-centric spatial subgraphs to mimic cellular niches, enabling mini-batch-wise processing that is less sensitive to input data scale. The encoder module applies graph neural networks (GNNs) to cell microenvironment subgraphs, generating low-dimensional embedding vectors for each cell. Within each microenvironment, neighbor cells are defined as either positive or negative samples based on spatial co-localization or co-expression. The loss function module then applies contrastive learning to guide the model in subsequent training steps, using the cell embeddings and positive/negative sample definitions from the current microenvironment. If hard samples are defined, an optional hard-sample-aware contrastive loss can be employed to further strengthen the representations. **b** Summary of the benchmark datasets used in this study, categorized by data scale, omics modality/technology, and application scenarios. **c** (Left) computation time required by different methods to generate microenvironment representations on benchmark datasets (cell counts from ~ 5000–~ 200,000). (Right) computation time for CellNiche to generate microenvironment representations on integrated atlas datasets spanning ~200,000–~ 10,000,000 cells—dashed lines represent computation time on the CPU, while solid lines represent computation time on the GPU. Source data are provided as a Source Data file.

CellNiche is a unified framework designed for atlas-scale spatial omics datasets to identify and characterize cellular microenvironments (Fig. 1a). Its architecture is built upon three key components: input module, encoder module, and loss function module (see *Methods* for details).

In the input module (Fig. 1a, top), CellNiche takes single-cell spatial omics data as input. Cell neighborhoods are constructed using spatial coordinates and a predefined radius^23,24^. Combined with a cell-centric microenvironment mini-batch sampling strategy (Fig. 1a, strategy 1), CellNiche mimics cell niche reconstruction and facilitates mini-batch processing of spatial omics data, enabling efficient, size-insensitive training on datasets of varying scales and resolutions.

In the encoder module (Fig. 1a, center), CellNiche leverages the spatial structure modeling capabilities of GNNs. It takes the sampled cellular subgraphs as input and initializes node features with a fixed cellular identity representation—by default, one-hot cell-type labels—to support direct joint modeling across samples and platforms. More generally, CellNiche can take alternative predefined identity features (e.g., expression-derived features, learned embeddings, or standardized cell-type annotations), allowing batch effects to be handled flexibly upstream without modifying the spatial model (Fig. 1a, strategy 2). The GNN-based encoder then processes these subgraphs to generate latent space embeddings for all cells within the current microenvironment mini-batch.

In the loss function module (Fig. 1a, bottom), CellNiche employs a microenvironment-context-based contrastive learning strategy (Fig. 1a, strategy 3). This strategy utilizes two distinct methods for defining positive and negative cell-pair samples within microenvironments: One approach is based on molecular co-expression, leveraging co-expression patterns to reflect molecular-level proximity between the target cell and its neighbors. The other approach, inspired by the masked token modeling of transformers^25^ (which captures token co-occurrence in language), focuses on spatial co-localization. Given the cellular context, CellNiche abstracts spatial co-localization more efficiently by performing multiple unbiased or biased random walks within each microenvironment. The frequency of these walks serves as a quantitative measure of spatial proximity between the target cell and its neighbors. CellNiche flexibly integrates these approaches to formulate contrastive learning, guiding the model to capture meaningful cell-cell relationships. In addition, an optional hard-sample mining strategy can be adopted.

After training, CellNiche generates a low-dimensional embedding vector for each cell. This embedding vector reflects the cellular microenvironment learned through contrastive targets and is a direct output of the model. Specific cellular microenvironments can be identified by clustering these cell vectors.

### CellNiche shows scalability and computational efficiency on atlas data

We applied CellNiche to a diverse collection of spatial transcriptomic and proteomic datasets from 6 different platforms^26–31^, comprising more than 10 million cells in total (Fig. 1b). CellNiche remained effective on datasets with more than 100,000 cells, and its runtime increased approximately linearly with the number of cells. In contrast, several baseline methods could not be trained on datasets of this scale (Fig. 1c). Based on these considerations, we designated datasets with fewer than 200,000 cells as benchmark datasets (Fig. 1b, gray gradient box) and compared CellNiche with representative state-of-the-art methods using various clustering and embedding quality metrics. For atlas-scale integration, we processed a multi-sample NSCLC cohort^27^ (765,771 cells; Fig. 1b, yellow gradient box) and performed cross-sample training and analysis with CellNiche. We further integrated 10,851,748 cells from 293 mouse brain slices spanning four independent studies across three platforms^31–34^ (Fig. 1b, blue gradient box) and trained CellNiche on the merged atlas, enabling cross-platform mouse brain harmonization and downstream cross-atlas annotation transfer. Notably, training the mouse brain atlas took only ~ 16 min on a single GPU and ~ 1 h on a CPU, demonstrating CellNiche’s scalability and computational efficiency (Fig. 1c, right).

### CellNiche shows accurate and robust performance on spatial transcriptomics data

**Fig. 2:**
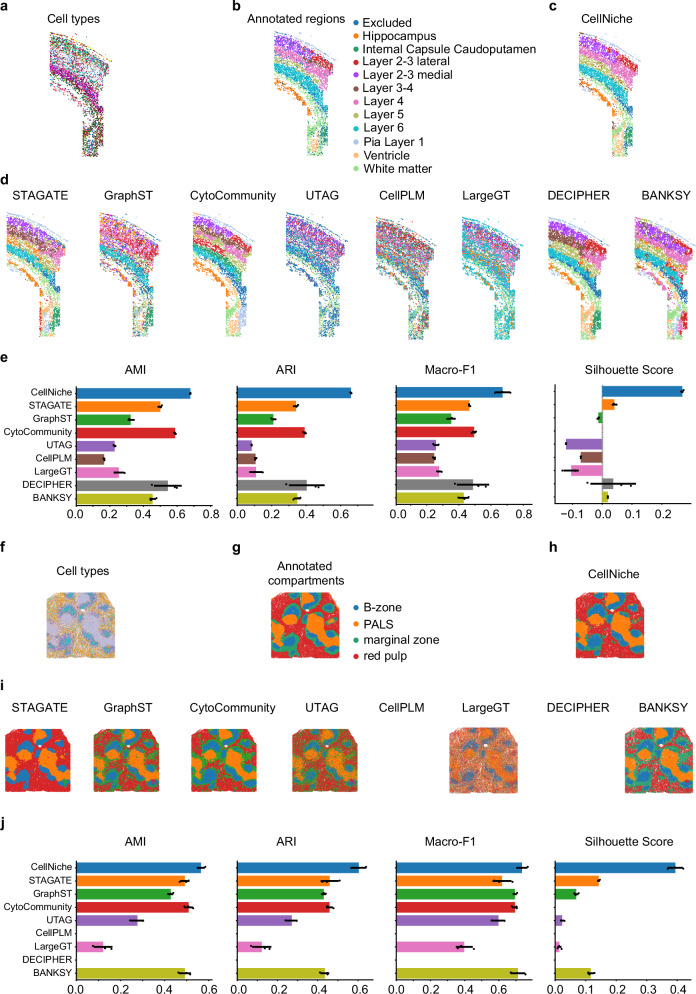
Benchmarking CellNiche across spatial transcriptomics and spatial proteomics. **a**, **b** osmFISH data from the mouse somatosensory cortex, where each cell is colored by its niche label (**a**) and cell type label (**b**). **c** Cellular niches identified by CellNiche on the mouse somatosensory cortex dataset. **d** Cellular niches identified by other methods on the mouse somatosensory cortex dataset. **e** Performance comparison on the mouse somatosensory cortex dataset using ARI, AMI, Macro-F1, and silhouette score metrics. Each method was independently run 3 times. The error bars indicate the mean ± s.d. **f**, **g** Mouse spleen CODEX data from 3 wild-type spleen samples, with each cell colored by its cell type label (**f**) and niche label (**g**). **h** Cellular niches identified by CellNiche on the mouse spleen CODEX dataset. **i** Cellular niches identified by other methods on the mouse spleen CODEX dataset. **j** Performance comparison on the mouse spleen CODEX dataset using AMI, ARI, Macro-F1, and silhouette score metrics. Each method was evaluated on *n* = 3 samples. The error bars indicate the mean ± s.d. Source data are provided as a Source Data file.

We first evaluated CellNiche using a spatial transcriptomics dataset of the mouse somatosensory cortex generated using the osmFISH technology^28^. This dataset captures the canonical layered anatomy of the cortex and comprises 5328 spatially resolved cells profiled for 33 genes, with ground-truth annotations covering 32 cell types and 12 spatial regions (Fig. 2a, b and Supplementary Fig. 1).

We compared CellNiche with a set of representative graph-based and transformer-based baselines spanning distinct modeling paradigms. Graph-based methods included UTAG^15^ and BANKSY^16^, which derive spatial features from local neighborhood aggregation and filtering with lightweight, scalable designs; STAGATE^13^ and GraphST^14^, which learn spatial embeddings using GNN-based encoders; CytoCommunity^17^, which operates on cell-type inputs and directly outputs spatial domains; and DECIPHER^18^, which learns representations via self-contrastive objectives over augmented molecular views. Transformer-based baselines included CellPLM^19^, evaluated in a zero-shot setting using pretrained weights, and LargeGT^35^, trained from scratch to assess performance. This benchmarking protocol was used consistently in all subsequent evaluations.

We quantified agreement with the reference annotations using AMI, ARI and Macro-F1 and additionally reported the silhouette score to assess the compactness and separation of the embedding space. For robust evaluation, each method was run independently three times on each tissue slice. Clustering was performed using K-means with the number of clusters set equal to the number of annotated regions, except for CytoCommunity^17^, which directly outputs spatial domain assignments and does not require additional clustering.

CellNiche achieved the best segmentation of cortical structures, particularly excelling in distinguishing cortical layers 4, 5, and 6, the white matter region, and the hippocampus. In contrast, other methods exhibited varying degrees of boundary confusion and were less effective in capturing clear laminar organization (Fig. 2c, d). Quantitative results further confirmed the superiority of CellNiche, which achieved the highest scores across all 4 evaluation metrics, significantly outperforming all other methods (Fig. 2e). The same conclusion held when using Leiden or Louvain clustering instead of K-means, indicating robustness to the choice of clustering strategy (Supplementary Fig. 2).

In addition to targeted gene-panel datasets, we also evaluated CellNiche on a whole-transcriptome spatial transcriptomics dataset (mouse E16.5 whole embryo Stereo-seq data, comprising 12,215 cells and 15,120 genes)^36^ and obtained competitive results with consistent performance trends (Supplementary Fig. 3).

### CellNiche shows accurate and robust performance on spatial proteomics data

To further evaluate the performance of CellNiche on spatial proteomics data, we applied it to a dataset generated using Co-Detection by Indexing (CODEX), which profiles the mouse spleen microenvironment^30^. This dataset includes three healthy BALB/c spleen samples (BALB/c-1, BALB/c-2, and BALB/c-3), each stained with 29 protein markers. The samples contain 82,251, 81,346, and 80,636 cells, respectively, spanning 27 annotated cell types (Fig. 2g and Supplementary Fig. 4).

The original study manually annotated each image into 4 known anatomical compartments of the spleen: red pulp, marginal zone, B-cell zone, and periarteriolar lymphoid sheath (PALS). We show the spatial visualization of one representative slice (BALB/c-1) (Fig. 2f). We treated these region annotations as ground truth and benchmarked the performance of all methods across the 3 samples.

Consistently across the three samples, CellNiche achieved the best overall quantitative performance across AMI, ARI, Macro-F1 and silhouette score (Fig. 2j). The same conclusion holds when using Leiden or Louvain for niche identification (Supplementary Fig. 5). Notably, CellPLM^19^ and DECIPHER^18^ did not yield informative embeddings on this dataset. For CellPLM^19^, this likely reflects the minimal overlap between its pretraining gene set and the CODEX marker panel (seven shared markers). For DECIPHER^18^, which relies on random-dropout augmentations for self-contrastive learning, the low-dimensional and sparse marker space may lead to information-poor augmented views, compromising representation learning.

### CellNiche performs efficiently on datasets with complex tissue structures and varying scales

Next, we evaluated the scalability and structural sensitivity of all models using a subset of mouse brain spatial transcriptomics datasets generated with STARmap PLUS technology. This subset includes tissue slices with cell counts ranging from 14,829 to 196,416 and regional annotations covering 7 to 69 spatial domains^32^. The benchmarking procedure followed the same protocol as in previous sections. In addition, we recorded the runtime for both model preparation and training phases to assess the computational efficiency and scalability of each model (Supplementary Figs. 6,7). Notably, transformer-based methods^19,20,35^ were excluded from runtime comparisons due to their distinct pretraining–inference paradigm.

**Fig. 3:**
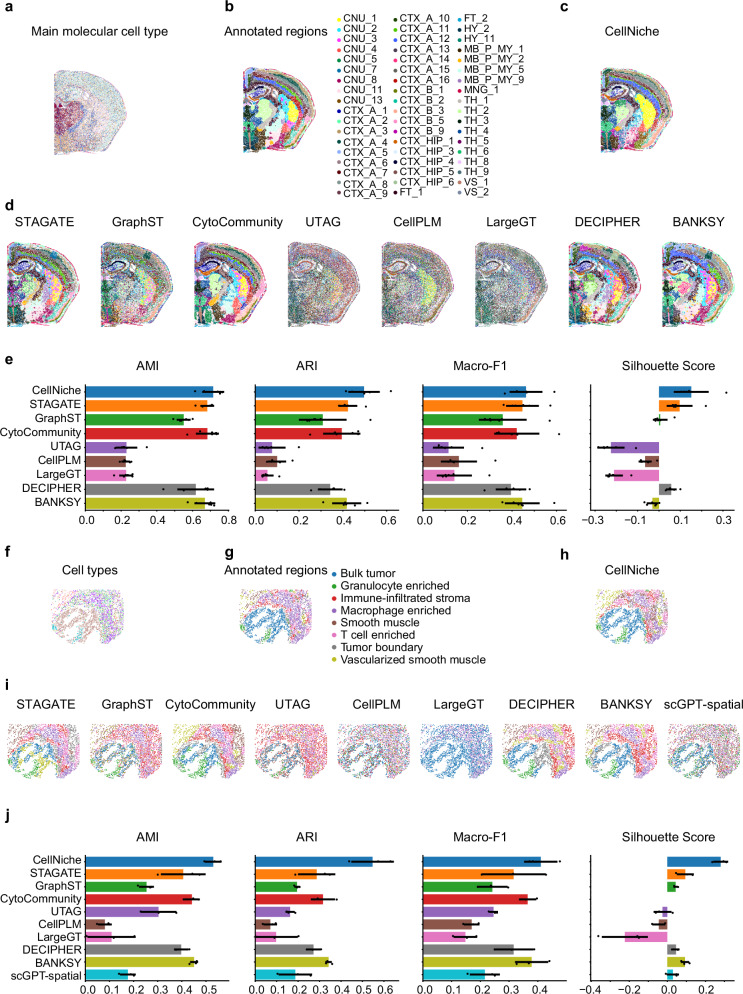
CellNiche performance on complex tissue structures and pathological microenvironments. **a** Coronal slice “well11” of the mouse brain generated using STARmap PLUS technology, where each cell is colored by its niche label (**a**) and cell type label (**b**). **c** Sub-tissue regions identified on “well11” by the CellNiche. **d** Sub-tissue regions identified on “well11” by other methods. **e** Performance comparison on the mouse brain STARmap PLUS data subset using AMI, ARI, Macro-F1, and silhouette score metrics. Each method was evaluated on *n* = 8 samples. The error bars indicate the mean ± s.d. **f**, **g** Human colorectal cancer CODEX slice “reg007_A”, with each cell colored by its cell type label (**f**) and niche label (**g**). **h** Spatial domains identified on “reg007_A” by the CellNiche. **i** Spatial domains identified on “reg007_A” by other methods. **j** Performance comparison on the human colorectal cancer CODEX dataset using AMI, ARI, Macro-F1, and silhouette score metrics. Each method was independently run 3 times. The error bars indicate the mean ± s.d. Source data are provided as a Source Data file.

We illustrate the results using a representative tissue slice (well 11), which comprises 43,341 cells, 1063 cell types, and 63 annotated subregions (Fig. 3a, b and Supplementary Fig. 8). This dataset represents a particularly challenging benchmark due to the heterogeneous and intricate organization of the mouse brain, as well as the higher-resolution annotation task targeting subregional domains. Compared with other methods, CellNiche yielded spatial domains that better respected gross neuroanatomy, with more continuous cortical laminae and more sharply delimited subcortical nuclei (Fig. 3c). In contrast, other methods produced clusters with substantial regional mixing, failing to delineate the fine spatial organization of brain substructures (Fig. 3d). Quantitatively, CellNiche achieved consistently strong performance across all four evaluation metrics, demonstrating a competitive advantage over other methods on this challenging subregional annotation task (Fig. 3e). This performance trend was robust to the choice of clustering strategy, and similar conclusions were obtained when niches were defined using Leiden or Louvain instead of K-means (Supplementary Fig. 9). Moreover, consistent with the design of CellNiche, we observed that CellNiche maintained competitive and stable advantages over other methods when using molecular identity features from different sources as inputs (raw expression, PCA/scVI-derived embeddings, or cell-type features) (Supplementary Fig. 10)

In terms of computational performance, CellNiche showed lower overhead during both model preparation and training. In contrast, GraphST^14^ and CytoCommunity^17^ incur considerable overhead during the preparation phase, and both methods as well as STAGATE^13^ encounter out-of-memory errors when the number of cells scales beyond 100,000. During training, GraphST^14^ and CytoCommunity^17^ failed beyond ~ 50,000 cells, while STAGATE^13^ failed beyond ~ 80,000 cells. Remarkably, CellNiche also demonstrated superior CPU-side performance, outperforming other models trained on GPUs (except UTAG^15^ and BANKSY^16^) in terms of training efficiency (Supplementary Fig. 6), further highlighting its computational scalability.

### CellNiche performs efficiently on datasets with human tumor pathological microenvironments

To extend benchmarking beyond mouse tissues into a clinically relevant tumor setting, we evaluated CellNiche on a human colorectal cancer (CRC) spatial proteomics dataset generated using CODEX. Specifically, we applied CellNiche to the section “reg007_A” from the diffuse inflammatory infiltration (DII) risk group, which contains 3077 spatially resolved cells profiled with 58 protein markers. The original study^37^ provided annotations for 17 cell types and 8 regions (Fig. 3f, g and Supplementary Fig. 11).

In this setting, we additionally included scGPT-spatial^20^, evaluated in a zero-shot manner using pretrained weights. CellNiche produced spatially coherent domains that aligned closely with the reference regions, achieving the highest overall agreement across AMI, ARI, and Macro-F1, and improved embedding quality as reflected by the silhouette score (Fig. 3h–j). Similar trends were observed under alternative clustering strategies (Supplementary Fig. 12).

### CellNiche integrates tumor cohort data and deciphers intratumor heterogeneity

Intratumoral heterogeneity arises from the coexistence of distinct malignant cell populations and diverse immune-stromal components within the tumor microenvironment. Spatial molecular profiling across multiple tumor samples presents a unique opportunity to elucidate the organizational patterns and interactions between tumor and immune-stromal cells. To this end, we applied CellNiche to a spatial transcriptomics cohort of 8 NSCLC tissue sections derived from 5 patients—including 4 lung adenocarcinoma (LUAD) and one lung squamous cell carcinoma (LUSC)—encompassing 765,771 cells in total. All samples were profiled using the Nanostring CosMx platform^27^, which quantified the expression of 960 genes at single-cell resolution and annotated 22 distinct cell types.

**Fig. 4:**
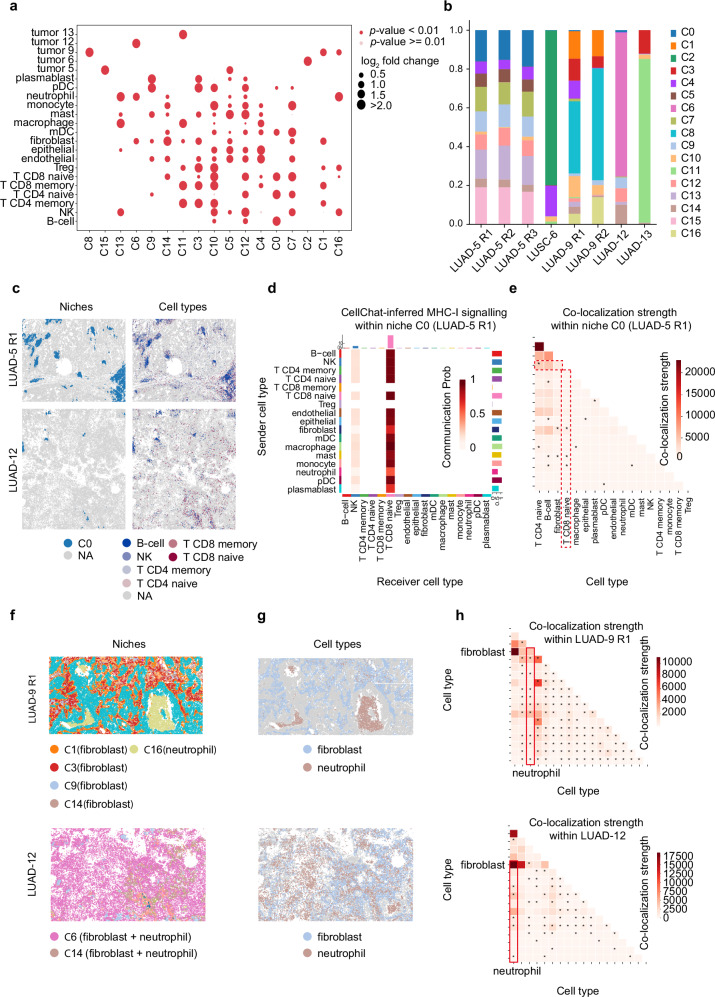
CellNiche deciphers cellular microenvironments in NSCLC. **a** Cell-type enrichment across the 17 CellNiche microenvironments (niches). Dot size denotes log_2_(fold change) and color indicates significance (empirical *p-*values from a permutation test). Significant enrichment is defined as *p*  <  0.01. **b** CellNiche’s spatial niche proportions in each sample. **c** Spatial maps highlighting the lymphoid-enriched niche C0 (left) and the corresponding lymphoid cell types (right) in LUAD-5 R1 (top) and LUAD-12 (bottom). **d** CellChat-inferred MHC-I signaling communication probabilities among cell types within niche C0 in LUAD-5 R1. **e** Proximal cell-cell interactions between different cell types within C0. Color bars indicate the interaction strength between cell type i and cell type j. Asterisks indicate significant pairs based on a one-sided permutation test (*n*  =  1000 permutations) by shuffling cell-type labels; * *p* < 0.05. **f**–**h** Spatial distribution of fibroblast-enriched and neutrophil-enriched niches (**f**), spatial distribution of fibroblast and neutrophil (**g**), and cell-cell interaction strengths within slices (**h**) for LUAD-9 R1 (top) and LUAD-12 (bottom); significance in (**h**) is assessed using the same permutation test as in (**e**).

By jointly training on the full cohort, CellNiche identified 17 distinct cellular microenvironments, guided by clustering stability metrics proposed by Varrone et al.^38^ (see “Methods” for details, Supplementary Fig. 13). Enrichment analysis revealed tumor-associated niches as well as immune- and stroma-dominant niches composed of varying combinations of immune and stromal cell types. Across this cohort, tumor-enriched niches were predominantly patient-specific (Fig. 4a, b and Supplementary Fig. 14), underscoring the substantial inter-patient heterogeneity of the tumor microenvironment. This observation is corroborated by our distance-based analysis of tumor cells, which revealed that inter-patient variation dominates intra-patient variation in the NSCLC cohort (Supplementary Fig. 15)—a pattern recapitulated in the CRC dataset (Supplementary Figs. 16, 17a). Such heterogeneity was further reflected in the CRC dataset by observable niche composition shifts across clinical risk groups, with microenvironmental patterns that appeared differentially associated with high-risk (DII) versus low-risk (CLR) patients (Supplementary Fig. 17b–d).

In contrast, several immune–stromal niches exhibited conserved patterns across samples, suggesting shared immune programs recurring across independent patients. A prominent example was niche C0, which was strongly enriched for lymphoid-lineage cells, including B cells, T CD4 memory, and T CD8 memory^39^, and was predominantly present in LUAD-5 and LUAD-12 (Fig. 4c). This composition and spatial pattern were consistent with tertiary lymphoid structures (TLSs) annotated in the original study (Supplementary Fig. 18a). TLSs are ectopic, organized lymphoid aggregates that arise in chronically inflamed tissues and tumors, often featuring compartmentalized B- and T-cell zones and serving as local sites for antigen presentation and adaptive immune activation. To illustrate how spatial niche structure can contextualize expression-based communication inference, we focused on antigen presentation (MHC-I)^40,41^—a hallmark TLS function and an interaction mode that is inherently contact-dependent^42^. CellChat^43^ inferred a broad set of transcriptionally putative MHC-I interactions involving CD8 naïve T cells and multiple additional cell types within C0 (Fig. 4d). By contrast, spatial co-localization analysis within the same niche showed that the strongest proximity signals for CD8 naïve T cells were concentrated in B cells and CD4 naïve T cells, among the cell types present in C0 (Fig. 4e). Given that effective MHC-I–mediated antigen presentation requires close cell–cell proximity^42^, these results suggest that incorporating spatial niche structure can help refine interaction hypotheses derived from expression-only inference and yield interaction patterns that better reflect local tissue organization.

Furthermore, different spatial mixing patterns of the same cell types may be a driver of the different niche assignments by CellNiche between samples. For instance, neutrophils and fibroblasts had similar proportions in LUAD-9 and LUAD-12 (Supplementary Fig. 18b) but were enriched in different microenvironments: in LUAD-12, both were co-enriched in C6 and C14, whereas in LUAD-9, fibroblasts were scattered across C1, C3, C9, and C14, and neutrophils were concentrated in C16 (Fig. 4f). Spatial visualization (Fig. 4g) and proximity-based interaction networks confirmed stronger spatial co-localization between these two cell types in LUAD-12 (Fig. 4h), supporting the view that niche definitions in CellNiche are driven not only by cell identity but also by spatial interactions. Similar trends were observed under coarser clustering resolutions, where neutrophils and fibroblasts in LUAD-12 co-localized within C7, while those in LUAD-9 mapped to distinct niches (C9 and C10, respectively) (Supplementary Figs. 13, 18c, d).

**Fig. 5:**
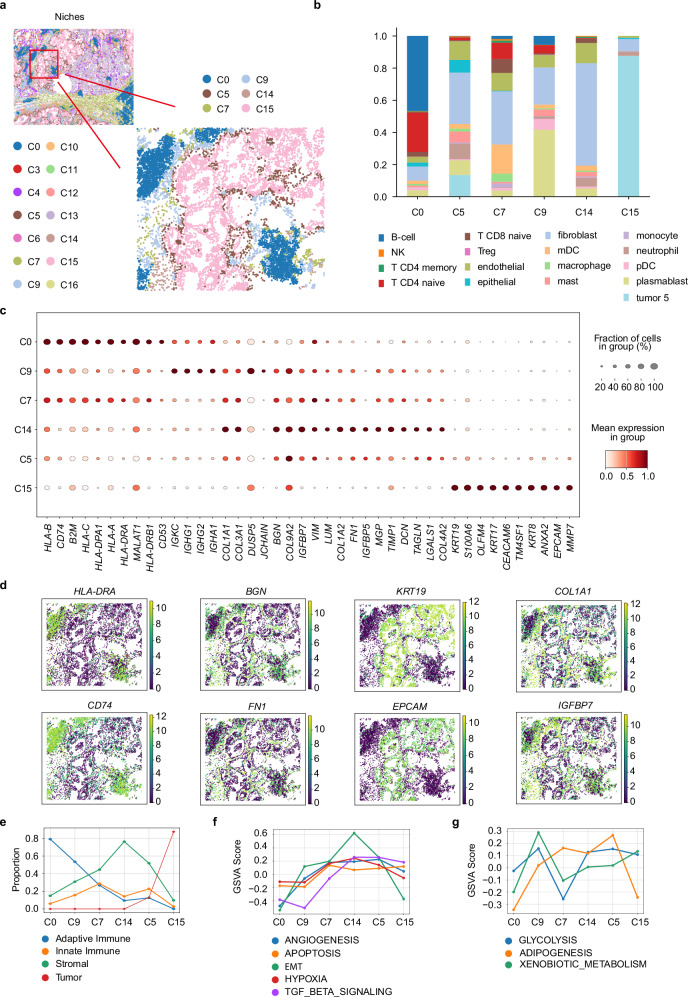
CellNiche identifies and characterizes zonal patterns from lymphoid structures to tumor core regions. **a** Global (upper left) and local (lower right) views of slice LUAD-5 R1. **b** Cell type proportions in each niche (C0, C9, C7, C14, C5, C15).** c** Differentially expressed genes in the niche of the local region in slice LUAD-5 R1. **d** Spatial expression levels of major genes (*HLA-DRA*, *CD74*, *BGN*, *FN1*, *KRT19*, *EPCAM*, *COL1A1*, *IGFBP7*) in the local region of slice LUAD-5 R1. **e** Proportions of major cell types in different cell microenvironments in the local region.** f** GSVA scores for different hallmarks of cancer in different niches in the local region, including ANGIOGENESIS, APOPTOSIS, EMT, HYPOXIA, and TGF_BETA_SIGNALING. **g** GSVA scores for different hallmarks of metabolic pathways in different niches in the local region, including GLYCOLYSIS, ADIPOGENESIS, and XENOBIOTIC_METABOLISM. Source data are provided as a Source Data file.

We further investigated a local region within LUAD-5 R1, where CellNiche identified 6 spatially adjacent microenvironments that correspond to a cellular spatial transition from TLSs, through immune–stroma-enriched zones, to invasive fronts and tumor core regions, as suggested by spatial visualization and cell type composition analysis (Fig. 5a, b). Differential expression gene analysis and spatial mapping of marker genes revealed coordinated molecular and spatial transitions: the outermost region, C0, exhibited high expression of MHC class I and II molecules (such as *HLA-A/B/C*, *HLA-DPA1/DRB1*), along with *CD74* and *MALAT1*, indicating a niche characterized by active antigen presentation^44^. Moving inward, C9 was enriched for immunoglobulin genes associated with plasma cells (including *IGKC*, *IGHG1/2*, *JCHAIN*) as well as early extracellular matrix (ECM) components, suggesting a functional interface between immune and stromal compartments. This transitioned into C7, where ECM remodeling genes (such as *COL3A1*, *IGFBP7*, and *VIM*) were co-expressed with residual immune activity, reflecting an early phase of stromal engagement^45^. Further into the tissue, C14 demonstrated a strong upregulation of genes associated with cancer-associated fibroblasts (CAF), including *FN1*, *DCN*, and *TIMP1*, and represented a matrix-dense niche central to stromal^46^ remodeling. Adjacent to this, C5 exhibited moderate levels of ECM and immune markers, pointing to a transitional invasive front where tumor cells may exploit remodeled matrix pathways for dissemination^47^. Finally, C15, located at the center of the gradient, was dominated by tumor epithelial markers such as *KRT19, KRT17, EPCAM*, and *CEACAM6*, representing the proliferative tumor parenchyma^48,49^ (Fig. 5c, d).

Finally, main cell type proportions and Gene Set Variation Analysis (GSVA)^50^ of hallmark cancer pathways^51^ and key metabolic programs further substantiated the spatial continuum inferred by CellNiche (Figs. 5b, 5e–g). For example, C0 was predominantly composed of adaptive immune cells (~ 79%) and exhibited strong antigen presentation activity. This region showed markedly suppressed pathway activity in angiogenesis (– 0.48), epithelial–mesenchymal transition (EMT, – 0.54), glycolysis (– 0.03), and hypoxia (– 0.11), indicating a highly immune-active and metabolically restrained microenvironment. Progressing inward, C9 presented a mixed composition of ~ 53% adaptive immune cells and ~ 31% stromal cells, and displayed moderate activation of xenobiotic metabolism (0.29) and hypoxia (0.16), reflecting initial immune–stromal interactions^52^. C7, enriched with ~ 29% innate immune cells and ~45% stromal cells, showed increased activity in EMT (0.20), apoptosis (0.14), and adipogenesis (0.16), consistent with early-stage ECM remodeling and inflammatory response^53^. The most stromal-dominated niche, C14 ( ~ 76% stromal cells), exhibited the highest activation in EMT (0.62), TGF-β signaling (0.26), hypoxia (0.24), and glycolysis (0.13), indicative of a dense fibrotic barrier microenvironment^54^. The adjacent region, C5, comprising ~13% tumor, ~ 52% stromal, and ~ 23% innate immune cells, revealed strong upregulation in angiogenesis (0.23), glycolysis (0.16), adipogenesis (0.27), and apoptosis (0.09), forming a metabolically reprogrammed invasive front^55^. At the core, C15 was dominated by tumor epithelial cells (~ 87%) and characterized by moderately activated apoptosis (0.12) and xenobiotic metabolism (0.14) but suppressed EMT (– 0.37) and angiogenesis (0.05), reflecting an immune-cold, proliferation-centric tumor core.

Collectively, these results demonstrate that CellNiche not only effectively captures sample-specific and conserved cellular microenvironments in cross-sample analyses but also provides fine-grained characterization of local microenvironmental transitions. This capability allows CellNiche to reveal continuous spatial gradients, from peripheral immune activation to stromal remodeling and ultimately to the tumor core, reflecting the complex organizational hierarchy within the tumor microenvironment.

### CellNiche integrates cross-platform spatial maps and constructs a unified virtual tissue atlas

CellNiche can integrate heterogeneous spatial omics datasets to construct atlas-scale virtual tissue maps spanning tens of millions of cells. In this study, we integrated spatial transcriptomics data from 4 publicly available mouse brain atlases, generated from different studies: Shi et al.^32^. (Atlas 1, STARmap PLUS), Yao et al.^31^. (Atlas 2, MERSCOPE), Zhang et al.^33^. (Atlas 3, MERFISH), Vizgen^34^ (Atlas 4, MERSCOPE). To harmonize cellular phenotypes across This contrast can be interpreted through the lens of representation learning these datasets, we employed the Allen Institute’s MapMyCells tool (RRID:SCR_024672), which provides a unified taxonomy consisting of 4 hierarchical levels: 35 classes, 339 subclasses, 1202 supertypes, and 5323 clusters. To balance resolution and performance, we chose the “supertype” level as the cell phenotype representation (Supplementary Fig. 19).

**Fig. 6:**
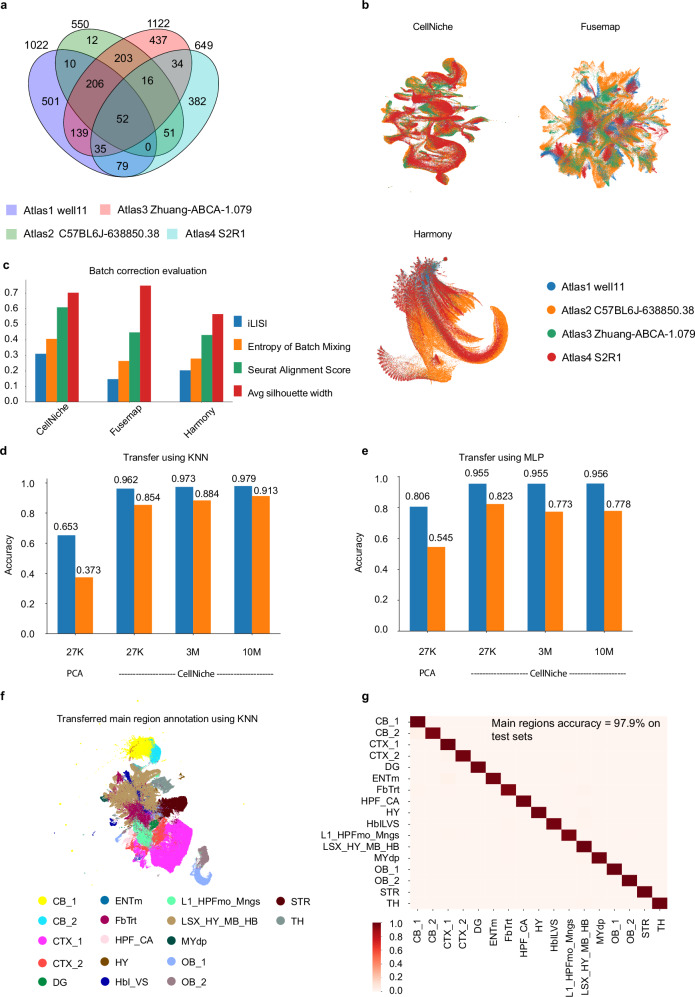
CellNiche can integrate heterogeneous maps across platforms. **a** Intersection size of gene panels in the four representative sections. **b** UMAP projections of cell embeddings generated by CellNiche (top left), FuseMap (top right), and Harmony (bottom left), colored by slice ID. **c** Batch correction evaluation metrics. **d** Annotation transfer accuracy using the KNN method. **e** Annotation transfer accuracy using the MLP method. **f** UMAP projections of cell embeddings generated by CellNiche, colored by main region annotations. **g** Percentage of original main tissue region annotations (rows) transferred from Atlas 1 (columns). Source data are provided as a Source Data file.

To evaluate CellNiche’s integration capability across diverse datasets, we first selected four representative single-cell resolution slices of similar anterior–posterior position, each from a different study and platform. Notably, despite these slices having only 52 overlapping genes (Fig. 6a), CellNiche, leveraging the unified phenotype space strategy, successfully integrated them into a common representation space. We compared CellNiche with two established integration methods: FuseMap^56^ (specifically designed for spatial atlas integration of the mouse brain) and Harmony^57^ (a single-cell data integration method). Integration quality was assessed using four metrics: iLISI, entropy of batch mixing, Seurat alignment score, and average silhouette width. CellNiche outperformed both FuseMap and Harmony^57^ on the first three metrics, and was slightly lower than FuseMap^56^ on average silhouette width (Fig. 6b, c).

To further assess label transfer performance, we extracted cells from Atlas 1^32^ within the integrated post-training representation space and randomly divided them into 80% training and 20% test sets. Two classification approaches were evaluated: the KNeighborsClassifier (KNN) from scikit-learn and a custom-implemented multi-layer perceptron (MLP), both used to predict cell types based on the learned embeddings. We considered three dataset scales: (1) the four benchmark slices comprising 276,321 cells; (2) 20 randomly selected slices from each atlas (or all available slices if fewer than 20), resulting in 3,449,645 cells; and (3) the full dataset including all 293 slices, totaling 10,851,748 cells. As a control, we included a baseline group using the same training data as group (1) but with PCA-derived embeddings instead of CellNiche representations (Fig. 6d, e).

We observed that, across both classification methods, label transfer performance based on CellNiche embeddings consistently outperformed that based on PCA-derived embeddings. Notably, when using PCA as the feature representation, the KNN classifier yielded lower accuracy than the MLP. In contrast, when using CellNiche-learned embeddings, KNN achieved higher annotation accuracy than MLP (Fig. 6d, e). Furthermore, with KNN-based label transfer, annotation accuracy improved steadily with increasing dataset size. While the accuracy for major anatomical regions was relatively saturated, the annotation accuracy for sub-regions improved from 0.854 to 0.913 as the training volume increased (Fig. 6d). Notably, Group 2 included all 20 slices from Atlas 1^32^, and the observed increase in annotation accuracy—from 0.884 to 0.913—not only reflects CellNiche’s enhanced representation capacity gained by supplementing its training with additional atlas datasets, but also demonstrates that our unified input-space strategy allows the model to ignore inter-study variations and benefit from data across independent studies. In contrast, no similar scaling law was observed when using MLP for label transfer (Fig. 6e).

This contrast can be interpreted through the lens of representation learning as follows: KNN preserves CellNiche’s unsupervised representations, while MLP introduces task-specific learning, which changes the embedding of CellNiche as training iterates. As a self-supervised learning framework, CellNiche benefits from increasing training data, enabling the model to acquire richer representations for downstream tasks, while MLP reflects more task-driven training and may overfit to specific label structures.

Building on this unified embedding space and neighbor-based label transfer, we propagated Atlas 1^32^ annotations to all cells across the other three atlases. This enabled harmonized registration of 17 primary molecular brain regions across the four atlases, achieving 97.9% accuracy. These included major anatomical domains such as olfactory bulb (OB), cortex (CTX), nuclei (CNU), thalamus (TH), hypothalamus (HY), midbrain (MB), hindbrain (HB), cerebellum (CB), fiber tracts (FbTrt), and ventricles (VS) (Fig. 6f, g; and Supplementary Fig. 20, 21, 22, 23). Similarly, we successfully transferred 107 subregion annotations with 91.3% accuracy (Supplementary Fig. 24a, b).

**Fig. 7:**
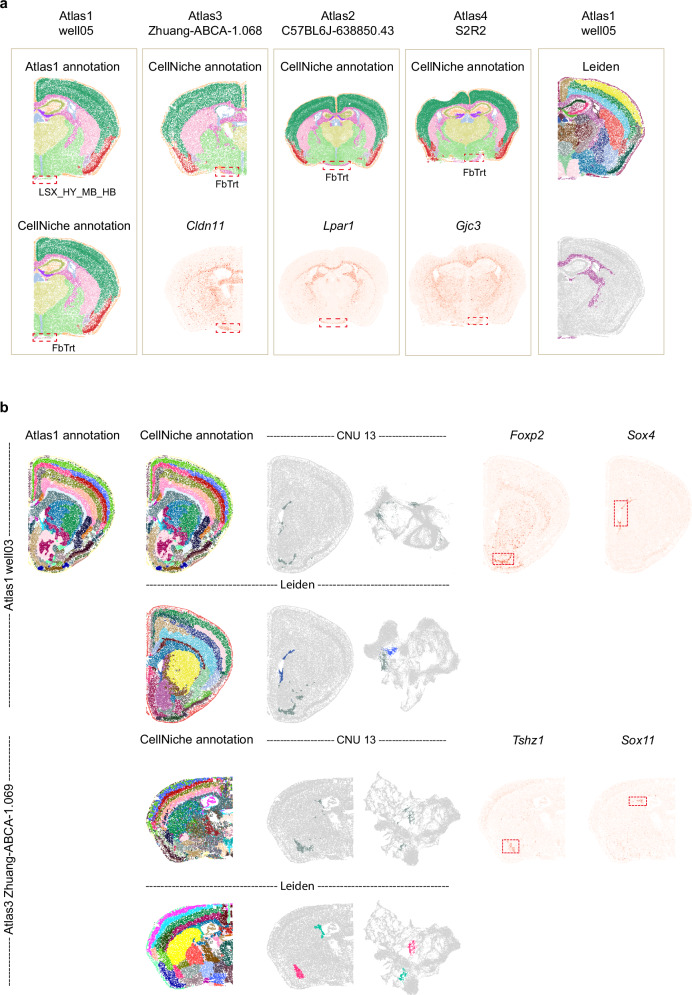
CellNiche enables harmonized integration of tissue regions across atlases. **a** Representative main tissue region refinement in Atlas 1 “well05”, where cells originally annotated as LSX_HY_MB_HB are partially reassigned to FbTrt by CellNiche. The refinement is supported by concordant patterns in spatially similar sections from independent atlases (Atlas 3 Zhuang-ABCA-1.068, Atlas 2 C57BL6J-638850.43, and Atlas 4 S2R2), marker gene expression (*Cldn11*, *Lpar1*, *Gjc3*), and unsupervised Leiden clustering performed on CellNiche embeddings.** b** Example of intra-region substructure within CNU_13, suggesting a two-part subdivision in Atlas 1 well03 supported by *Foxp2* and *Sox4* expression, and a corresponding pattern in Atlas 3 Zhuang-ABCA-1.069 supported by *Tshz1* and *Sox11*. Leiden clustering on CellNiche embeddings further corroborates the spatial separability of the proposed subregions.

We further investigated the discrepancies between the transferred annotations and the original labels, as such differences may reveal the influence of information derived from other atlases. This integration enables the expansion of the limited spatial perspective offered by a single atlas into a more comprehensive whole-organ representation—an outcome that is otherwise difficult to achieve through single-atlas analysis alone. For example, in the well05 slice from Atlas 1^32^, a subset of cells originally annotated as LSX_HY_MB_HB was reassigned to FbTrt after cross-atlas integration. This refinement was supported by consistent spatial patterns observed in anatomically comparable slices from other atlases (e.g., Zhuang-ABCA-1.068 in Atlas 3, C57BL6J-638850.43 in Atlas 2, and S2R2 in Atlas 4), as well as enriched expression of *Cldn11*^58^, *Lpar1*^59^, and *Gjc3*^60^ in the corresponding region (Fig. 7a). Importantly, unsupervised Leiden clustering on the CellNiche embeddings showed that the putative FbTrt cells consistently formed a spatially coherent domain across a wide range of clustering resolutions, indicating that this refinement is robust in the learned representation space (Supplementary Fig. 25a).

Further, we explored whether CellNiche could suggest finer intra-region organization beyond the transferred labels. In the well03 slice from Atlas 1^32^, the CNU_13 region appeared spatially fragmented and showed internal separation in embedding space. marker genes (*Foxp2*^61^, *Sox4*^62^) provided convergent evidence for a two-part subdivision.

To assess whether this subdivision reflects a stable feature of the learned representation—rather than an artefact of a particular clustering resolution or discretization—we performed Leiden clustering on the CellNiche embeddings and evaluated the partition across multiple resolutions. Notably, the two CNU_13 subdomains remained separable even under coarse clustering (i.e., a small number of global clusters) and persisted across decreasing resolutions, indicating that the partition is supported by the continuous embedding geometry rather than a single discretization (Supplementary Fig. 25b).

Consistent evidence was observed in an independent section, Zhuang-ABCA-1.069 (Atlas 3), where the corresponding two subdomains were marked by *Tshz1*^63^ and *Sox11*^64^, respectively (Fig. 7b). Although these two sections are not adjacent, they lie at different positions along the mouse brain rostro–caudal (anterior–posterior) axis, where anatomical structures undergo gradual spatial transitions across coronal sections. The reproducibility of the same bipartition across non-adjacent sections therefore supports the biological plausibility of this proposed subregional refinement.

Together, these results demonstrate that CellNiche not only enables atlas-scale cross-platform spatial integration but also facilitates biologically meaningful refinement and discovery of molecular brain subregions, establishing a scalable and interpretable virtual tissue map of the mouse brain.

## Discussion

In this study, we introduce CellNiche, a scalable framework for identifying and characterizing cellular microenvironments from atlas-scale, heterogeneous spatial omics data. Distinct from previous methods, CellNiche improves scalability to the atlas level by mimicking the cellular niche concept, placing greater emphasis on learning spatial proximity patterns through a cell-centric and mini-batch efficient strategy, and separating identity feature specification from spatial proximity learning. Our results demonstrate that CellNiche consistently outperforms existing methods for cellular microenvironment identification and characterization across multiple datasets and platforms—including both transcriptomic and proteomic modalities—by delivering superior performance in this task while revealing biologically meaningful spatial patterns.

A key strength of CellNiche lies in its strategic decomposition of spatial omics modeling into localized microenvironment-level representation learning. By focusing on cell-centric spatial subgraphs rather than processing entire tissue slices, CellNiche more closely mirrors the biological reality of microenvironmental interactions while significantly reducing spatiotemporal computational costs. Furthermore, CellNiche treats molecular identity as a fixed input and focuses the learnable component on spatial proximity modeling, enabling the characterization of cell–cell relationships within the context of a given molecular identity representation. This design choice prioritizes the modeling of spatial proximity over molecular encoding, yielding more interpretable microenvironmental structures. In contrast to 1D token co-occurrence modeling in language models, CellNiche leverages random walk–derived visit frequencies to construct 2D spatial co-localization “contours”, enabling a topology-aware depiction of cell–cell context. Finally, by relying on phenotype-based inputs rather than raw molecular profiles, CellNiche demonstrates strong robustness to batch effects and technical noise in data integration settings. This adaptability positions it as a scalable framework for harmonizing heterogeneous spatial omics datasets. We anticipate that this strategy will become increasingly valuable in the future, as large-scale initiatives such as the Human Cell Atlas (HCA) and the development of unified phenotype models continue to advance—enabling the integration of cellular data from diverse studies into a shared phenotypic space for comprehensive cross-atlas and cross-platform analyses.

Beyond benchmarking performance, CellNiche demonstrates powerful integrative capabilities in two complex biological settings. First, when applied to NSCLC spatial transcriptomics data, CellNiche reveals both conserved and patient-specific microenvironments shaped by immune and stromal compartments, while also delineating a spatial continuum of tumor progression—from adaptive immune activation, through stromal remodeling, to immune-excluded tumor cores. Second, CellNiche constructs a virtual whole-brain map comprising over 10 million cells by integrating 293 spatial transcriptomic slices across four mouse brain atlases. Through embedding-based label transfer and alignment, it achieves high-accuracy regional annotation across atlases and uncovers finer substructures beyond those captured by original annotations, highlighting its capacity to build coherent spatial representations across platforms.

Together, CellNiche is promising in modeling microenvironments as a cell-contextual learning problem at a scale of multi-million cell atlases and serves as a foundational tool for comparative spatial analysis and virtual tissue map construction in the era of high-throughput spatial omics.

## Methods

### Data preprocessing

The data preprocessing in CellNiche comprises two key components: cellular identity representation and cell’s spatial proximity graph construction.

### Cellular identity representation

CellNiche is designed to flexibly handle two types of cellular features as input: cell gene expression profiles at the molecular level and cell annotation labels at the phenotypic level. For datasets with gene expression information, we directly use the normalized expression matrix to construct the expression-based feature matrix ${F}_{{{{\rm{expr}}}}}\in {{\mathbb{R}}}^{n\times m}$, where $n$ is the number of cells, $m$ is the number of molecules (dimensionality of the expression features). For datasets with phenotype annotations, we apply one-hot encoding to transform the categorical labels of cells into a binary phenotype feature matrix ${F}_{{pheno\; one}-{hot}}\in \{{0,1}\}^{n\times c}$, where $n$ is the number of cells and $c$ denotes the number of unique phenotype categories.

### Spatial proximity graph construction

We construct a spatial proximity graph where nodes represent cells and edges reflect their spatial proximity. This allows us to preserve the spatial topology of the tissue and enables structure-aware sample selection during training. Unlike fixed-k nearest neighbor approaches, CellNiche employs a radius-based strategy, which allows for variable edge densities: denser tissue regions yield more edges, whereas sparser regions form fewer connections. This design better captures the intrinsic spatial heterogeneity of biological tissues. Specifically for single-sample data, we construct the spatial proximity graph using the NearestNeighbors algorithm implemented in scikit-learn. For multi-slice or integrated datasets (e.g., NSCLC cohort or multi-atlas mouse brain), we utilize squidpy to generate slice-specific graphs. For the $i$-th tissue slice, we apply Delaunay triangulation to build an undirected spatial graph ${G}_{i}$, whose adjacency matrix ${A}_{i}$ is stored in either CSR (Compressed Sparse Row) or COO (Coordinate) format. These sparse matrix representations significantly reduce memory usage and improve computational efficiency, especially in large-scale graphs, by storing only the non-zero entries.

### Microenvironment sampling and mini-batch training strategy

CellNiche adopts a microenvironment-aware subgraph sampling strategy combined with mini-batch optimization to enable efficient training on atlas-scale spatial graphs while preserving local structural information. Based on the cell’s spatial proximity graph constructed during preprocessing, CellNiche systematically iterates over each node and applies hierarchical neighborhood sampling to capture its local cellular context. Specifically, given a target cell, we utilize the NeighborSampler module from PyTorch Geometric to recursively sample multi-hop neighbors in a spatial proximity graph to construct a subgraph centered around the target cell. This results in a subgraph-specific adjacency matrix that reflects the local microenvironment of the target cell. These extracted subgraphs are then used as the input units for each mini-batch, ensuring that message passing in the graph neural network occurs within spatially coherent neighborhoods. This design not only improves training scalability but also maintains the biological relevance of spatial proximity during representation learning.

### Cellular microenvironment modeling

To effectively capture local spatial and molecular context, CellNiche introduces a structured strategy for modeling each cell’s microenvironment by leveraging both molecular similarity and spatial co-localization, which is built upon previously sampled target cell-centric subgraphs.

Once the target cell-centric subgraphs are defined, CellNiche characterizes cell–cell interactions within the microenvironment from two optional perspectives:

### Molecular co-expression modeling

From the molecular identity perspective, we compute the pairwise cosine similarity between the target cell and each of its neighbors based on their co-expression. This co-expression similarity is defined as:

1 $${{ES}}_{{ij}}=\frac{{{expr}}_{i}\cdot {{expr}}_{j}}{\parallel {{expr}}_{i}{\parallel }_{2}\parallel {{expr}}_{j}{\parallel }_{2}}$$

where ${{expr}}_{i}$ and ${{expr}}_{j}$ are the molecular feature vectors of cell $i$ and cell $j$, respectively. A co-expression similarity threshold for cell $i$ is then calculated as the average similarity across its niche:

2 $${{similarity}\,{threshold}}_{i}=\frac{{\sum }_{j}\,{{ES}}_{{ij}}}{{deg}_{e}\left(i\right)}$$

Here ${deg}_{e}(i)$ is the degree of cell $i$ in the co-expression subgraph. Cells with similarity above this threshold are labeled as positive samples, while those below are labeled as negative samples.

### Spatial co-localization modeling

Inspired by attention mechanisms in models like transformers, which encode context through co-occurrence patterns, CellNiche models a cell’s spatial co-localization by forming a subgraph and simulating random walks (or biased walks) within each subgraph. A walk is a Markovian node sequence $\left({v}_{0},{v}_{1},\ldots,{v}_{L}\right)$ generated by repeatedly sampling the next node from the current node’s neighbors according to a transition distribution $P({v}_{t+1}| {v}_{t})$. In the default setting, we use a uniform transition over neighbors, i.e., $P(\,j| u)=1/{{{\rm{| }}}}{{{\mathscr{N}}}}(u){{{\rm{| }}}}$ for $j\in {{{\mathscr{N}}}}(u)$, where ${{{\mathscr{N}}}}(u)$ is the neighbor set of node $u$. During each walk, the number of times each neighboring cell is visited is recorded. This visit frequency serves as a proxy for spatial co-localization.

Let ${{freq}}_{{ij}}$ denote the frequency of node $j$ is visited from node $i$ across multiple walks. The mean co-occurrence frequency for node $i$ is defined as its co-localization threshold:

3 $${{threshold}}_{i}=\frac{{\sum }_{j}\,{{freq}}_{{ij}}}{{deg}_{l}\left(i\right)}$$

Here ${deg}_{l}(i)$ is the degree of cell $i$ in the co-localization subgraph. Cells with visit frequencies above this threshold are labeled as positive samples, whereas those below are labeled as negative samples.

The two modeling strategies described above—molecular co-expression similarity and spatial co-localization—can be applied independently or jointly, depending on the experimental setting and data modality. The intersection of both strategies yields a stricter definition of positive interactions; the union of both strategies yields a more inclusive definition. This flexible masking mechanism allows for better control over interaction strength estimation in the cellular microenvironment and adapts to the specific biological characteristics of different datasets.

The final positive and negative interaction labels between the target cell and its niche neighbors are encoded as a binary (0/1) mask matrix, where each element indicates whether a neighboring cell is selected as a positive (1) or negative (0) sample. This mask matrix is used during contrastive training to supervise representation learning by guiding attraction or repulsion between cell embeddings.

### Training module

The node features and adjacency matrix obtained through subgraph sampling serve as the input to the graph neural network. We adopt GraphSAGE^10^, an inductive and scalable graph convolutional architecture, as the encoder for cellular microenvironment representation learning. GraphSAGE^10^ supports efficient mini-batch training and is well-suited for large spatial omics graphs.

Given a graph $G=(V,E)$ with node set $V$ and edge set $E$, let ${x}_{v}$ be the input feature vector for node $v\in V$. These node features are derived from the cell identity representation stage and can correspond to either (i) phenotype-based features obtained via one-hot encoding of cell type, or (ii) expression-based features representing normalized gene expression profiles. The choice depends on the input modality of the given dataset. The node embedding at the input layer is initialized as:

4 $${h}_{v}^{0}={x}_{v}$$

At layer $k$, the representation of node $v$ is updated by aggregating the features of its sampled neighbors ${{{{\mathscr{N}}}}}_{S}(v)$, using the following rule:

5 $${h}_{v}^{k}=\sigma \left({W}^{k}\cdot {{{\rm{CONCAT}}}}\left({h}_{v}^{k-1},{{{{\rm{AGGREGATE}}}}}^{k}\left(\left\{{h}_{u}^{k-1}:u\in {{{{\mathscr{N}}}}}_{S}\left(v\right)\right\}\right)\right)\right)$$

Here, ${{{\rm{CONCAT}}}}(\cdot )$ denotes feature concatenation, ${W}^{k}$ is the trainable weight matrix for layer $k$, $\sigma (\cdot )$ is the nonlinear activation function (ReLU is used in this work), and ${{{{\rm{AGGREGATE}}}}}^{k}(\cdot )$ is the aggregation function (mean aggregation is used in this work).

The final layer *K* node embeddings ${z}_{v}={h}_{v}^{K}$ are then used for downstream contrastive learning.

### Loss function

Given a mini-batch of node embeddings $Z\in {{\mathbb{R}}}^{N\times d}$ obtained from the training module, where $N$ is the batch size and $d$ is the embedding dimension, we compute the similarity matrix ${S}^{0}\in {{\mathbb{R}}}^{N\times N}$ as:

6 $${S}^{0}=\frac{Z\cdot {Z}^{T}}{\tau }$$

Here, the temperature parameter $\tau$ is defined via a learnable parameter $\beta \,{\mathbb{\in }}\,{\mathbb{R}}$ to ensure positivity:

7 $$\tau={{\mathrm{ln}}}\left(1+{e}^{\beta }\right)$$

For numerical stability, each row of the similarity matrix is normalized by subtracting the row-wise maximum value:

8 $${S}_{{ij}}={S}_{{ij}}^{0}-{max }_{k}\,{S}_{{ik}}^{0}$$

To prevent self-comparison, diagonal entries are masked:

9 $${S}_{{ii}}=-\infty$$

Next, the log-probabilities for each pair of samples are computed using a row-wise log-softmax:

10 $$\log {p}_{{ij}}=\log \frac{\exp \left({{{{\rm{S}}}}}_{{ij}}\right)}{{\sum }_{k}\,\exp \left({{{{\rm{S}}}}}_{{ik}}\right)}$$

where ${p}_{{ij}}$ denotes the probability that sample $i$ is similar to sample $j$.

Without hard-sample weighting, the basic contrastive loss is defined as:

11 $${{{{\mathscr{L}}}}}_{{\mbox{unweighted}}}=-\frac{1}{\left|{{{\mathscr{P}}}}\right|}{\sum }_{\left(i,j\right){{{\mathscr{\in }}}}{{{\mathscr{P}}}}}\,\log {p}_{{ij}}$$

where ${{{\mathscr{P}}}}$ denotes the set of positive sample pairs.

To enhance learning from hard samples, CellNiche introduces weighting terms:

For positive pairs $(i,j)\in {{{\mathscr{P}}}}$, define the weight as:

12 $${w}_{{ij}}^{+}=1-{S}_{{ij}}$$

and then normalized:

13 $${w}_{{ij}}^{+}=\frac{{w}_{{ij}}^{+}-\min \left({w}^{+}\right)}{\max \left({w}^{+}\right)-\min \left({w}^{+}\right)+\epsilon }$$

where $\min ({w}^{+})$ and $\max ({w}^{+})$ denote the minimum and maximum positive sample weights, respectively, and $\epsilon$ is a small constant to prevent division by zero.

For negative pairs $(i,j)\in {{{\mathscr{N}}}}$, define the weight as:

14 $${w}_{{ij}}^{-}=\frac{{S}_{{ij}}-\min \left({w}^{-}\right)}{\max \left({w}^{-}\right)-\min \left({w}^{-}\right)+\epsilon }+1.0$$

where $\min ({w}^{-})$ and $\max ({w}^{-})$ denote the minimum and maximum negative sample weights, respectively.

The normalization term across positive and negative samples is:

15 $${Z}_{w}={\sum }_{\left(i,j\right){{{\mathscr{\in }}}}{{{\mathscr{P}}}}}\,\exp \left({S}_{{ij}}\right)+{\sum }_{\left(i,j\right){{{\mathscr{\in }}}}{{{\mathscr{N}}}}}\,\exp \left({S}_{{ij}}\right)\cdot {w}_{{ij}}^{-}$$

where ${{{\mathscr{P}}}}$ and ${{{\mathscr{N}}}}$ represent the sets of positive and negative pairs, respectively.

Then, the adjusted log probability of a positive sample becomes:

16 $$\log {p}_{{ij}}^{{adj}}={S}_{{ij}}-\log \left({Z}_{w}+\epsilon \right),\left(i,j\right){{{\mathscr{\in }}}}{{{\mathscr{P}}}}$$

where $\epsilon$ is a small constant to prevent division by zero.

Finally, the hard-sample-aware contrastive learning loss is formulated as:

17 $${{{{\mathscr{L}}}}}_{{weighted}}=-\frac{1}{\left|{{{\mathscr{P}}}}\right|}{\sum }_{\left(i,j\right){{{\mathscr{\in }}}}{{{\mathscr{P}}}}}\,{w}_{{ij}}^{+}\cdot \log {p}_{{ij}}^{{adj}}$$

This formulation encourages the model to pull together high-confidence and hard-positive pairs, while pushing apart hard-negative pairs with relatively high similarity, thereby learning embeddings that are robust to spatial ambiguity and molecular noise.

### Clustering strategies

During model evaluation, we applied unsupervised clustering to the learned embeddings in order to assess their ability to reveal biologically meaningful cellular microenvironments. Notably, while the Leiden algorithm is commonly used for community detection, its resolution parameter tuning can be time-consuming and subjective, particularly for large-scale datasets. To ensure both efficiency and fair comparison across methods, we consistently applied K-means clustering with a fixed number of clusters to all benchmark datasets, thereby avoiding variability introduced by resolution tuning.

It is important to note that CytoCommunity directly learns the mapping to the spatial domain. Therefore, no additional clustering step is applied for this model.

#### In specific applications

For the NSCLC dataset, we followed the clustering pipeline proposed by Varrone et al., which includes cluster stability assessment and Gaussian mixture–based clustering.

For the integrated mouse brain atlas, we applied Leiden clustering on the learned embeddings to visualize the spatial segregation of specific anatomical or cell-type labels of interest.

Furthermore, to assess the robustness of each model across clustering strategies, we conducted supplementary comparisons on representative slices from five datasets: one slice from the cortical osmFISH dataset, the “BALB/c-1” slice from the mouse spleen CODEX dataset, the coronal “well11” slice from the mouse brain STARmap PLUS dataset, the “reg007_A” slice from the human colorectal cancer CODEX dataset, and the “E16.5_E2S4” slice from the mouse embryo Stereo-seq dataset. We evaluated all methods using three clustering approaches—K-means, Leiden, and Louvain. For K-means, we set the number of clusters to match the number of label categories in each dataset. For Leiden and Louvain, we used the Scanpy^24^ implementations (scanpy.tl.leiden and scanpy.tl.louvain), where the resolution parameters were iteratively optimized to ensure the cluster counts matched the reference label categories for each dataset.

### Comparison of CellNiche with existing tools

To evaluate the performance of CellNiche, we conducted a systematic benchmarking comparison against nine representative spatial modeling methods, including six graph-based methods—STAGATE, GraphST, CytoCommunity, UTAG, DECIPHER and BANKSY—and three transformer-based approaches—CellPLM, LargeGT and scGPT-spatial.

For each dataset, the number of clusters used for evaluation was fixed across all methods and matched the ground-truth annotation to ensure a fair comparison. Input modalities were aligned according to method specifications: CytoCommunity utilized cell type annotations and spatial coordinates, whereas the other methods consumed either protein-level or gene expression matrices along with spatial information.

#### STAGATE (v1.0.0)

We applied STAGATE to all benchmark datasets following the official implementation (https://github.com/QIFEIDKN/STGATE_pyG). Spatial neighbor graphs were constructed using the Cal_Spatial_Net and Stats_Spatial_Net functions. The low-dimensional latent representations were then learned using train_STAGATE, and subsequently clustered using K-means. All parameter settings followed the official documentation.

#### GraphST (v1.1.1)

GraphST was applied to all benchmark datasets using its Python implementation. We adopted the default preprocessing pipeline and hyperparameter settings recommended by the official release.

#### CytoCommunity (v1.1.0)

We evaluated the unsupervised mode of CytoCommunity on all datasets. For the cortex dataset, we used default parameters. For the mouse spleen (CODEX) and mouse brain datasets, we adjusted the number of training epochs (Num_Epoch) to 500 and 400, and set the early stopping threshold (Loss_Cutoff) to -0.4 and -0.6, respectively, in order to reduce training time. All other parameters were kept as the default.

#### UTAG (v0.1.1)

UTAG was applied uniformly across datasets, using the default preprocessing and hyperparameter settings provided in the official implementation.

#### CellPLM

CellPLM serves as a representative pretrained transformer model for zero-shot inference on spatial omics data. We used the publicly available pretrained weights (version 20231027_85M) to initialize the *CellEmbeddingPipeline* and obtained embeddings via the *CellEmbeddingPipeline.predict* method. The embeddings were extracted directly without fine-tuning and evaluated across all benchmark datasets.

#### LargeGT

LargeGT is a transformer-based model that was not originally designed for spatial omics data. We adopt it as a representative of fully trained models specifically for spatial omics data. LargeGT was trained from scratch on each dataset. The number of training epochs is set to 1000 (cortex), 800 (spleen), and 800 (mouse brain) to ensure convergence. All other parameters follow the official default settings.

#### DECIPHER (v0.3.1)

DECIPHER was applied to all benchmark datasets using its Python implementation. We adopted the default preprocessing pipeline and hyperparameter settings recommended by the official release.

#### BANKSY (v1.5.3)

BANKSY was applied to all benchmark datasets using its Python implementation. We adopted the default preprocessing pipeline and hyperparameter settings recommended by the official release.

#### scGPT-spatial

scGPT-spatial serves as a representative pretrained transformer model for zero-shot inference on spatial omics data. We used the publicly available pretrained weights (scGPT-spatial V1 weights) to initialize the model and obtain embeddings via the *scgpt_spatial.tasks.embed_data* method. The embeddings were extracted directly without fine-tuning and evaluated across all benchmark datasets.

### Evaluation

We quantitatively assessed the performance of all compared methods using four commonly used clustering evaluation metrics: AMI, ARI, Macro-F1, and silhouette score. In the cortex dataset, we used cortical structure labels provided by the original publication. In the mouse spleen (CODEX) dataset, compartment labels for three wild-type samples (BALB/c-1, BALB/c-2, and BALB/c-3) were obtained from the CytoCommunity GitHub repository (https://github.com/huBioinfo/CytoCommunity). For the mouse brain (STARmap PLUS) dataset, both coarse and fine-grained region annotations were available. We adopted fine-grained subregional annotations as the gold standard for evaluation.

To assess runtime efficiency, we divided the total running time into two distinct phases: (1) Model preparation stage: including data loading and preprocessing. (2) Model training stage: referring to the actual learning or embedding generation process.

For STAGATE and GraphST, the model preparation and training phases are implemented with separate code. For CytoCommunity, the preparation and training phases corresponded to two distinct script files. For UTAG, the PCA computation (sc.tl.pca) was originally invoked within the clustering module. To ensure accurate measurement of training-only time, we explicitly moved the PCA step outside the clustering block, thereby isolating training time while retaining the original design of the method.

All benchmarking experiments were conducted on a GPU cluster. To further demonstrate the computational efficiency of CellNiche, we additionally evaluated its running time performance on a CPU cluster.

The GPU cluster was equipped with NVIDIA A6000 GPUs (48 GB memory), while the CPU cluster was configured with Intel(R) Xeon(R) Gold 6226 R processors and 365 GB of system memory.

### Integration and processing of cross-atlas mouse brain data

We integrated mouse brain atlas data from four independent studies: atlas 1 (used STARmap PLUS technology, with 17 coronal and 3 sagittal slices from 2 animals), atlas 2 (used MERSCOPE technology, with 59 coronal slices from 1 animal), atlas 3 (used MERFISH technology, with 183 coronal slices and 22 sagittal slices), atlas 4 (used MERSCOPE technology, consisting of 9 coronal brain slices (3 repeats from 3 coronal planes)). Atlas 2 and atlas 3 share consistent cell type annotations (from the Allen Institute’s four-level annotation), while atlas 1 and atlas 4 have their own independent cell type annotations. To unify the phenotype space, we re-annotated the cells in atlas 1 and atlas 4 using the “MapMyCells” tool from the Allen Institute, with the reference taxonomy set to 10x Whole Mouse Brain (CCN20230722), and hierarchical mapping as the annotation algorithm. To ensure annotation quality, we retained only cells with a ‘bootstrapping_probability’ greater than 0.5.

### Determination of the Number of Cellular Microenvironments in the NSCLC Dataset

The number of cellular microenvironments was determined using a clustering stability procedure proposed by Varrone et al. This method evaluates the robustness of clustering results at adjacent *K* values. Briefly, Gaussian mixture models (GMMs) are fitted at *K*-1, *K*, and *K* + 1, respectively, and the similarity between microenvironments is quantified using the Fowlkes-Mallows index (FMI). *K-*values that consistently maintain a high FMI value across multiple runs are determined as stable *K-*values. For the selected *K-*value, the GMM model with the highest marginal likelihood is retained.

### Gene set variation analysis (GSVA)

To quantify pathway-level activity across the cellular microenvironments identified by CellNiche, we performed GSVA on pseudo-bulk expression profiles. For each niche, log-normalized gene expression values were averaged across all cells to obtain a cluster-by-gene matrix. GSVA was performed using the *gseapy* (v.1.1.11) implementation of the original GSVA method, with the MSigDB Hallmark gene set collection (h.all.v2024.1). Enrichment scores were computed using the default Gaussian kernel (kcdf = “Gaussian”). The resulting GSVA matrix (pathway × niche) was used to evaluate biological programs associated with immune activation, stromal remodeling, and metabolic transitions.

### Differential gene expression analysis

Differentially expressed genes for each CellNiche microenvironment were identified using the Wilcoxon rank-sum test implemented in *scanpy.tl.rank_genes_groups*. Each microenvironment was compared against all remaining cells (“rest”), and *p*-values were adjusted using the Benjamini–Hochberg false discovery rate (FDR). Log-normalized expression matrices were used as input. For each microenvironment, the top-ranked genes were selected for visualization and downstream interpretation.

### Batch correction with harmony

Batch correction across samples was performed using Harmony via the *sce.pp.harmony_integrate* function. Log-normalized expression values were first reduced using principal component analysis (*sc.pp.pca*). Harmony integration was then applied with the sample identifier specified as the batch covariate (*sce.pp.harmony_integrate*(adata, key = “sample”)), using default parameters. The corrected PCA embeddings were used for downstream analysis.

### Integration with FuseMap

FuseMap was applied following the official implementation and default settings provided by the authors. No hyperparameters or preprocessing procedures were modified. The method was used as released, ensuring consistency with the original design of FuseMap.

### Reporting summary

Further information on research design is available in the Nature Portfolio Reporting Summary linked to this article.

## Supplementary information

Supplementary Information

Reporting Summary

Transparent Peer Review file

## Source data

Source data

## Data Availability

The osmFISH dataset of mouse somatosensory cortex is available at https://github.com/drieslab/spatial-datasets^28^. The mouse spleen CODEX dataset is available at https://data.mendeley.com/datasets/zjnpwh8m5b/1^30^. The STARMap dataset of mouse brain is available at https://singlecell.broadinstitute.org/single_cell/study/SCP1830^32^. The human CRC CODEX dataset is available at https://data.mendeley.com/datasets/mpjzbtfgfr/1^37^. The NSCLC CosMx dataset is available at https://nanostring.com/products/cosmx-spatial-molecular-imager/nsclc-ffpe-dataset/^27^. The spatial transcriptomics atlases of mouse brain are available at https://singlecell.broadinstitute.org/single_cell/study/SCP1830 (Atlas 1)^32^, https://doi.brainimagelibrary.org/doi/10.35077/g.610 (Atlas 2)^31^, https://doi.brainimagelibrary.org/doi/10.35077/act-bag (Atlas 3)^33^, https://info.vizgen.com/mouse-brain-map (Atlas 4)^34^. The mouse E16.5 whole embryo Stereo-seq data is available at https://db.cngb.org/stomics/mosta/download/^36^. Source data are provided in this paper.

## Code availability

The software package implementing the CellNiche algorithm has been deposited at GitHub https://github.com/Super-LzzZ/CellNiche under the MIT license. The version associated with this study has been archived at Zenodo (10.5281/zenodo.19143524)^65^.
