# Supplementary material for: CellNiche represents cellular microenvironments in atlas-scale spatial omics data with contrastive learning
Source: Nat Commun. 2026 Apr 22;17:5547. doi: 10.1038/s41467-026-71759-4 (PMC13287597; doi:10.1038/s41467-026-71759-4)
Supplement: Supplementary file 1 — Supplementary Information [file 41467_2026_71759_MOESM1_ESM.pdf]

CellNiche represents cellular microenvironments in atlas-scale spatial omics data with contrastive learning

Zhongming Liang[1,2,3]✉, Bingxu Zhong[4], Mingqi Jiao[1], Yong Wang[1,5,6]✉, Shiping Liu[1,2,3] ✉

1. Key Laboratory of Systems Health Science of Zhejiang Province, School of Life Science, Hangzhou Institute for Advanced Study, University of Chinese Academy of Sciences, Hangzhou 310024, China.
2. State Key Laboratory of Genome and Multi-omics Technologies, BGI Research, Hangzhou 310030, China.
3. Key Laboratory of Spatial Omics of Zhejiang Province, BGI Research, Hangzhou 310030, China.
4. SJTU-Yale Joint Center for Biostatistics and Data Science, Department of Bioinformatics and Biostatistics, School of Life Sciences and Biotechnology, Shanghai Jiao Tong University, Shanghai, China.
5. State Key Laboratory of Mathematical Sciences, Academy of Mathematics and Systems Science, Chinese Academy of Sciences, Beijing 100190, China.
6. School of Mathematics, University of Chinese Academy of Sciences, Chinese Academy of Sciences, Beijing 100049, China.

✉ Corresponding authors. liangzhongming21@mails.ucas.ac.cn (Z.M.L.), ywang@amss.ac.cn (Y.W.), liushiping@genomics.cn (S.P.L.)

Supplementary Figures

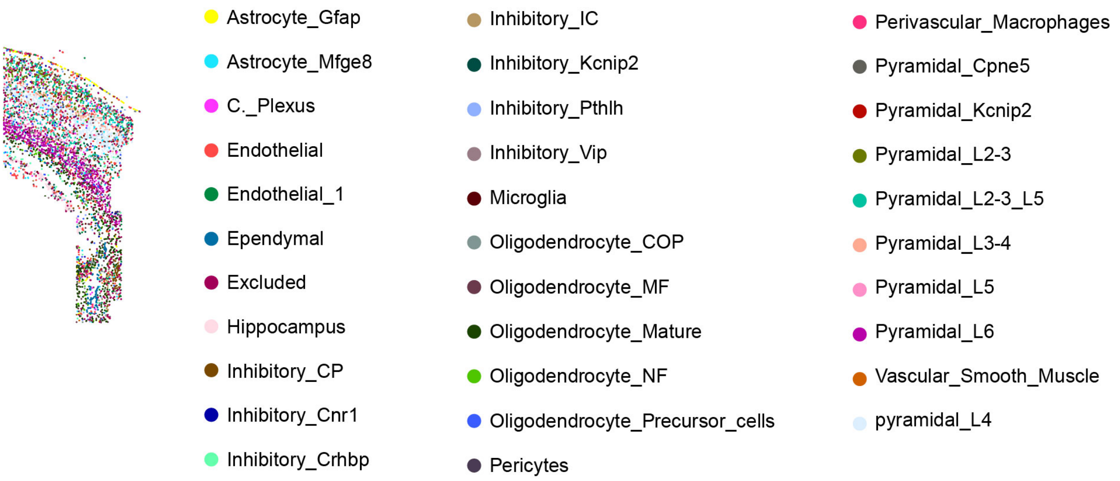

Supplementary Fig. 1: Spatial visualization of cell types in mouse somatosensory cortex slice.

Cells are colored according to cell types.

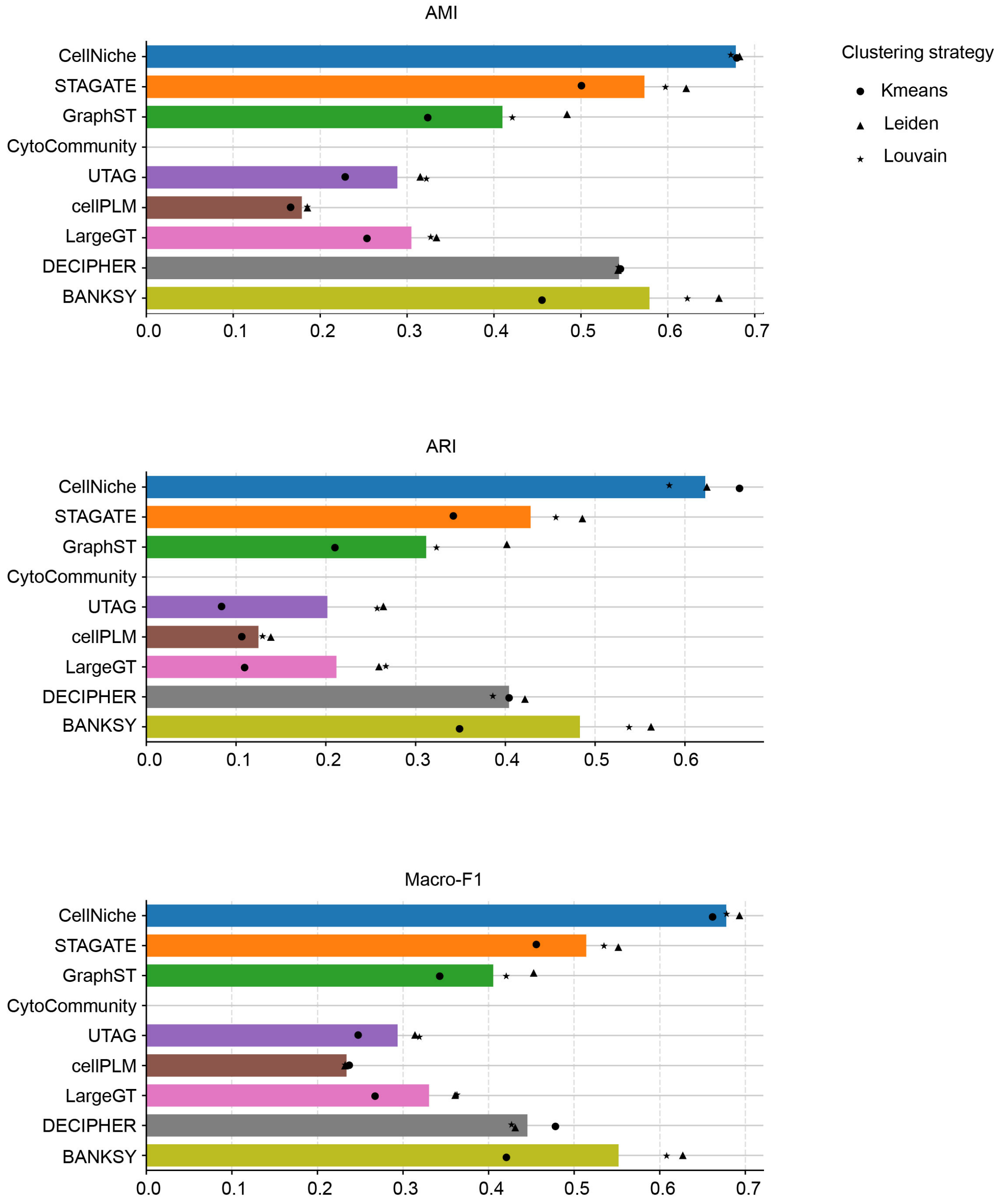

Supplementary Fig. 2: Benchmark performance under different clustering strategies on the mouse somatosensory cortex osmFISH dataset.

Bar plots show the average agreement with the reference region annotations across three clustering strategies (K-means, Leiden, and Louvain) for each method. Marker symbols indicate the scores obtained under each clustering strategy (circle: K-means; triangle: Leiden; star: Louvain). Metrics include AMI, ARI, and Macro-F1. Notably, the absence of a Leiden or Louvain marker for a specific metric indicates that the target number of clusters (matching the reference labels) could not be achieved, even after the resolution parameter was iteratively tuned to five decimal places. Source data are

provided as a Source Data file.

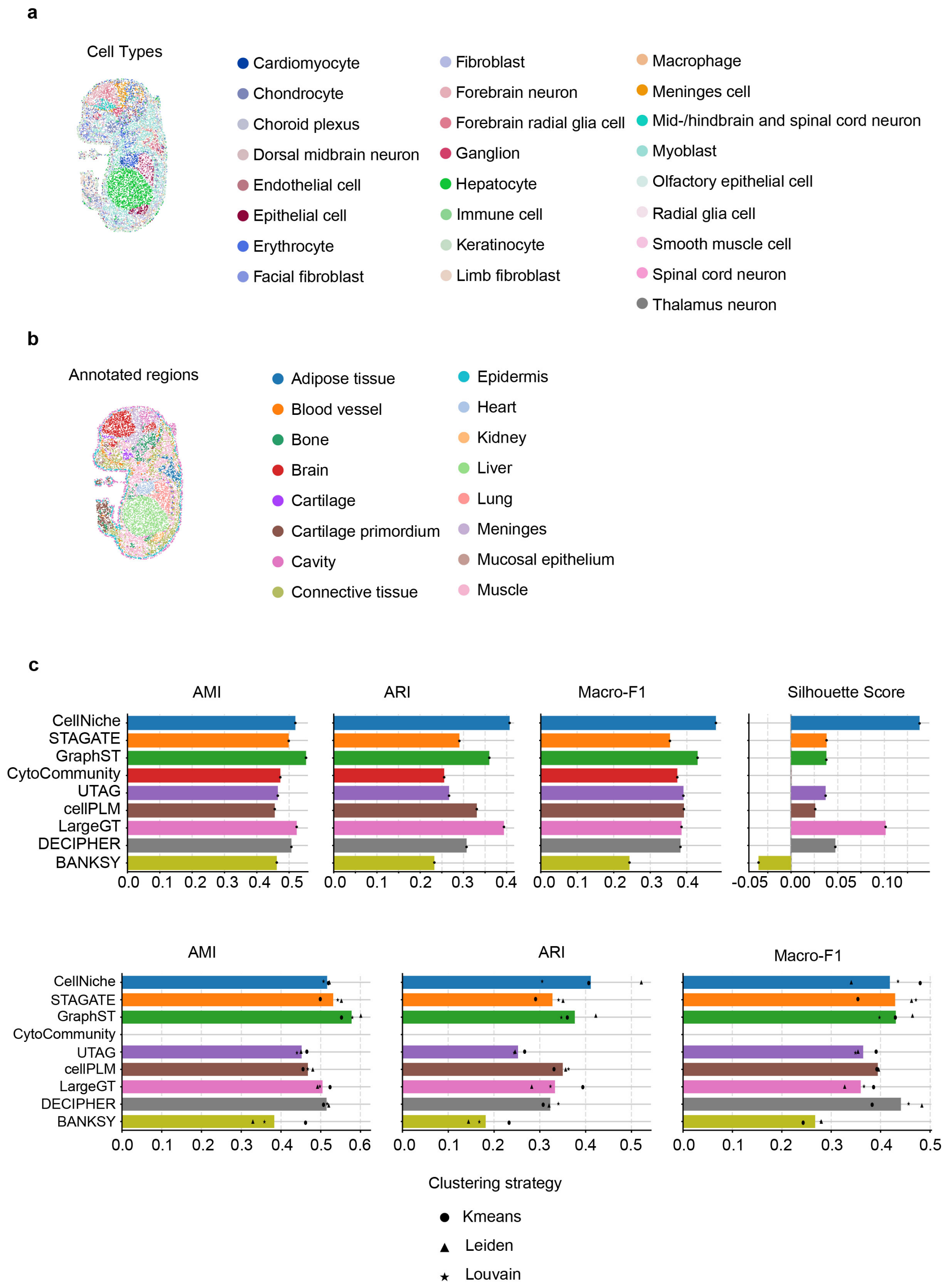

Supplementary Fig. 3: Benchmark performance under different clustering strategies on the Stereo-seq mouse embryo dataset (slice "E16.5_E2S4").

a, Spatial map colored by annotated cell types. **b,** Spatial map colored by annotated tissue regions. **c,** Benchmark comparison across methods using AMI, ARI, Macro-F1, and silhouette score. Top row: scores obtained using K-means clustering, consistent with the main text settings. Bottom row: mean scores under three clustering strategies, and marker symbols indicate the scores obtained under each

clustering strategy (circle: K-means; triangle: Leiden; star: Louvain). Notably, the absence of a Leiden or Louvain marker for a specific metric indicates that the target number of clusters (matching the reference labels) could not be achieved, even after the resolution parameter was iteratively tuned to five decimal places. Source data are provided as a Source Data file.

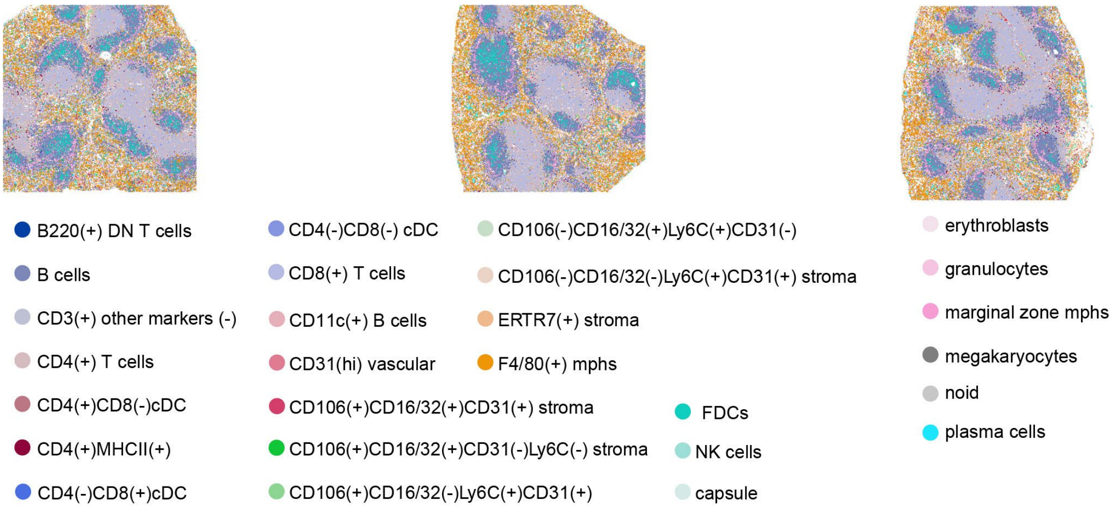

Supplementary Fig. 4: Spatial visualization of cell types in mouse spleen datasets.

Cells are colored according to cell types.

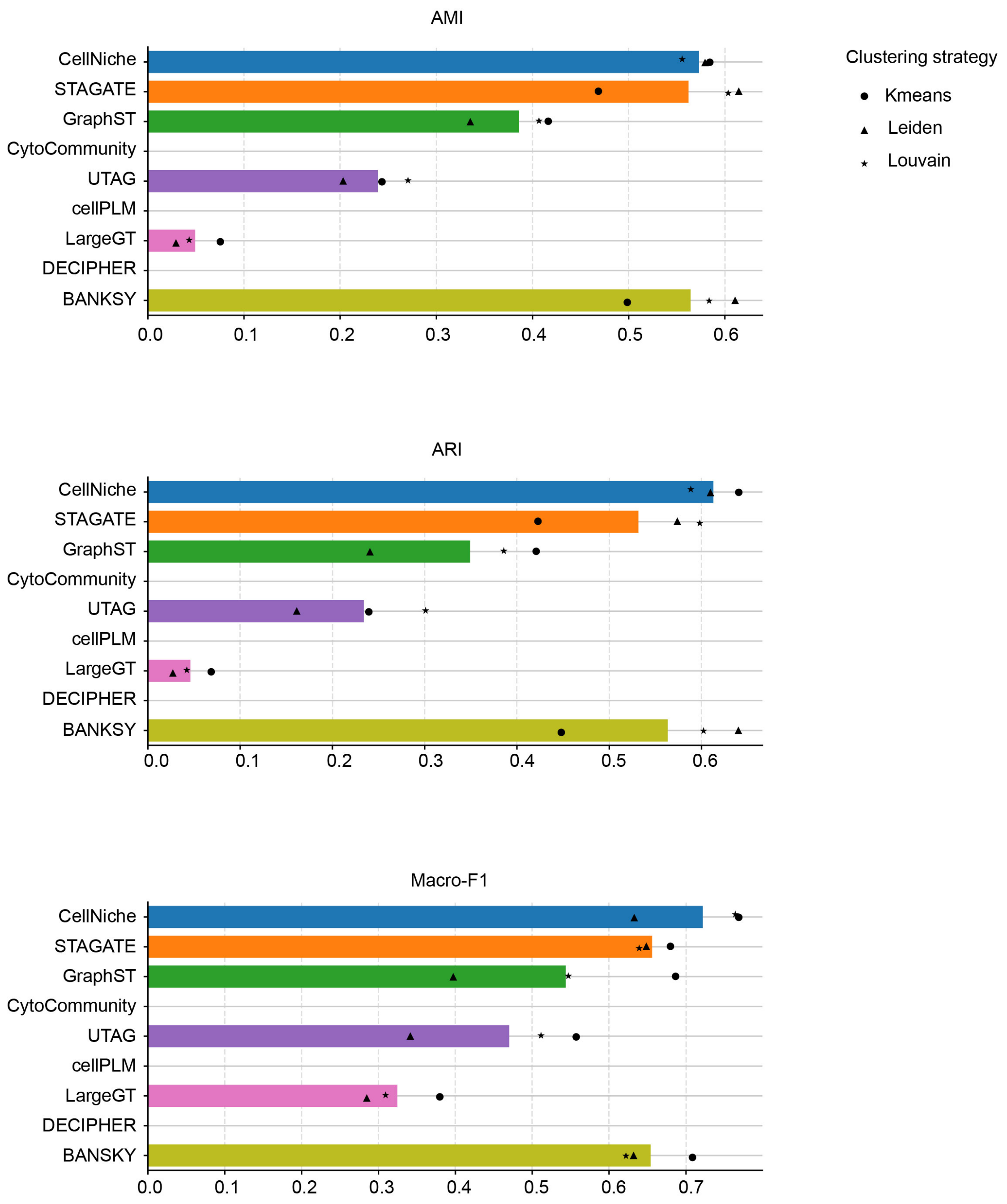

Supplementary Fig. 5: Benchmark performance under different clustering strategies on the mouse spleen CODEX dataset (slice "BALB/c-1").

Bar plots show the average agreement with the reference region annotations across three clustering strategies (K-means, Leiden, and Louvain) for each method. Marker symbols indicate the scores obtained under each clustering strategy (circle: K-means; triangle: Leiden; star: Louvain). Metrics include AMI, ARI, and Macro-F1. Notably, the absence of a Leiden or Louvain marker for a specific metric indicates that the target number of clusters (matching the reference labels) could not be achieved, even after the resolution parameter was iteratively tuned to five decimal places. Source data are provided as a Source Data file.

a

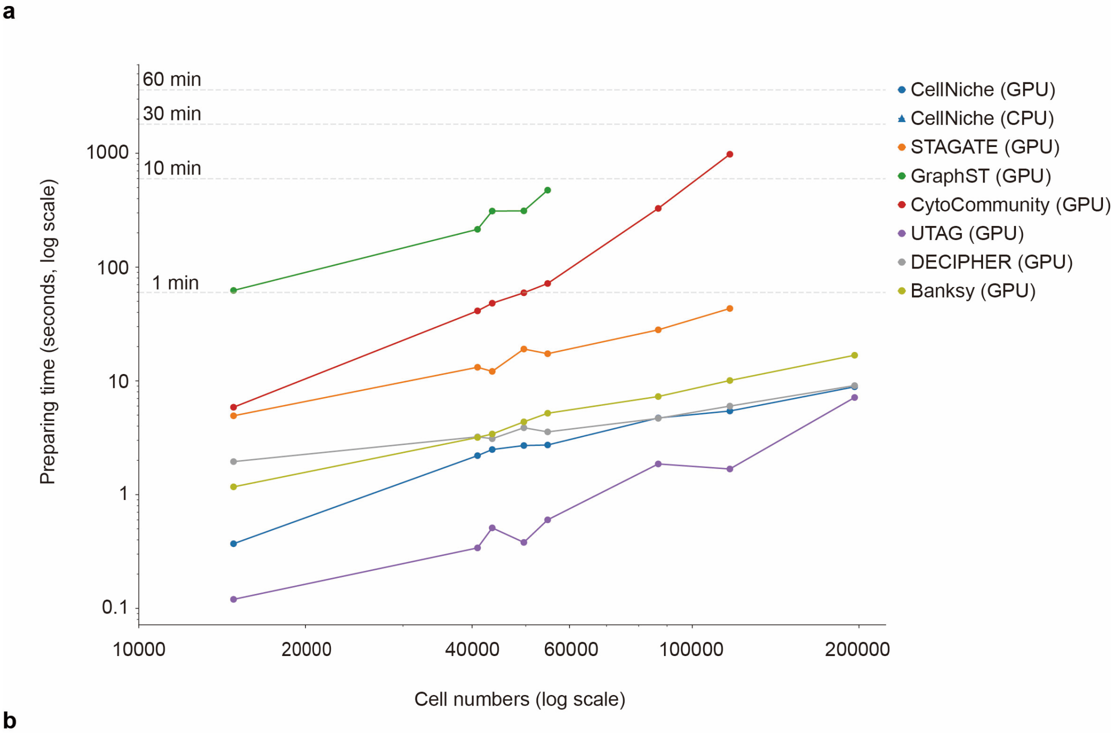

b

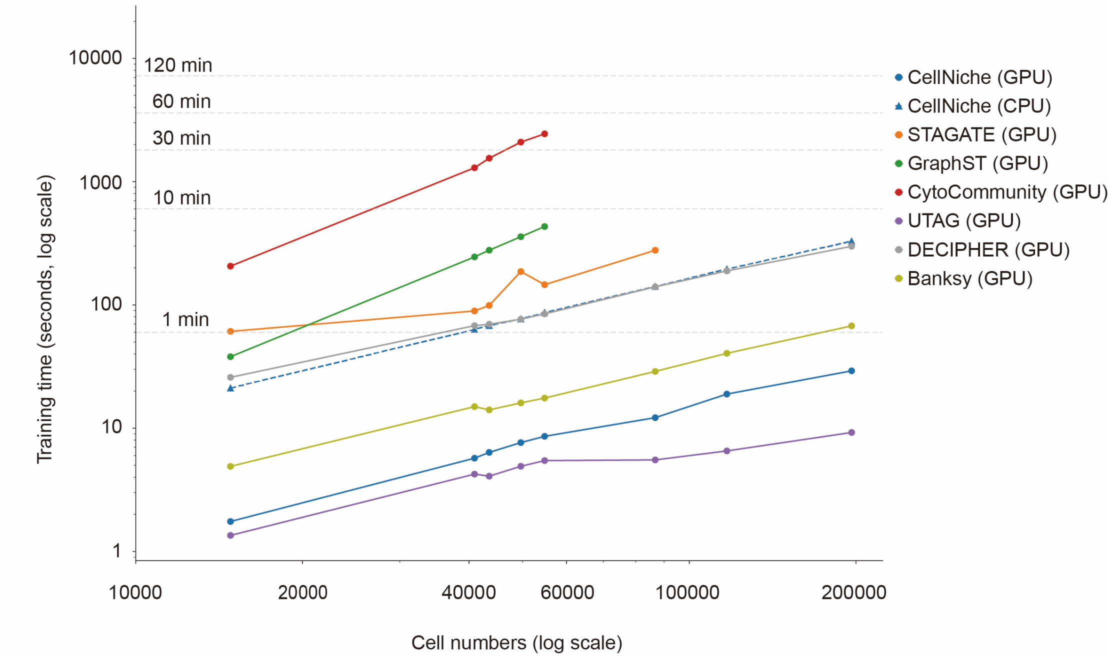

Supplementary Fig. 6: Time cost comparison experiment of each method.

a, b, Time consumption for model preparation (a) and model training (b) of each method on the mouse brain STARmap PLUS data subset. The GPU cluster was equipped with an NVIDIA A6000 GPU (48 GB memory), while the CPU cluster was configured with Intel(R) Xeon(R) Gold 6226R processors and 365 GB of system memory. Source data are provided as a Source Data file.

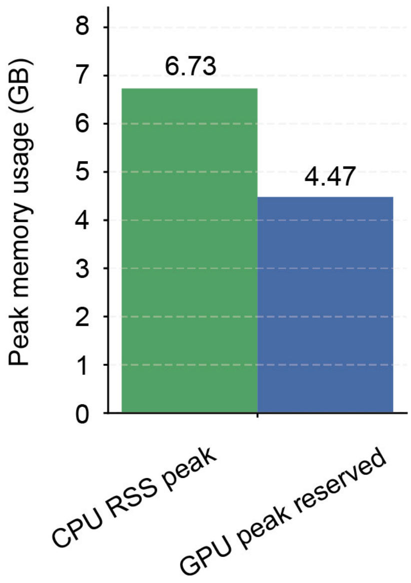

Supplementary Fig. 7: Peak memory usage during CellNiche training on a STARmap PLUS dataset.

Peak CPU resident memory (RSS) and peak GPU reserved memory were recorded during training on the “sagittal3” mouse brain slice (196,416 cells). Measurements were obtained under a single NVIDIA A6000 GPU configuration. Source data are provided as a Source Data file.

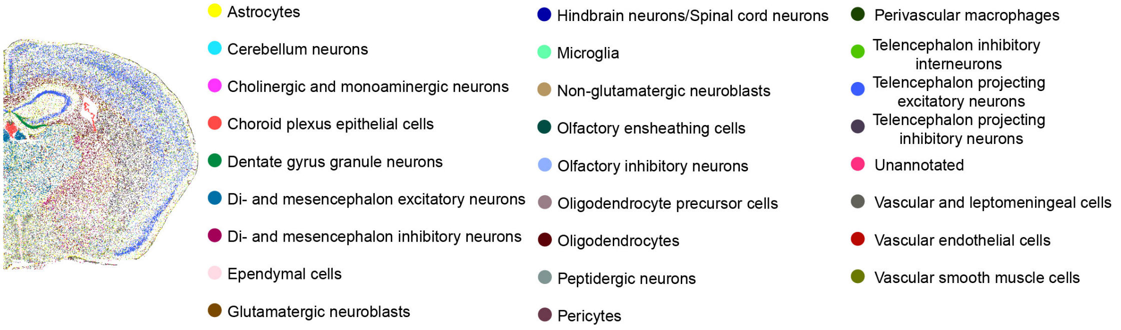

Supplementary Fig. 8: Spatial visualization of cell types in mouse brain slice "well11".

Cells are colored according to major cell types.

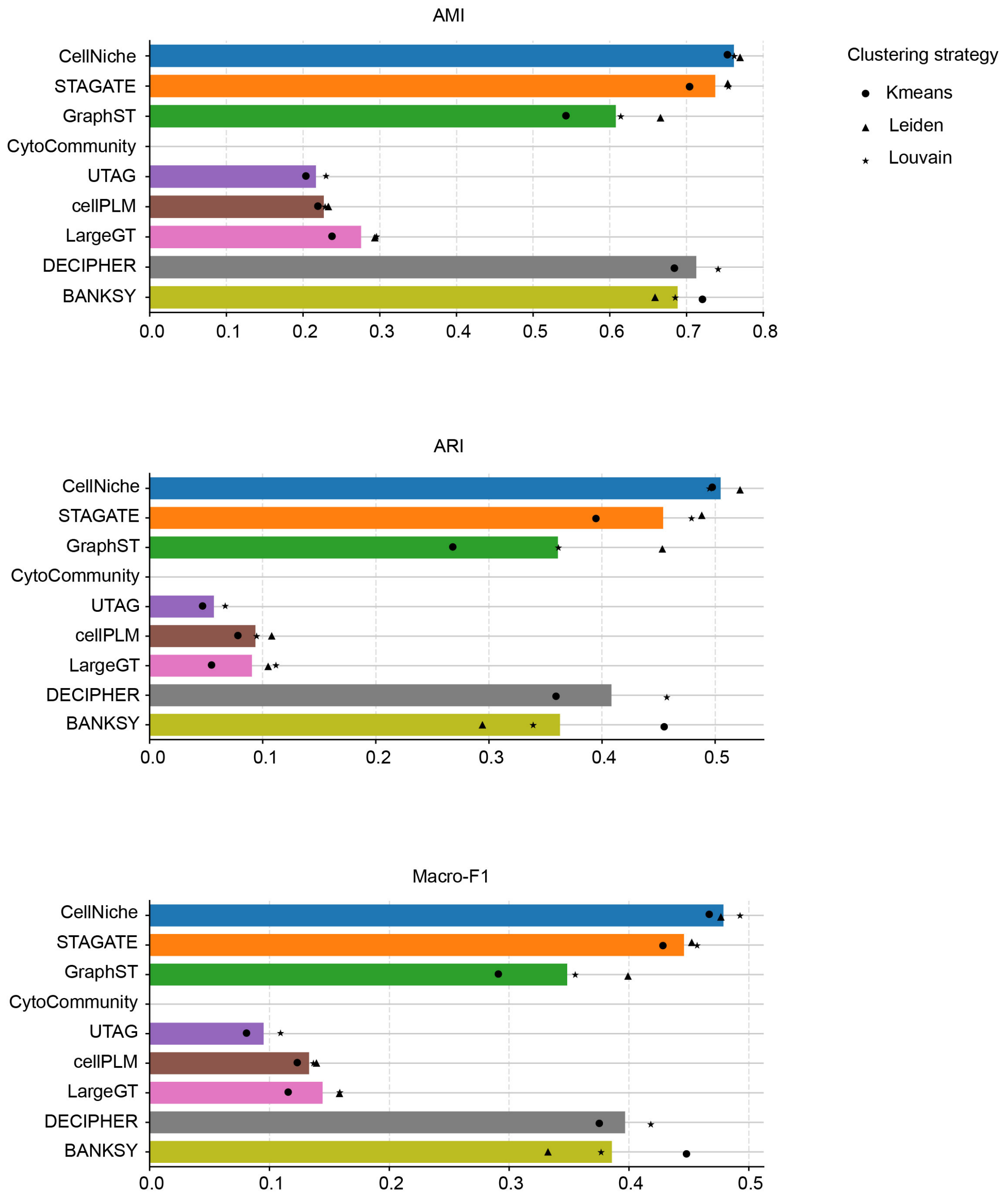

Supplementary Fig. 9: Benchmark performance under different clustering strategies on the STARmap PLUS mouse brain dataset (coronal slice "well11").

Bar plots show the average agreement with the reference region annotations across three clustering strategies (K-means, Leiden, and Louvain) for each method. Marker symbols indicate the scores obtained under each clustering strategy (circle: K-means; triangle: Leiden; star: Louvain). Metrics include AMI, ARI, and Macro-F1. Notably, the absence of a Leiden or Louvain marker for a specific metric indicates that the target number of clusters (matching the reference labels) could not be achieved, even after the resolution parameter was iteratively tuned to five decimal places. Source data are provided as a Source Data file.

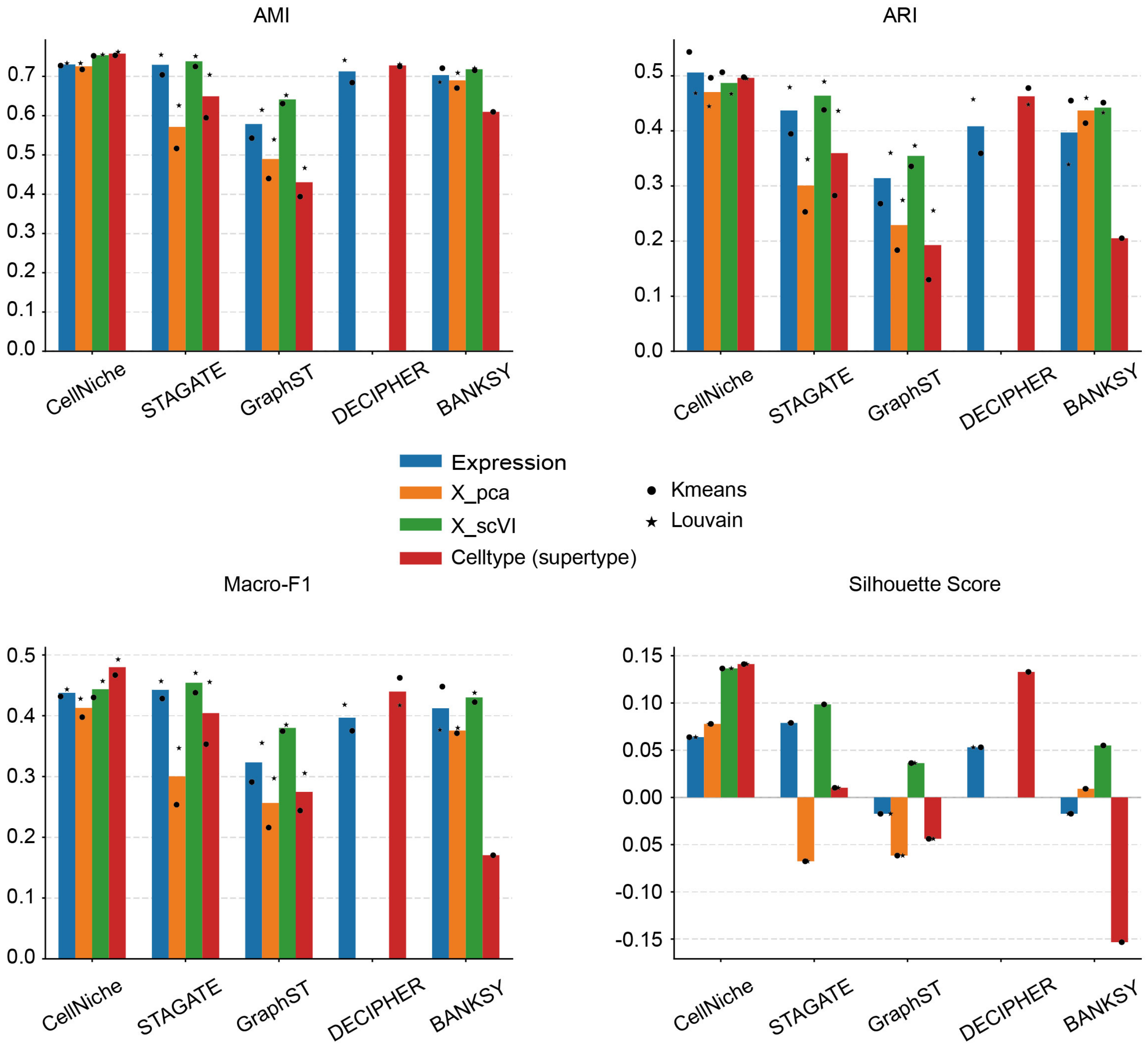

Supplementary Fig. 10: Benchmarking the performance of five spatial analysis methods under four distinct molecular identity representations on the "well11" coronal slice of the mouse brain (STARmap PLUS).

CellNiche, STAGATE, GraphST, DECIPHER, and BANKSY were evaluated using four types of input features: raw expression profiles (blue), PCA embeddings (orange), scVI embeddings (green), and "supertype" cell labels from the original study (red). Performance was assessed using four evaluation metrics—AMI, ARI, Macro-F1, and silhouette score—and two clustering strategies (K-means, black dots; Louvain, black stars). Source data are provided as a Source Data file.

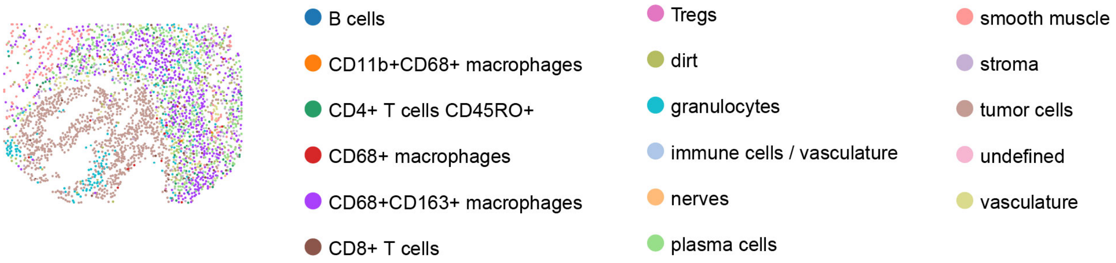

Supplementary Fig. 11: Spatial visualization of cell types in human colorectal cancer slice "reg007_A".

Cells are colored according to cell types.

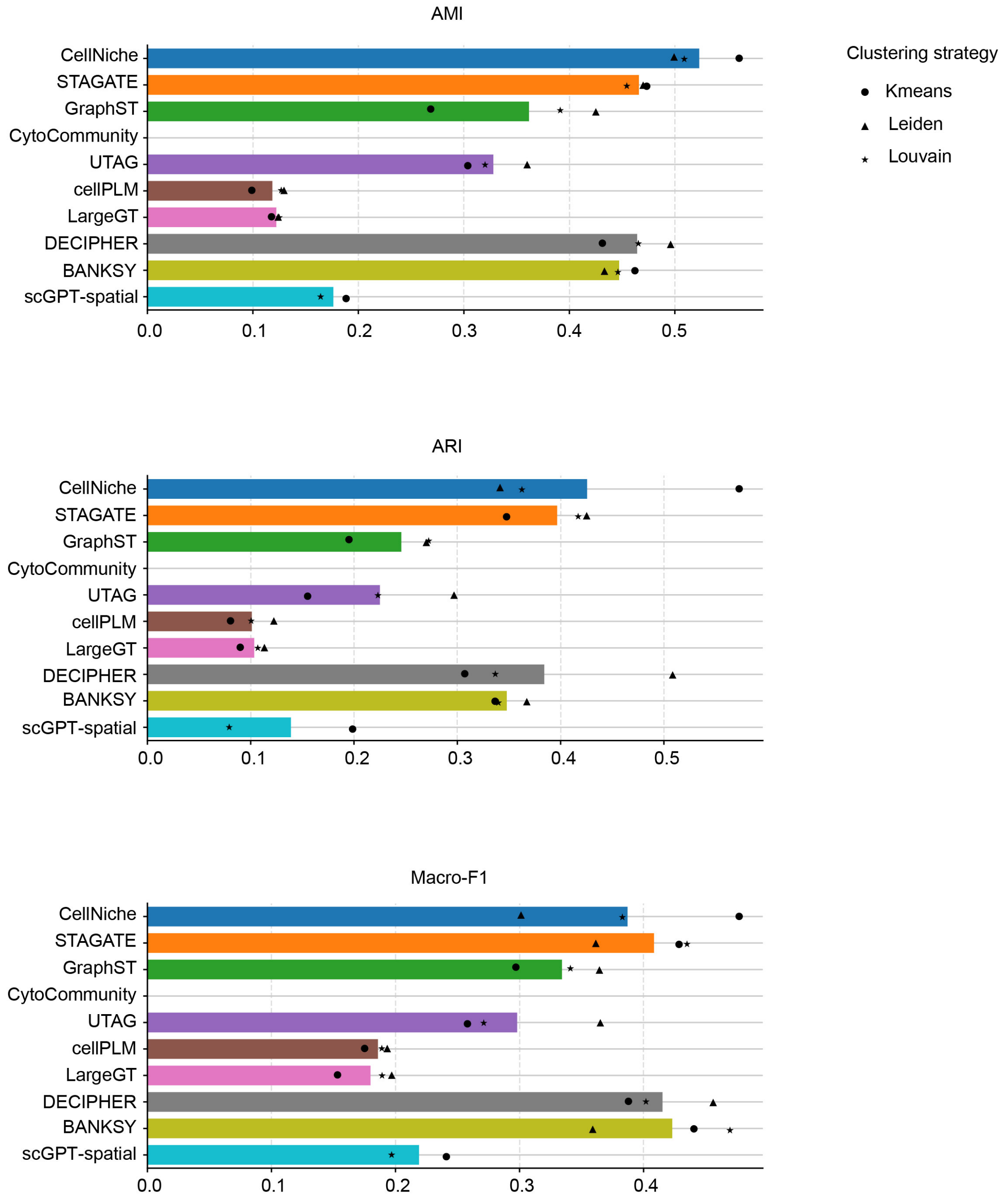

Supplementary Fig. 12: Benchmark performance under different clustering strategies on the human colorectal cancer CODEX dataset (slice "reg007_A").

Bar plots show the average agreement with the reference region annotations across three clustering strategies (K-means, Leiden, and Louvain) for each method. Marker symbols indicate the scores obtained under each clustering strategy (circle: K-means; triangle: Leiden; star: Louvain). Metrics include AMI, ARI, and Macro-F1. Notably, the absence of a Leiden or Louvain marker for a specific metric indicates that the target number of clusters (matching the reference labels) could not be achieved, even after the resolution parameter was iteratively tuned to five decimal places. Source data are provided as a Source Data file.

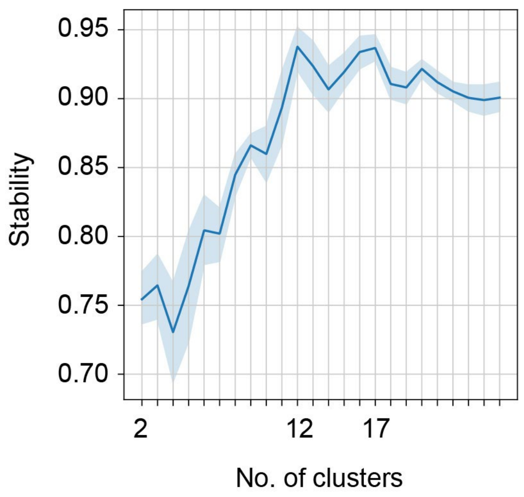

Supplementary Fig. 13. Cluster stability of microenvironment in NSCLC.

Cluster stability across $K = 2$–25. Stability is measured by the Fowlkes–Mallows index between clustering assignments at consecutive K values. Data are presented as mean values with a 95% confidence interval across $n = 5$ repeated runs.

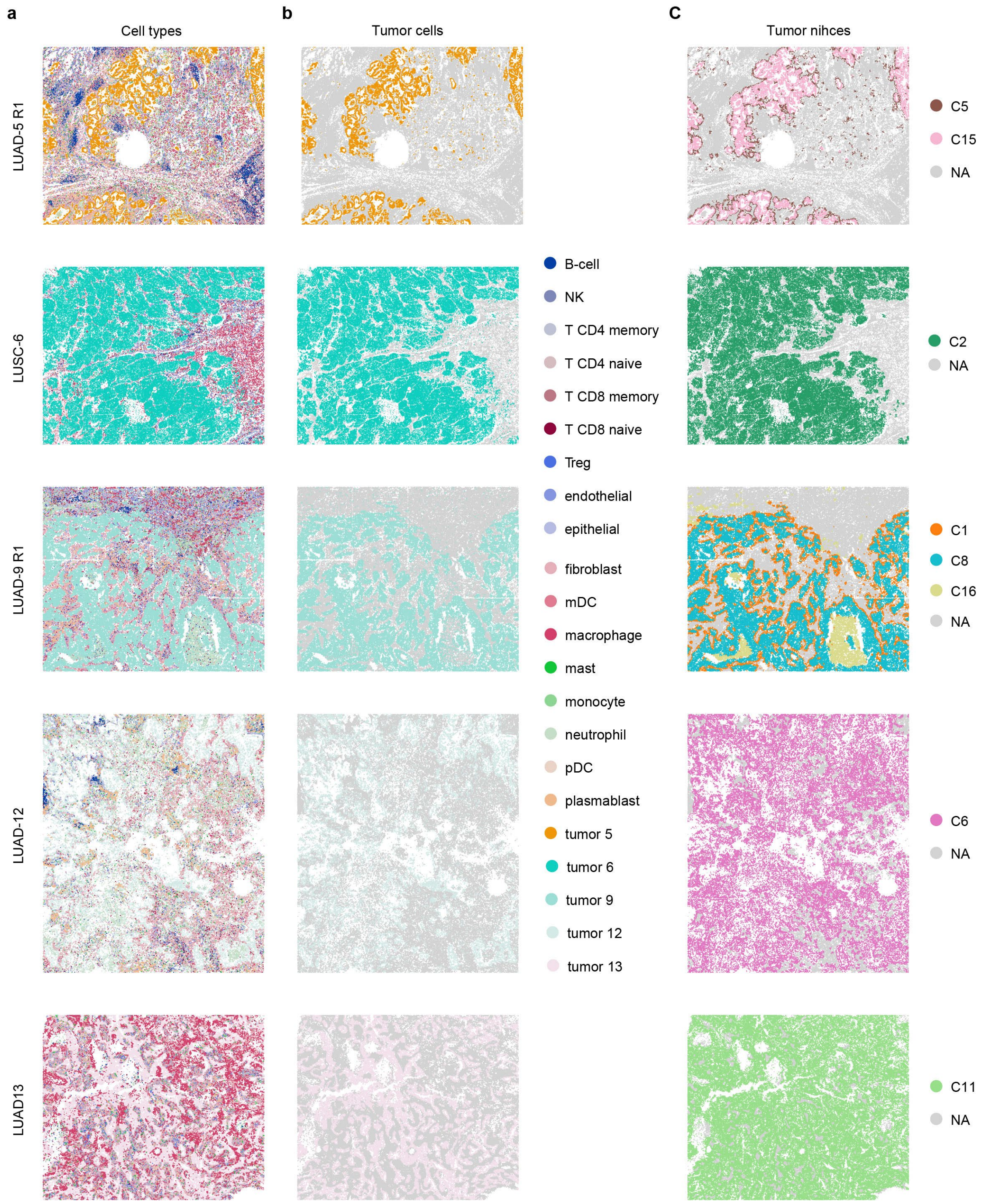

Supplementary Fig. 14: Spatial transcriptomics in NSCLC.

a, b, c, Spatial distribution of cell types (a), tumor cells (b), and tumor-enriched cell niches (c) across five sample slices.

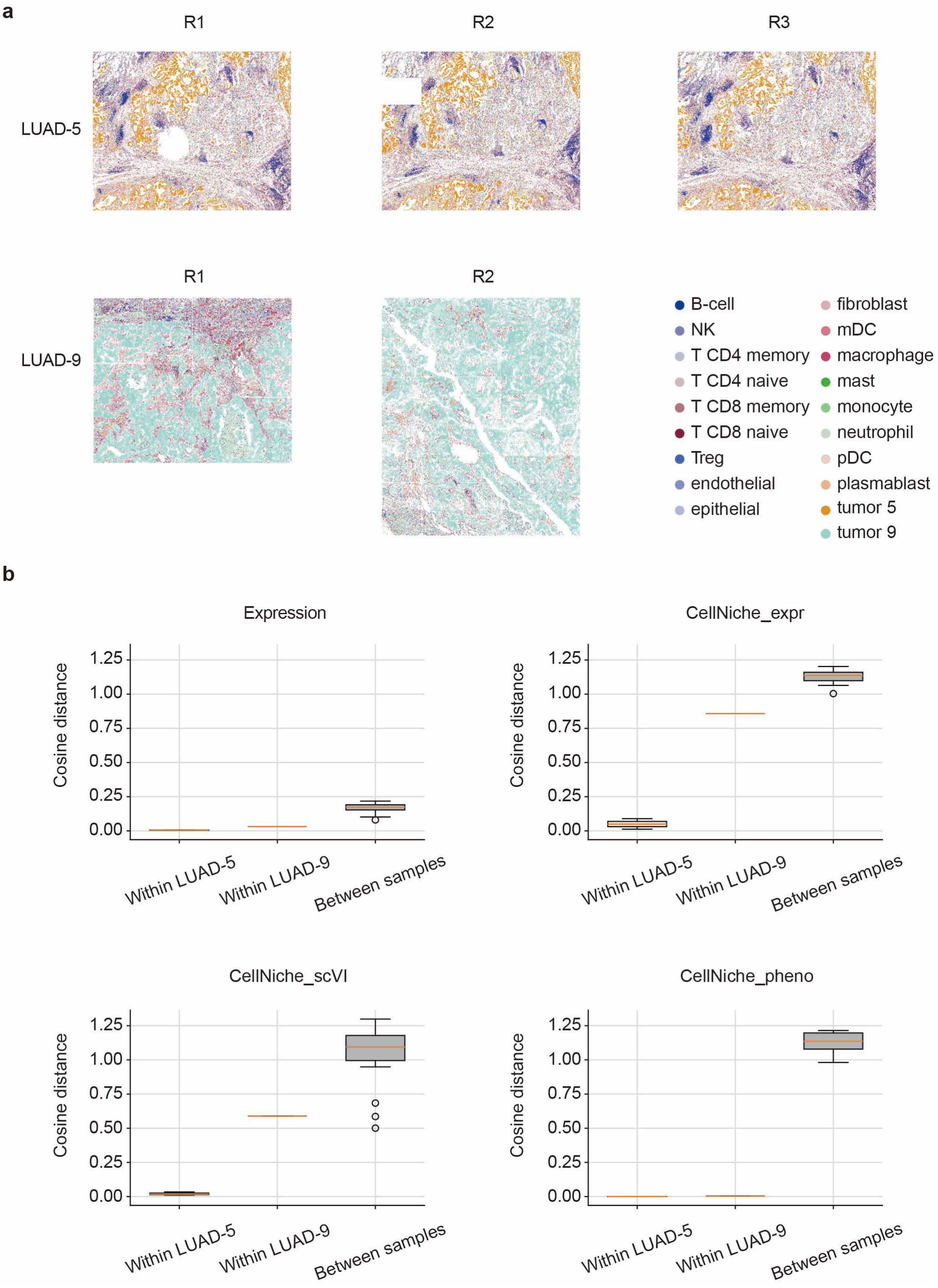

Supplementary Fig. 15: Spatial distribution and tumor microenvironment heterogeneity in NSCLC datasets.

a, Cell-type maps for LUAD-5 (three sections: R1–R3) and LUAD-9 (two sections: R1–R2). Each point represents a single cell, colored by its cell-type annotation (legend shown on the right). LUAD-5 sections exhibit highly consistent spatial tissue composition, while LUAD-9 sections display

noticeable spatial divergence. **b,** Cosine distances between tumor-cell centroid embeddings across sections, evaluated in four representation spaces: Raw expression, CellNiche trained with expression input (CellNiche_expr), CellNiche trained with scVI embeddings (CellNiche_scVI), and CellNiche trained with cell-type labels (CellNiche_pheno). For each embedding space, distances were computed for three groups: within LUAD-5 (three technical sections), within LUAD-9 (two technical sections), and between samples. Boxplots (minima, 25th percentile, median, 75th percentile, maxima, and outliers). The p value is calculated with the one-sided Mann-Whitney U test. n = 3, 1, 24 pairwise distances for the three groups, respectively. Source data are provided as a Source Data file.

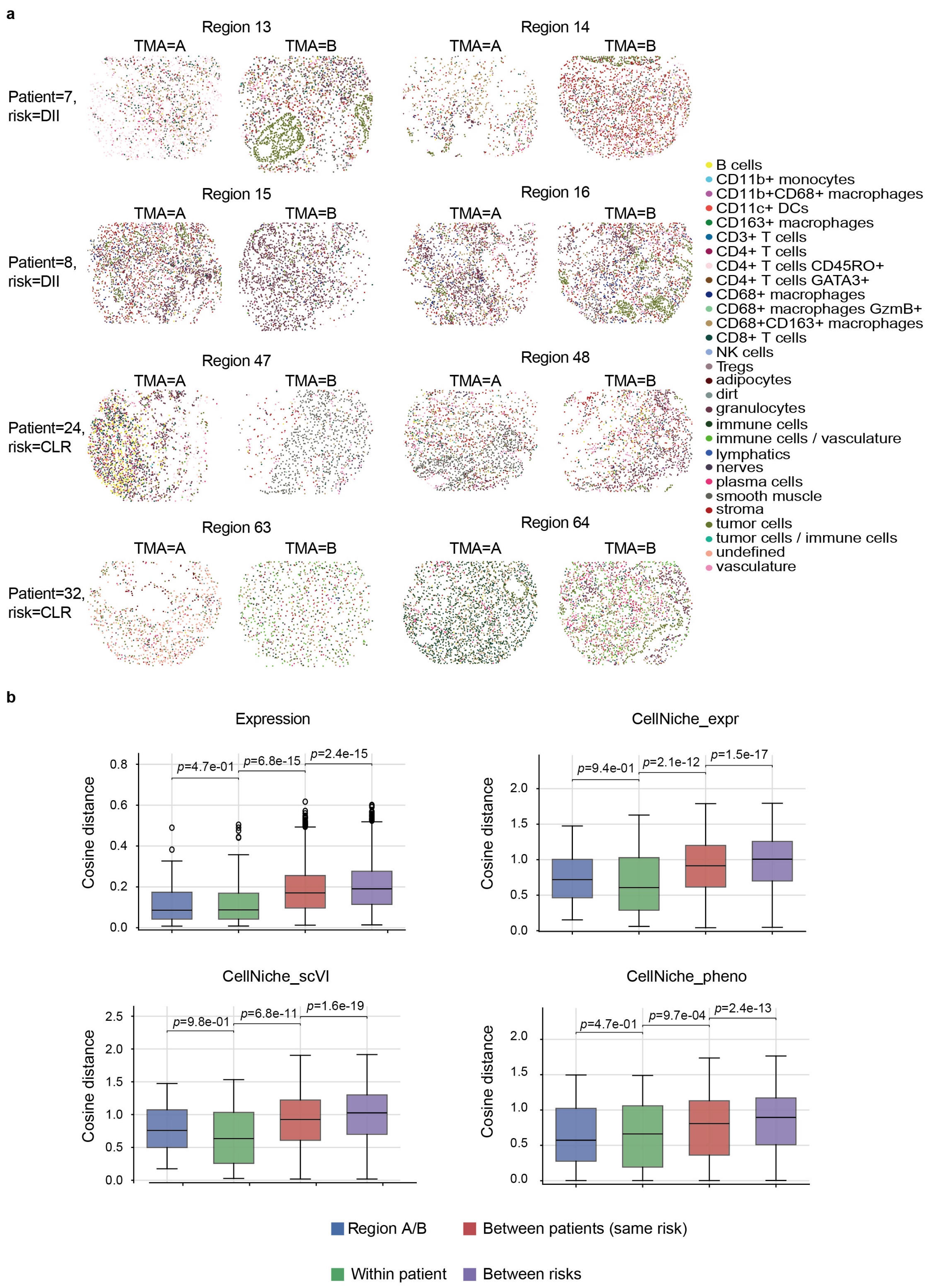

Supplementary Fig. 16: Spatial distribution and tumor microenvironment heterogeneity in CRC datasets.

a, Cell-type maps for representative tumor regions from CLR and DII patients. For each region, two adjacent sections (TMA-A and TMA-B) are shown. Each point represents a single cell, colored by its cell-type annotation (legend shown on the right). **b,** Cosine distances between tumor-cell centroid

embeddings across four representation spaces: Raw expression (RawX), CellNiche trained with expression input (CellNiche_expr), CellNiche trained with scVI embeddings (CellNiche_scVI), and CellNiche trained with cell-type labels (CellNiche_pheno). Distances were computed across four groups: (i) region A/B pairs from the same region ID, (ii) different regions within the same patient, (iii) different patients with the same CLR/DII risk classification, and (iv) patients with different risk classifications. Boxplots (minima, 25th percentile, median, 75th percentile, maxima, and outliers). The p value is calculated with the one-sided Mann-Whitney U test. n = 68, 136, 4,497, 4,752 pairwise distances for the four groups, respectively. Source data are provided as a Source Data file.

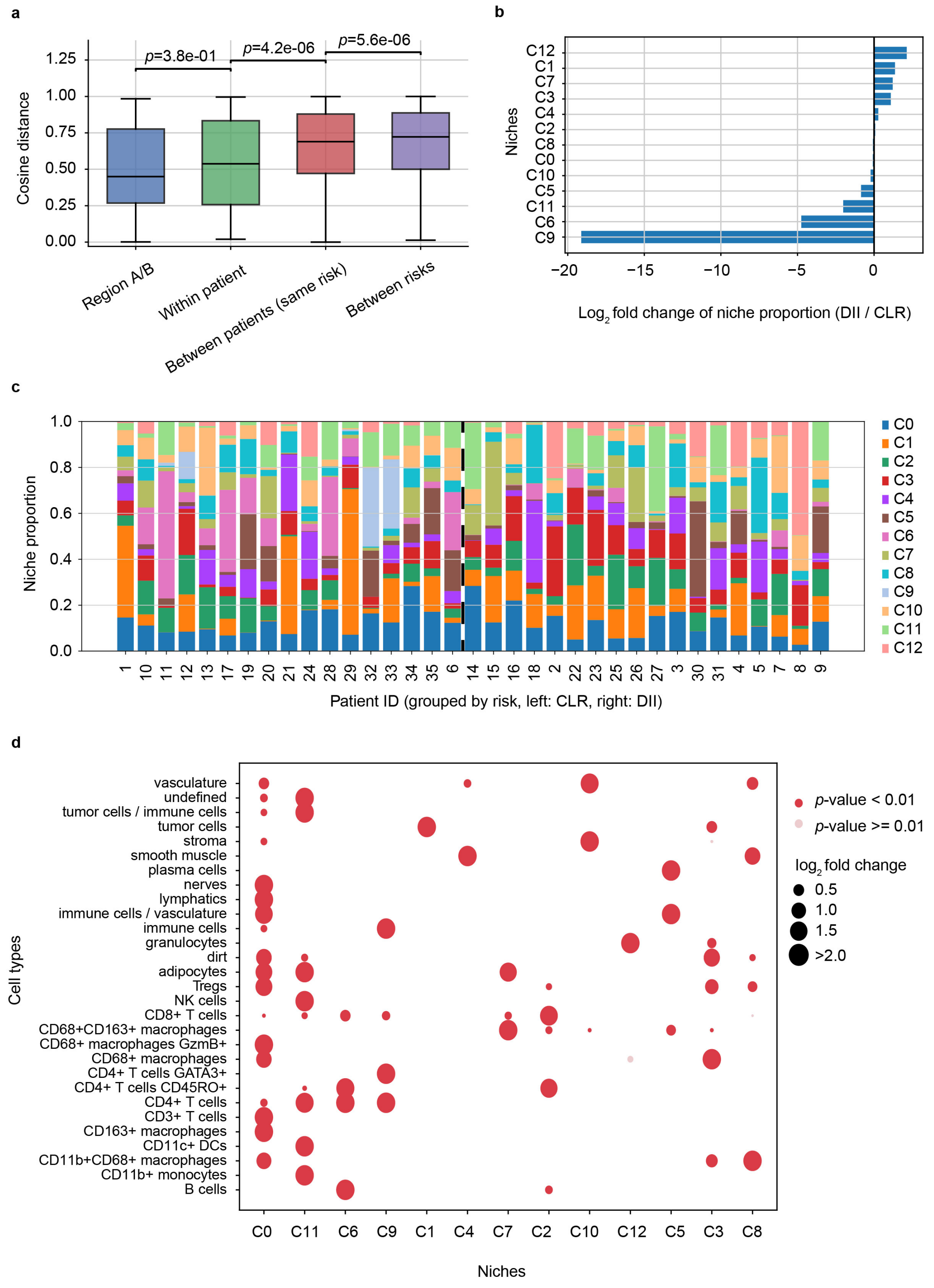

Supplementary Fig. 17: Characterization of CellNiche-defined microenvironmental niches in the human CRC cohort.

a, Cosine distances between patient/region niche-composition profiles derived from CellNiche in the CRC cohort. Each profile is a vector of niche proportions (C0–C12) for a given region or patient, and cosine distances were computed across four groups: (i) region A/B pairs from the same region ID,

(ii) different regions within the same patient, (iii) different patients with the same CLR/DII risk classification, and (iv) patients with different risk classifications. Boxplots (minima, 25th percentile, median, 75th percentile, and maxima). The *p* value is calculated with the one-sided Mann-Whitney U test. *n* = 70, 140, 4,624, 4,896 pairwise distances for the four groups, respectively. **b,** Risk-associated niche shifts summarized by the $\log_2$ fold-change of patient-level niche proportion (DII/CLR) for each niche (C0–C12), highlighting niches preferentially represented in DII or CLR patients. **c,** Patient-level niche compositions visualized as stacked bar plots (C0–C12). Patients are grouped by clinical risk (left: CLR; right: DII), with the dashed line demarcating the two risk groups. **d,** Cell type enrichment in each niche (*n* = 13 niches). Source data are provided as a Source Data file.

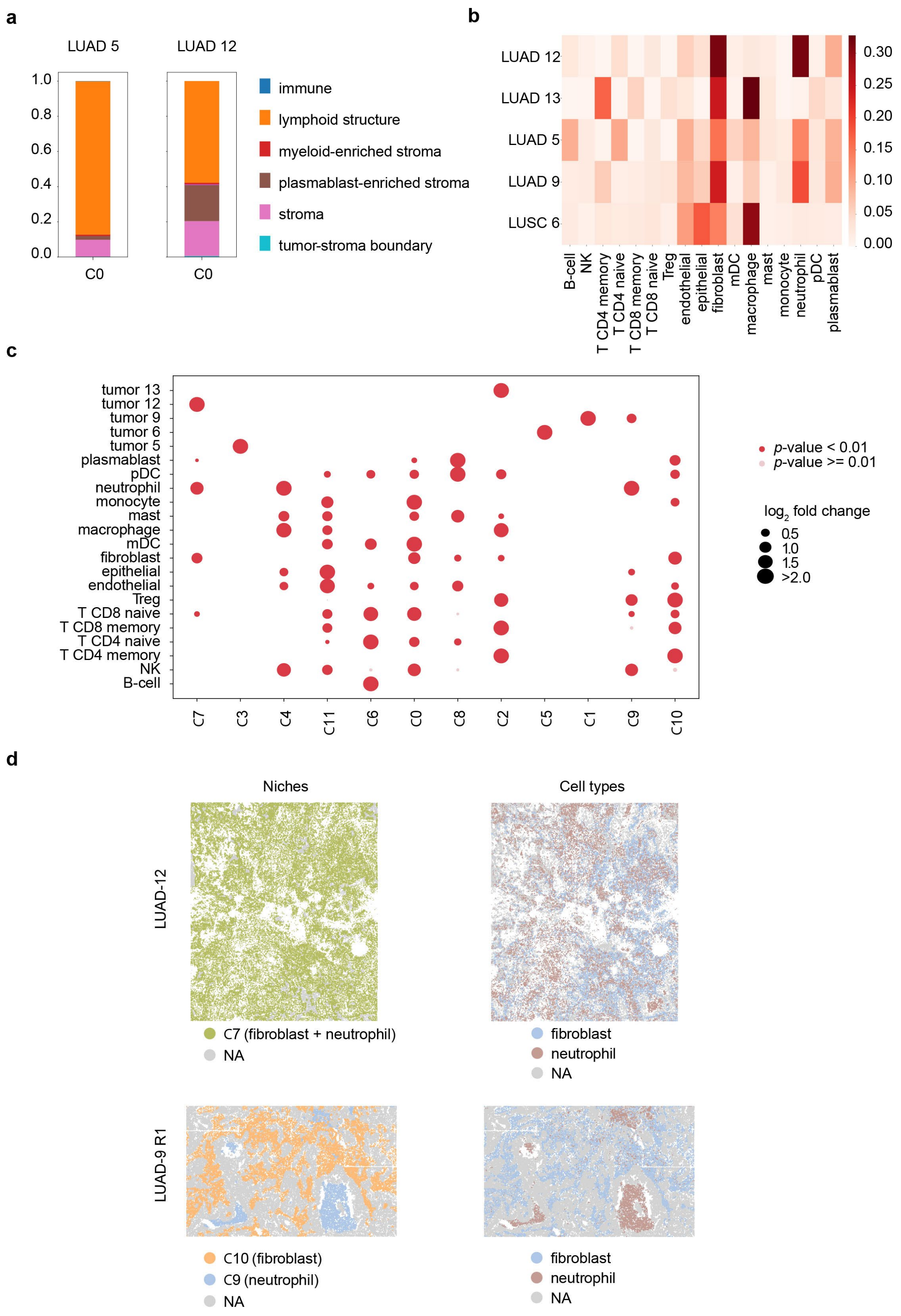

Supplementary Fig. 18. Additional analyses for NSCLC niche identification and characterization. a, The composition of the niches annotated by the original study in C0 of the CosMx human NSCLC dataset. **b,** Immune and stromal cell type fractions in tumor samples from 5 non-small cell lung cancer

patients (n = 12). **c,** Cell type enrichment in each niche (n = 12 niches). Dot size denotes $\log_2$(fold change) and color indicates significance (empirical p values from a permutation test). Significant enrichment is defined as $p < 0.01$. **d,** Spatial distribution of fibroblast- and neutrophil-enriched niches (left), spatial distribution of fibroblast and neutrophil (right), visualized for LUAD-9 R1 (bottom) and LUAD-12 (top).

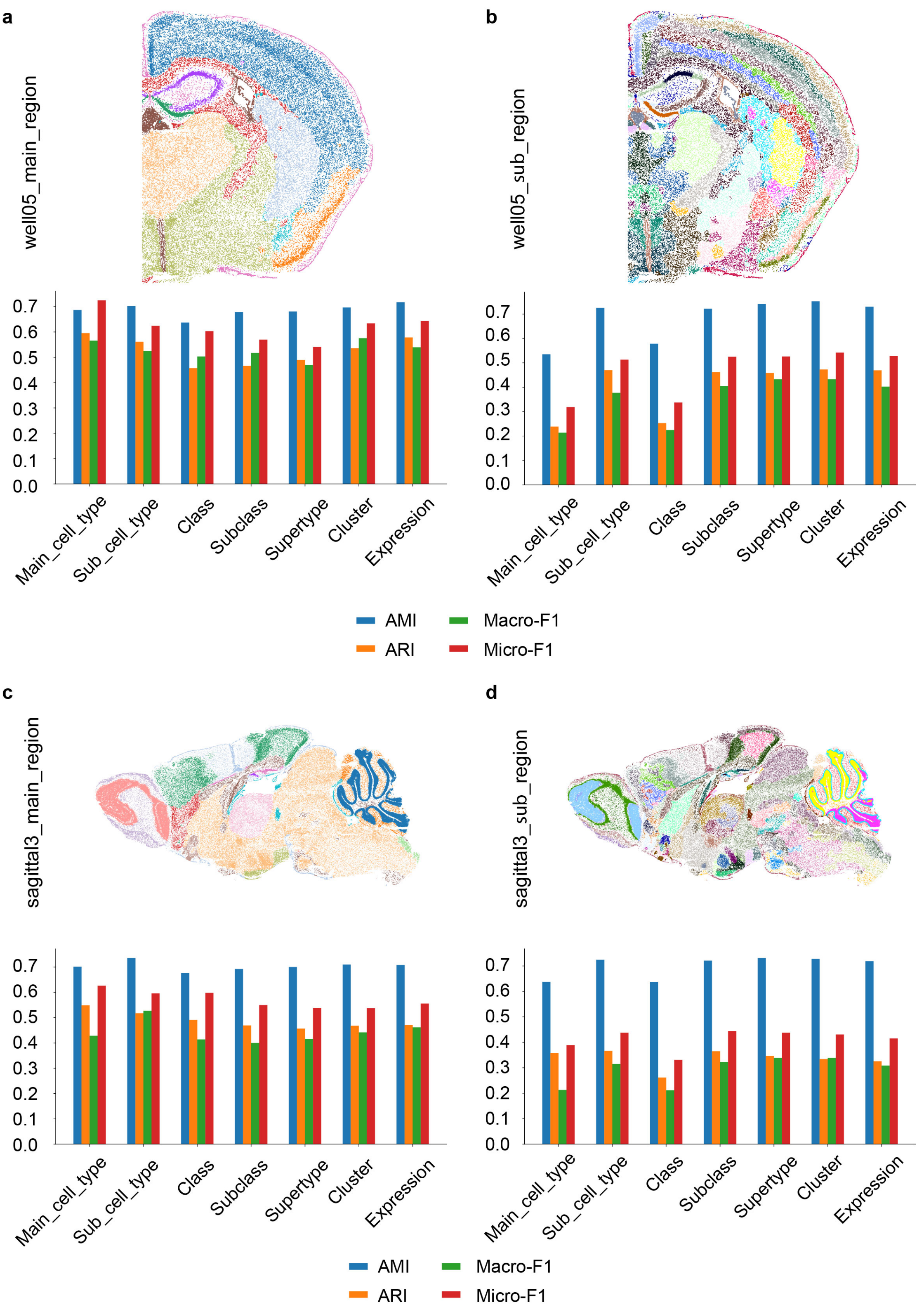

Supplementary Fig. 19: Impact of Input Resolution on Tasks with Different Resolution Levels.

a, b, Evaluation of main-tissue region (a) and sub-tissue region (b) identification on Atlas 1 "well05" using model inputs at different resolution levels, including primary cell types, subclass cell types, and four-level cell type annotations from the Allen Institute. Performance is measured using AMI, ARI, Macro-F1, and Micro-F1. **c, d,** Evaluation of main-tissue region (c) and sub-tissue region (d)

identification on Atlas 1 "sagittal3" using model inputs at different resolution levels, including primary cell types, subclass cell types, and four-level cell type annotations from the Allen Institute. Performance is measured using AMI, ARI, Macro-F1, and Micro-F1. Source data are provided as a Source Data file.

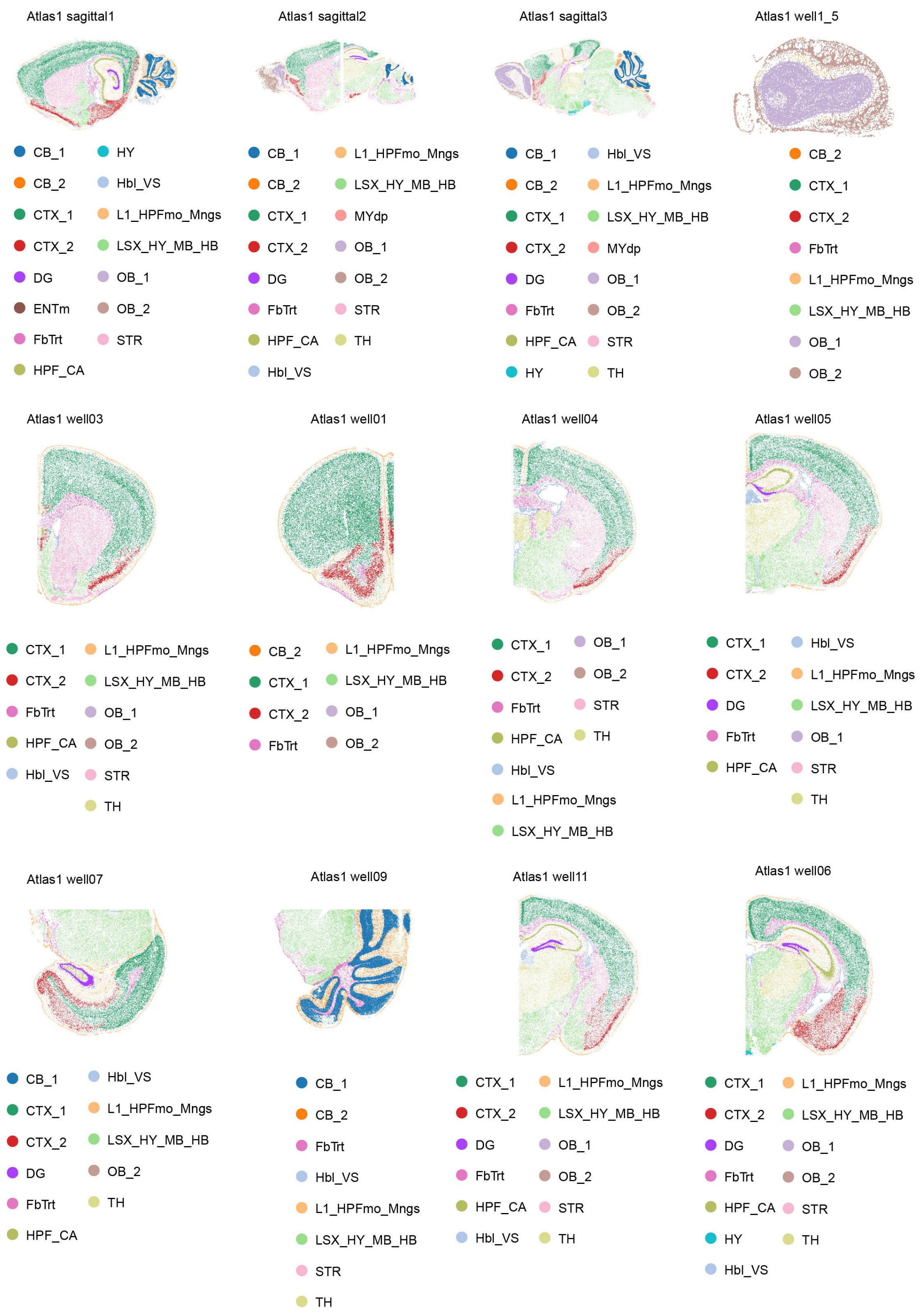

Supplementary Fig. 20: Spatial visualization of transferred main-tissue annotations for representative sections of Atlas 1.

Cells are colored according to their transferred main-tissue annotations.

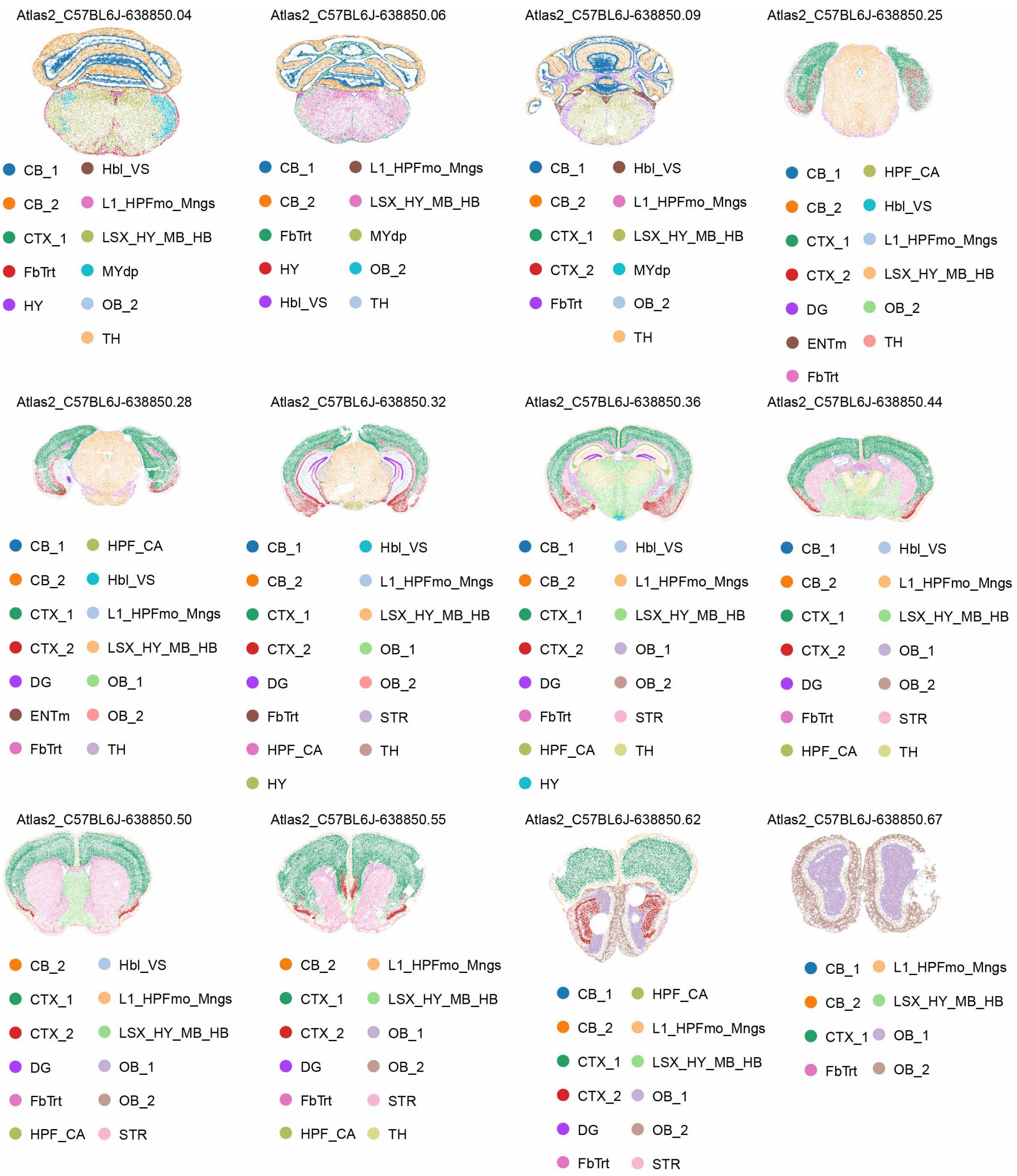

Supplementary Fig. 21: Spatial visualization of transferred main-tissue annotations for representative sections of Atlas 2.

Cells are colored according to their transferred main-tissue annotations.

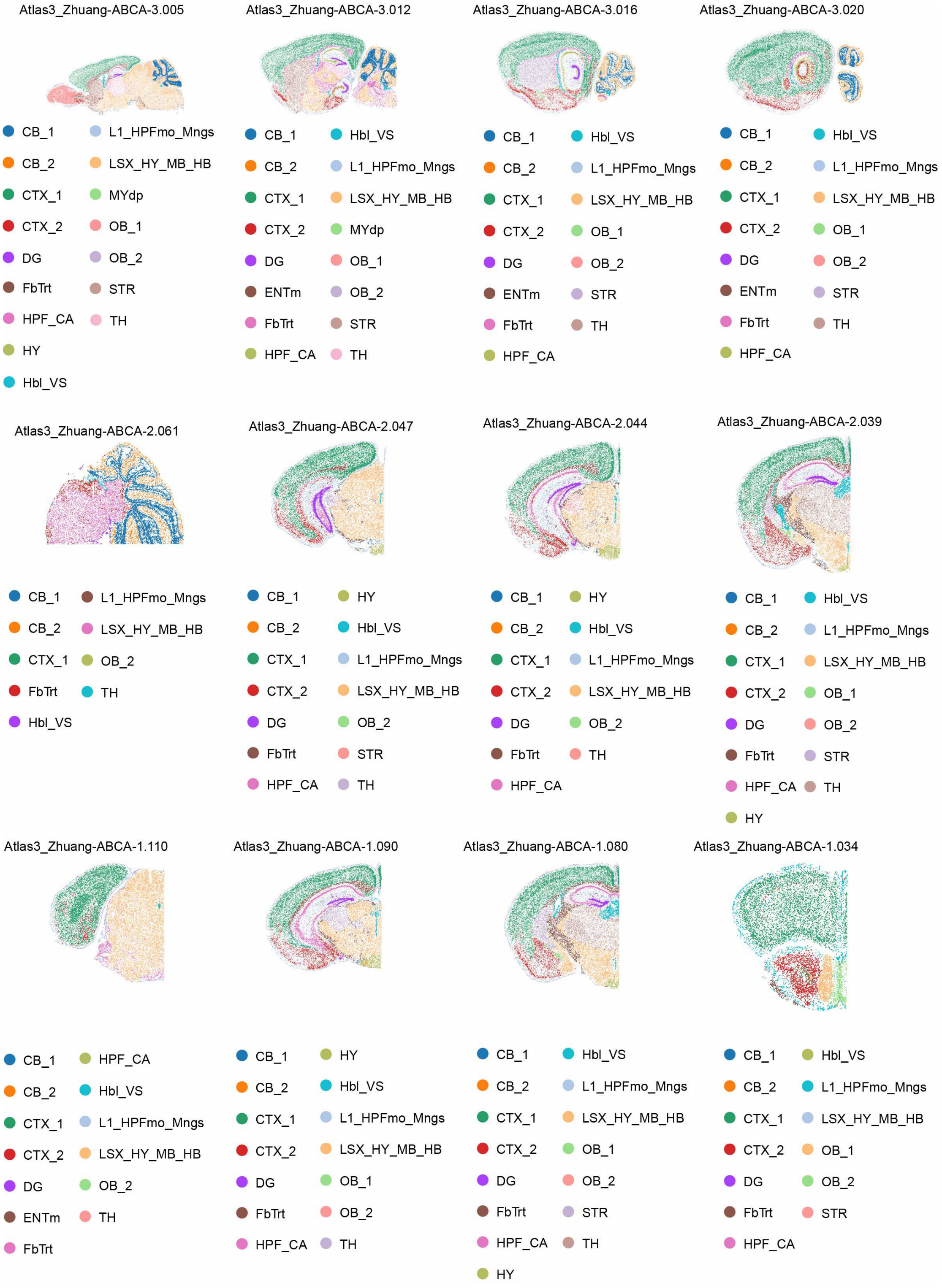

Supplementary Fig. 22: Spatial visualization of transferred main-tissue annotations for representative sections of Atlas 3.

Cells are colored according to their transferred main-tissue annotations.

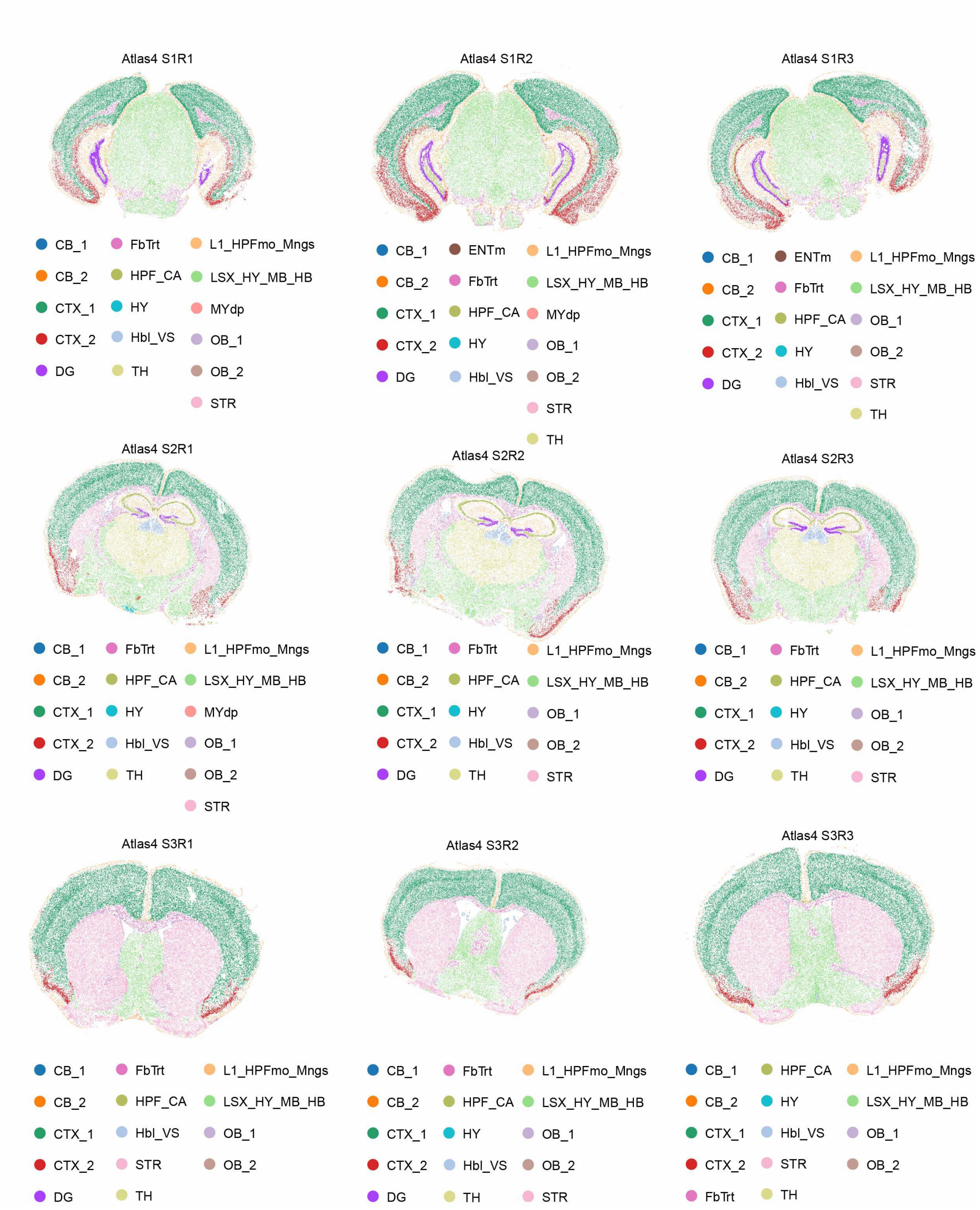

Supplementary Fig. 23: Spatial visualization of transferred main-tissue annotations for representative sections of Atlas 4.

Cells are colored according to their transferred main-tissue annotations.

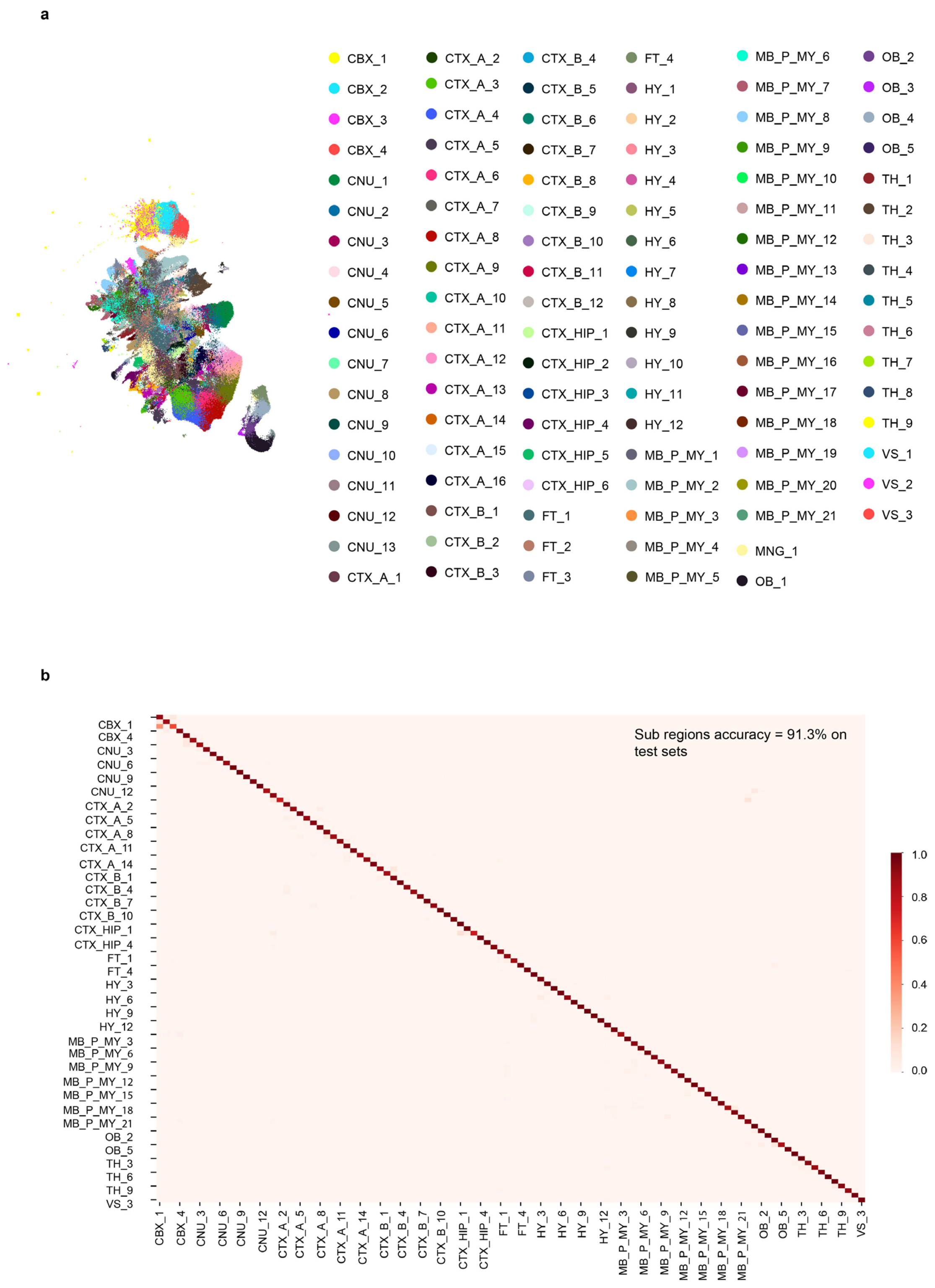

Supplementary Fig. 24. Sub-tissue annotation transfer in the integrated mouse brain atlas.
a, UMAP projections of cell embeddings generated by CellNiche, colored by transferred sub-tissue annotations. **b,** Percentage of original tissue region annotations (rows) from Atlas 1 transferred to sub-level tissue region annotations (columns).

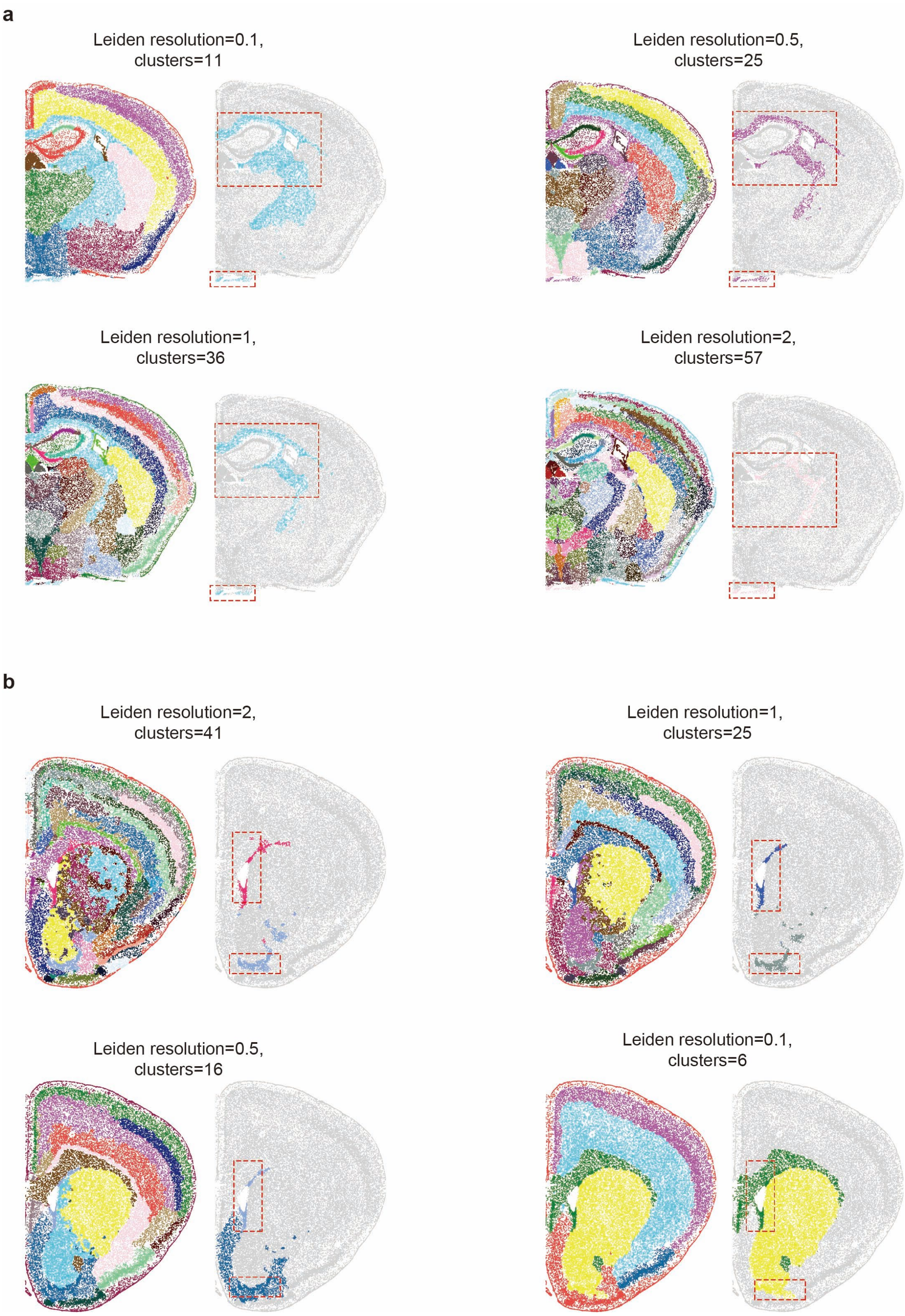

Supplementary Fig. 25: Robustness of the FbTrt refinement and the CNU_13 bipartition across Leiden resolutions.

a, Leiden clustering was performed on CellNiche embeddings for the Atlas 1 well05 section, using a range of resolution parameters (as indicated). Left panels show the full Leiden partition; right panels

highlight the cells assigned to FbTrt by cross-atlas label transfer. Across resolutions (from coarse to fine partitions), FbTrt-labeled cells consistently form a coherent and spatially contiguous domain. **b,** Leiden clustering on CellNiche embeddings was evaluated across multiple resolutions for the Atlas 1 well03 section to test the stability of the proposed CNU_13 subdivision. Even under coarse clustering (few global clusters), the two CNU_13 subdomains remain separable in the embedding-derived partitions.
