# Supplementary material for: CellNiche represents cellular microenvironments in atlas-scale spatial omics data with contrastive learning
Source: Nat Commun. 2026 Apr 22;17:5547. doi: 10.1038/s41467-026-71759-4 (PMC13287597; doi:10.1038/s41467-026-71759-4)
Supplement: Supplementary file 3 — Transparent Peer Review file [file 41467_2026_71759_MOESM3_ESM.pdf]

nature portfolio

Peer Review File

CellNiche represents cellular microenvironments in atlas-scale spatial omics data with contrastive learning

Corresponding Author: Mr Zhongming Liang

This file contains all reviewer reports in order by version, followed by all author rebuttals in order by version.

Version 0:

Reviewer comments:

Reviewer #1

(Remarks to the Author)
CellNiche is a contrastive-learning based computational tool which identifies and characterizes cellular microenvironement from large-scale spatial transcriptomic data. CellNiche was applied to spatial transcriptomic and proteomic datasets spanning six different platforms, demonstrating both its scalability and performance. In addition, the framework was applied to a multi-sample human non-small cell lung cancer cohort, where they identified tumor-associated and immune spatial microenvironments. Finally, CellNiche was used to integrate mouse brain slices from four independent atlases, enabling the construction of a comprehensive virtual brain map. More information is required to fully understand the methodology, and several aspects of the work could be strengthened. The following comments highlight areas where clarification or improvement would be beneficial:
Major Comments:
1. Authors claim that the one main contribution of this manuscript is to decouple molecular identity representations from spatial proximity. If this is true, then it is a great contribution. However, I didn't see any convincing results about this point.
2. To strengthen the discussion of the CellNiche's scalability, it would be valuable to provide additional details regarding the factors that contribute to its efficiency. Further clarification is needed on whether the observed speedup is due to the loss function, the contrastive learning strategy, architecture, or the batch sampling procedure. Audience may be interested in knowing which term make it so fast. Furthermore, more details are needed for memory consumption in the scalability analysis, specify the number of A6000 GPUs employed during training.
3. In reference to analyzing the efficiency/scalability of CellNiche, I guess CellNiche_pheno model is used in the scalability section. If it is the case, it may not be appropriate when compared to other methods since CellNiche_pheno takes cell labels as inputs and other methods do not.
4. The use of cell labels for CellNiche_pheno in benchmarking accuracy may not be appropriate, as it could artificially inflate accuracy and does not fully reflect the model's performance compared to other methods which do not use methods. Also, a clear description of what "cell phenotype information" is and how is it obtain is required.
5. The osmFish spatial transcriptomics dataset used has only 33 genes for performance evaluation. To more rigorously assess the generalizability and robustness of the proposed method, it would be important to evaluate its performance on larger-scale spatial transcriptomics datasets with higher gene coverage.
6. Relying solely on k-means–based evaluation may be problematic, as it is not a direct evaluation of the embedding quality and it also depend on the clustering. A more comprehensive assessment could be achieved by comparing the embeddings directly against the ground-truth cell labels using metrics such as the silhouette index.
7. A clear description of the model's output and the procedure by which spatial microenvironments (cell niches) are identified is needed, particularly in the overview of the Results section.
8. The section on NSCLC where cell-cell communication is used is not explained clearly, and the analysis of tertiary lymphoid structures (TLSs) on C0 requires further elaboration. It is not clear what is the consequence of two niche was predominantly present in two samples. For C4 analysis, it is unclear why only two cell type labels (B cell and Cd4) are emphasized and how this choice reflects biological realism. Moreover, the description does not fully align with the corresponding figure (Fig. 4e), which creates confusion.
9. In the final results section, further details are needed to explain why additional Leiden clustering was performed and how it supports the claim of subregion refinement of slice Zhuang-ABCA-1.069 (Atlas 3). The rationale for applying this extra clustering step should be clearly articulated. Additionally, what is referred to as Figure 8 appears to correspond to Figure 7b.

Minor Comments:

1. In Methods section, additional details are needed regarding how Gene Set Variation Analysis (GSVA) and Differential Gene Expression (DGE) were performed.
2. The implementation details for Harmony and FuseMap should be added to the Methods.
3. Information on cluster stability assessment should be provided rather than solely citing previous work.
4. Figure 4.d,e, and h need proper x-axis and y-axis labeling. Additionally, the description of Figure 4h in the figure legend should be clarified.
5. GraphSAGE should be cited in the training module section of Methods.
6. Figure 7e appears to be missing.
7. Figure S3 may correspond to Figure S6; this should be clarified.
8. A clearer definition of tertiary lymphoid structures (TLSs) is needed, along with an explanation of their relevance to this analysis.
9. Figure 3d is not referenced and the information on evaluation of the STARmap dataset using ARI, NMI, Macro-F1, and Micro-F1 metrics is missing.
10. The introduction should include the application of the NSCLC dataset and describe how the biological validation is planned.
11. The term “walk” used in the methodology should be clearly explained for clarity and reproducibility.

(Remarks on code availability)

Reviewer #2

(Remarks to the Author)
The authors propose a neural-network approach based on contrast learning to identify and characterize cellular microenvironments from spatial omics data. The authors show their approach performs better and faster compared to current approaches for numerous datasets. A few comments:

1. Although it’s infeasible to compare with every single other method, the authors should try to compare with the most popular recent methods, and those identified to be high-performing in benchmark papers. E.g., BANKSY (https://www.nature.com/articles/s41588-024-01664-3) and top methods in https://link.springer.com/article/10.1186/s13059-024-03361-0 – “Benchmarking clustering, alignment, and integration methods for spatial transcriptomics“. The last link also details methods which have good scalability, including BANKSY, Dr.SC, SpatialPCA, Spaceflow and PRECAST. Don’t have to implement all, but perhaps the ones which have both good scalability and high performing, e.g., BANKSY and SpatialPCA. It would also be good if the authors use the same datasets in the BANKSY/Spatial PCA and comparison papers, for fair comparison.
A similar comment applies to the integration methods chosen for Figure 6 – the authors only chose Harmony and FuseMap. It will be good if the authors either justified their choice (e.g., is there a recent review paper which showed FuseMap or Harmony was a top performing method?) or consider other recent methods, e.g., STaligner (https://www.nature.com/articles/s43588-023-00528-w), SPIRAL (https://link.springer.com/article/10.1186/s13059-023-03078-6), graphST, etc..

2. The authors indicate that batch effects is mitigated because the input are cell type labels, which makes sense and is fine. However, it’s not clear whether there will be batch effects when the input is expression profiles, which is discussed in the loss function module.

3. Parts of the text can be made more clear/grammatical mistakes, e.g.,
• The “strategies” in Figure 1a was a bit confusing, as I didn’t see how it matched with those in the text. E.g. the text talks about “batch sampling” “constrastive learning” and other uses of the word “strategy”.
• Line 70 – can’t a cells’ function be determined by other omics, e.g., proteins?, not just gene expression
• Line 83 – the connection between hypothesis-drive and data-drive paradigms, and spatial coordinates/structural constraints and neural networks can be made more clear. Similar comment for line 114 – the “hypothesis drive modelling basd on spatial..” wasn’t that clear to me. Also first letter of “hypothesis” should be capitalized.
• Page 1, line 37, “microenvironment, Inspired”
• Is Figure 8b a typo? (line 401)

4. It will be useful if the authors give more detail of the different cellNiche variants (perhaps in the Methods), and which scenarios should they be used. E.g., the authors indicate that there are scenarios when CellNiche_expr is the best-performing.

(Remarks on code availability)
Haven't installed, but their Github page seems to have detailed instructions for installing and running the application. There are also numerous tutorials.

Reviewer #3

(Remarks to the Author)

Summary:
This manuscript presents CellNiche, a scalable contrast learning framework for identifying and characterizing cellular microenvironments from spatial omics data. When applied to over 10 million cells spanning 6 distinct spatial omics platforms, the framework demonstrated superior performance compared to state-of-the-art methods, alongside robust atlas-scale scalability and batch-correction capabilities. While CellNiche holds promise for advancing spatial microenvironment analysis, the manuscript requires improvements in several aspects, including methodological contextualization, result validation, and content accuracy. Detailed comments are as follows:

Major comments:
1. The manuscript claims that the tool enables the analysis of large-scale heterogeneous spatial multi-omics data—a functionality already offered by existing tools such as DECIPHER (also published in Nature Communications). Notably, key capabilities highlighted for the proposed tool, including “model large-scale heterogeneous spatial data in silico effectively and efficiently”, “a novel cross-scale contrast learning strategy”, “enable delineating cell environment interaction across multiple scales” and “capable of handling spatial atlases with millions of cells” are also core features of DECIPHER. However, the manuscript lacks a systematic comparison between the two tools. Please supplement this critical contextualization:
- A clear comparison of core features (eg: core functions and details for model construction) to clarify its novelty;
- Quantitative evaluations of “runtime efficiency”, “performance” and “biological interpretation in real-world data” across datasets of varying sizes and types, to empirically demonstrate your tool’s performance advantages;
- A detailed discussion of model design differences. Both your tool and DECIPHER use contrastive learning to reduce runtime and propose its ability on large-scale datasets, but the manuscript fails to elaborate on distinctions in the model design—and how these differences impact efficiency and analytical outcomes.

2. Also, in the benchmark analysis, the manuscript only includes GNN methods (e.g., STAGATE, GraphST) and some Transformer-based methods (e.g., CellPLM, LargeGT), but omits other large-model studies in the current spatial omics field (such as scGPT-spatial, the spatial extension of scGPT mentioned in the manuscript). Such large models typically exhibit stronger cross-dataset generalization and feature extraction capabilities, making them important competitors to CellNiche for atlas-level data analysis. Without comparisons with these methods, the performance advantages of CellNiche in large-scale, heterogeneous data cannot be fully validated. We suggest that the authors supplement comparative experiments with mainstream spatial omics large models like scGPT-spatial, using consistent evaluation metrics (e.g., ARI, AMI, Macro-F1) and datasets. This will verify CellNiche’s competitiveness in the context of large models while analyzing its relative advantages in computational efficiency and microenvironment identification accuracy.

3. The manuscript presents three versions of CellNiche (CellNiche_pheno, CellNiche_expr, CellNiche_decoder), but their performance lacks consistent superiority (e.g., the default CellNiche_pheno performs best on the osmFISH dataset, while CellNiche_expr performs best on the CODEX dataset). We recommend that the authors conduct an in-depth analysis or discussions of the reasons for performance differences across versions and provide clear guidelines for version selection (e.g., which version is preferred for gene expression data, and which is suitable for phenotypic annotation data).

4. Clustering is primarily conducted using the K-means algorithm, where the number of clusters is arbitrarily set to match the number of annotated regions. No justification is provided for this choice—for instance, a rigorous biological prior hypothesis is required and how should the clustering number be determined for samples without prior knowledge (also the number is related with different biological label resolutions, how to determine the exact number of clustering)? We suggest:
- Testing alternative clustering algorithms widely used in single-cell/spatial omics (e.g., Leiden, Louvain, just like the results present in Fig. 7);
- Comparing cluster quality across methods using metrics like silhouette score or adjusted Rand index (ARI) to validate the reliability of region segmentation and ensure downstream analysis is robust.

5. Figure 4 presents inter-patient tumor heterogeneity, but it fails to distinguish whether this heterogeneity arises from “tissue section sampling differences” (e.g., spatial location within the tumor) or “intrinsic sample differences” (e.g., patient-specific genetic background or tumor stage). R1-R3 from the same sample LUAD5 may be adjacent sections and tend to have similar distributions of niches. Meanwhile, the current analysis relies on a small number of samples from the same type of cancer and without pan-cancer coverage, making it difficult to support the claim that the tool “reflecting interpatient heterogeneity in tumor composition.” We suggest:
- Incorporating more samples from the same type of cancer/pan-cancer datasets (e.g., breast cancer, colorectal cancer) to expand sample diversity and discussing the potential explanations of the heterogeneity between location differences and sample differences;
- Conducting analyses (if have, e.g., comparing samples from different patients with matched sampling locations, or comparing multiple sections from the same patient) to clarify the source of heterogeneity and enhance the generalizability of results.

6. The manuscript provides no explanation of how the tool analyzes or compares data at different granularities (e.g., cell subtype-level, cluster-level). Nor does it interpret the connections or differences between results at these granularities. Please supplement:
- Strategies for converting data between granularities, especially when addressing and comparing datasets with different label resolutions (which resolution should we choose and how to maintain the robustness of comparisons);
- A comparative interpretation of analytical outcomes across granularities for the same dataset, to demonstrate the tool’s flexibility in multi-scale analysis.

7. When comparing different methods, the manuscript should use the original datasets reported in the respective methods. This introduces bias, as dataset characteristics can influence method performance. We recommend adhering to standard benchmarking practices:
- Use the “native validation datasets” from each compared method’s original study;
- Alternatively, well-recognized public spatial multi-omics benchmark datasets are also acceptable (e.g., human non-small cell lung cancer dataset) to ensure fair comparisons, while more recent methods should be included.

8. There are many typos or wrong statements in the current manuscript:
- The claim that “One notable microenvironment, C0, showed strong enrichment for lymphoid-like cells... and was predominantly present in LUAD-5 and LUSC-6 (Fig. 4b)” is inconsistent with the data in Figure 4b. Please verify and correct this. Not LUSC-6?
- “CellChat analysis predicted strong MHC-I signaling interactions between CD8 naïve T cells and other cell types within cluster C4 (Fig. 4d)” is incorrect; the interactions actually occur in cluster C0. Correct the cluster ID and figure annotation.
- The statement “Similar trends were observed under coarser clustering resolutions... (Fig. 5a, 5d, 5e)” cannot be validated, as no corresponding results exist in Figure 5! Either add the missing figures or revise the conclusion.
- “Similarly, we successfully transferred 107 subregion annotations with 91.3% accuracy (Fig. 7e, Fig. S6a, b)” contains an incorrect figure citation; Figure 7e does not exist. Verify and update the reference.
- “Additional Leiden clustering (41 clusters) further confirmed the subregion refinement (Fig. 8b)” is wrong; the relevant result is in Figure 7b, not 8b. Correct the figure number.
- Thoroughly check and revise the entire manuscript, including but not limited to the errors above.

9. Figures 7a and 7b show marker gene expressions across different tissue sections, but no explanation is provided for why “different marker genes are used for similar anatomical regions across sections”. If these regions are intended to be similar, justify the use of distinct markers, and clarify the biological rationale for marker selection.

(Remarks on code availability)
The code provides a detailed README file with instructions for installing and running the application. I can easily install the code.

Version 1:

Reviewer comments:

Reviewer #1

(Remarks to the Author)
The additional analyses have significantly improved the manuscript, and the authors have addressed most of the questions. I have a few comments.
• The statement that CellNiche “decouples molecular identity representations from spatial proximity modeling” may be overly definitive. It would be more precise to describe how these two components are integrated or parameterized. Complete decoupling could lead to biologically inconsistent results; for instance, if two spatially proximal spots exhibit distinct expression profiles, their classification into the same or different niches should logically depend on their molecular identities. A model that strictly decouples these features might fail to capture the nuanced interdependence required to define a niche accurately.
• The manuscript does not mention the use of Louvain clustering method in the main text, although these analyses are presented in Supplementary Figures 2–5. It is highly recommended to add it in Methods section.
• The analyses for supplementary figures 6 and 7 has not been described or integrated into the manuscript, despite the presence of related results.
• Several other subfigures and supplementary figures are not explicitly referenced in the main text. All figures and supplementary materials should be cited and discussed to guide the reader through the results.
• Further clarification of the CRC dataset (around line 275) would be beneficial.
• In Figure 4f, cluster C17 is missing. Please verify whether this is a typographical or labeling error and correct it accordingly.
• Figure 4h lacks x- and y-axis tick labels. These should be added to improve interpretability.

(Remarks on code availability)

Reviewer #2

(Remarks to the Author)
I thank the authors for the response.

1) I thank the authors for including the additional spatial omics methods, but I couldn’t get an indication of how these methods compare with their method based on their response. In addition, as previously suggested, to ensure the authors are not cherry-picking data, it would be useful to also show the performance of their method on datasets which previous methods have shown to be best-performing.

2) Thanks for clarifying the batch effects issue. First, the manuscript would be more clear if there is consistency with how "batch" is used, as the definition of "batch" in the paper seems confusing, as the authors talk about "batch sampling", "batch processing", "microenvironment batch", and "batch effects"? Does "batch" mean the same thing in all these cases? It'll be good if the authors make it explicitly clear somewhere. Second, the authors should indicate in the response what changes were made from the previous version (e.g., old version vs new version). Eg., in the previous version they talk about Cell Niche "effectively mitigating batch effects for cross-dataset analysis", and now they talk about "batch effects…handled flexibly upstream". This seems like a conceptual shift, and it's not clear if the authors have changed their method in the revised version to accommodate this change, or are simply now presenting a different interpretation of batch effects in relation to their method.

3) Regarding the cellNiche variants, as with the previous comment, it would be useful (and appropriate) if the authors mention any changes they made from the original submitted manuscript in the reviewer response. For example, the previous Figure 2 had figures showing different variants of celNiche (eg., pheno, expr, decoder), but now they are not shown in the current updated figure. It's not clear what the reason for this and what the authors are now showing in their paper (Eg., pheno, expr or decoder?), making it hard to judge whether they have addressed the comments.

(Remarks on code availability)

Reviewer #3

(Remarks to the Author)
The authors have addressed all major concerns raised in my initial review. A few minor suggestions for further improvement are noted below:
1. Related to my previous comment #5, I could not find the CellNiche's spatial niche proportions for samples of CRC cohort. Whether the results are consistent with the tumor microenvironment differences across region, patient, and risk groups.
2. Please carefully check the order and citations of all supplementary figures in the main text.
3. Missing titles for supplementary figures 12 and 15.

(Remarks on code availability)

Version 2:

Reviewer comments:

Reviewer #1

(Remarks to the Author)
All my comments are addressed, and I have no further comments.

(Remarks on code availability)

Reviewer #2

(Remarks to the Author)
I thank the authors for the detailed response, and am happy to recommend the paper for publication.

(Remarks on code availability)

Reviewer #3

(Remarks to the Author)
The authors have addressed most of the questions. A few minor comments for further improvement are noted below:
The ordering of Supplementary Figure in the main text is incorrect: subfigure 11a is positioned before subfigure 10, and subfigure 14b precedes subfigure 14a.

(Remarks on code availability)

Open Access This Peer Review File is licensed under a Creative Commons Attribution 4.0 International License, which permits use, sharing, adaptation, distribution and reproduction in any medium or format, as long as you give appropriate credit to the original author(s) and the source, provide a link to the Creative Commons license, and indicate if changes were made.
In cases where reviewers are anonymous, credit should be given to 'Anonymous Referee' and the source.
The images or other third party material in this Peer Review File are included in the article’s Creative Commons license, unless indicated otherwise in a credit line to the material. If material is not included in the article’s Creative Commons license and your intended use is not permitted by statutory regulation or exceeds the permitted use, you will need to obtain permission directly from the copyright holder.
To view a copy of this license, visit https://creativecommons.org/licenses/by/4.0/

Point-by-point response

Reviewer #1 (Remarks to the Author):

CellNiche is a contrastive-learning based computational tool which identifies and characterizes cellular microenvironement from large-scale spatial transcriptomic data. CellNiche was applied to spatial transcriptomic and proteomic datasets spanning six different platforms, demonstrating both its scalability and performance. In addition, the framework was applied to a multi-sample human non-small cell lung cancer cohort, where they identified tumor-associated and immune spatial microenvironments. Finally, CellNiche was used to integrate mouse brain slices from four independent atlases, enabling the construction of a comprehensive virtual brain map. More information is required to fully understand the methodology, and several aspects of the work could be strengthened. The following comments highlight areas where clarification or improvement would be beneficial:

Response:

Thanks for the precise summary and help to strengthen our work!

Major Comments:

1. Authors claim that the one main contribution of this manuscript is to decouple molecular identity representations from spatial proximity. If this is true, then it is a great contribution. However, I didn't see any convincing results about this point.

Response:

We sincerely thank the reviewer for raising this key and insightful point. We agree that the relationship between molecular identity and spatial proximity lies at the core of spatial microenvironment modeling. We are glad the reviewer recognizes our decoupling design as a great contribution in improving performance robustness, handling batch effects, enabling large-scale spatial integration and allowing practical flexibility. We appreciate the opportunity to clarify both the conceptual motivation to decouple molecular identity representations from spatial proximity and the empirical evidence supporting our design.

1 Rationale for Decoupling Molecular Identity from Spatial Modeling

In the single-cell transcriptomics era, encoder–decoder frameworks trained via expression reconstruction have been highly effective for extracting and denoising high-dimensional, sparse, and noisy molecular signals. This approach has played a central role in learning meaningful cellular representations directly from raw expression profiles.

Building upon this paradigm, most existing spatial omics models (e.g., STAGATE, GraphST)

jointly learn molecular identity and spatial structure through reconstruction-driven training. However, coupling molecular feature compression with spatial interaction modeling introduces two key limitations:

1.1 High computational cost driven by reconstruction objectives.

Reconstruction-based training requires iterative optimization toward recovering high-dimensional molecular profiles, typically necessitating substantially more training epochs, increased memory usage, and longer runtimes before meaningful representations emerge. As spatial datasets scale to millions of cells, this computational burden becomes a major constraint.

1.2 Limited adaptability to diverse or externally defined molecular representations.

Methods that focus on learning or extracting cell identity generally assume that raw expression profiles—being noisy and sparse—require transformation into a latent space. Consequently, these models are inherently tailored to raw transcriptomics data and are less compatible with alternative or pre-computed representations, such as PCA, scVI embeddings, protein-derived features, or curated cell-type annotations. As the field moves toward unified reference embeddings enabled by large-scale consortia and foundation models, a tightly coupled reconstruction paradigm may become increasingly misaligned with emerging use cases.

In contrast, CellNiche is built on the hypothesis that molecular identity can be treated as a fixed representation space, and that spatial microenvironments can be modeled independently—without re-learning or reconstructing molecular features. This reflects a paradigm shift in which spatial interaction embedding becomes the sole learning target, separating identity representation from spatial modeling to improve efficiency, flexibility, and future compatibility.

2 Implementation of the Decoupled Design

To operationalize this decoupled paradigm, CellNiche is designed so that the model:

2.1 Does not optimize or reconstruct molecular features as a learning objective.

While CellNiche employs a graph encoder to generate a latent embedding, this transformation is not trained to compress, refine, or recover molecular identity. Unlike reconstruction-based frameworks, the encoder serves solely to support the spatial contrastive objective rather than to learn a new molecular representation.

2.2 Optimizes exclusively for spatial relational structure.

The training objective acts on pairwise relationships defined by microenvironmental patterns of co-localization and co-expression, adjusting the embedding space such that relational structure within local spatial neighborhoods is emphasized. In this framework,

contrastive learning operates on relational signals, rather than explicitly redefining or extracting molecular identity.

2.3 Treats molecular identity as a fixed input space.

CellNiche does not attempt to infer or transform identity into a new molecular feature space. Instead, it assumes the input representation—whether raw expression, PCA, scVI embeddings, protein-derived features, or cell-type annotations—to be a valid identity space and learns spatial interaction structure within that fixed space.

Together, these design choices ensure that CellNiche learns microenvironment-driven spatial relationships rather than molecular identity itself, distinguishing it from approaches that jointly couple molecular feature learning and spatial interaction modeling within a single training objective.

3 Performance Robustness Across Diverse Molecular Identity Representations

We conducted a new benchmarking analysis on the coronal slice *"well11"* of the mouse brain generated using STARmap technology. In this analysis, we considered four distinct forms of cellular identity features as model inputs: (i) raw expression profiles, (ii) PCA embeddings, (iii) scVI embeddings, and (iv) "supertype" labels provided by the original study.

We compared CellNiche against four high-performing spatial omics methods—STAGATE, GraphST, DECIPHER, and BANKSY—using four evaluation metrics (AMI, ARI, Macro-F1, and Silhouette score) and two clustering strategies (K-means and Louvain).

Across all four input representations, CellNiche consistently achieved the highest or comparable performance, whereas the other methods exhibited varying degrees of sensitivity to the choice of molecular representation. In particular:

when replacing raw expression with PCA features (X_pca), STAGATE and GraphST exhibited substantial performance drops; in contrast, their performance rebounded markedly when using scVI embeddings (X_scVI). This pattern suggests that their quality of the learned microenvironment representation is strongly influenced by the upstream embedding choice.

BANKSY showed comparatively stable performance across raw expression, X_pca, and X_scVI—on par with CellNiche in terms of robustness to these continuous molecular inputs—but its performance deteriorated sharply when switching to discrete cell-type labels, indicating limited adaptability to phenotype-only representations.

DECIPHER, which is also trained with a contrastive objective, showed good compatibility with both the raw expression matrix and the cell-type－based representation. However, when X_pca or X_scVI embeddings were used as input, DECIPHER failed to produce meaningful embeddings. One plausible explanation is that its self-comparison strategy relies on generating paired augmented views of the same cell based on dropout; in low-dimensional or sparse feature

spaces (PCA is 50-dimensional by default, and scVI is 10-dimensional), this operation may produce information-poor augmented views, leading to training failure. Similar issues were observed on the mouse spleen CODEX proteomics dataset, which contains only 29 protein tags.

Taken together, these results suggest that, compared with existing methods, CellNiche is less dependent on any specific form of molecular representation and can operate robustly across diverse identity feature spaces. This empirical behavior is consistent with our design choice to treat molecular identity as a fixed input space rather than an explicit learning target within the model.

4 Decoupling Enables Superior Scalability

Because CellNiche treats molecular identity as an input space rather than a learning target, the model does not require iterative optimization toward reconstructing high-dimensional molecular profiles. Consequently, it can obtain informative microenvironment representations within comparatively fewer training epochs, resulting in a reduced computational burden and improved scalability on large spatial datasets.

5 Decoupling Simplifies Batch Handling and Integration

Since identity representations are not re-learned within the model, batch correction can be applied flexibly at the upstream stage using a wide range of standardized or externally defined approaches—such as scVI-derived embeddings, unified cell-type annotations, or potentially future large-model–based cellular representations—rather than being embedded as an additional module within the spatial model itself. This design enables CellNiche to operate on batch-corrected inputs directly, without requiring task-specific adjustments or model-level modifications.

Building on this principle, we applied CellNiche to two settings in the manuscript where a unified cellular identity space was available: (i) integration and analysis of a multi-sample NSCLC cohort, and (ii) cross-platform integration of mouse brain atlases.

In both cases, training was performed using standardized cell-type labels as the input identity representation, allowing data from different samples or platforms to be modeled jointly. These results further demonstrate the practical flexibility and utility of this decoupled design for handling batch effects and enabling large-scale spatial integration.

While these results do not exhaust all possible batch scenarios, they provide initial empirical evidence supporting the flexibility of this approach

We have now added these points in **Abstract, Introduction, Results section 1** and **Supplementary Figs. 6** of our revised manuscript.

Abstract section (Additions) (Line 41):

“

Importantly, CellNiche decouples molecular identity from spatial modeling by treating cellular identity features (e.g., expression-derived features, learned embeddings, or standardized cell-type annotations) as fixed inputs rather than reconstruction targets, enabling spatial characterization of cell–cell relationships without re-learning cellular identity representations.

”

Introduction section (Additions) (Line 126):

“

(2) CellNiche decouples molecular identity representations from spatial proximity modeling. Instead of learning molecular identity as a reconstruction target within the spatial model, CellNiche treats cellular identity features as predefined inputs (e.g., expression-derived features, learned embeddings, or standardized cell-type annotations). This enables the model to focus on learning microenvironmental relationships under fixed molecular identities, while allowing batch handling and identity harmonization to be performed flexibly upstream using established approaches. CellNiche then quantifies cell–cell relationships within each local niche using spatial co-localization frequencies and molecular similarity cues and optimizes context-aware embeddings via contrastive learning[21,22].

”

Results section 1 (Additions) (Line 153):

“

*More generally, CellNiche can take alternative predefined identity features (e.g., expression-derived features, learned embeddings, or standardized cell-type annotations), allowing batch effects to be handled flexibly upstream without modifying the spatial model (**Fig. 1a**, strategy 2).*

”

Supplementary Fig. 6 (Additions):

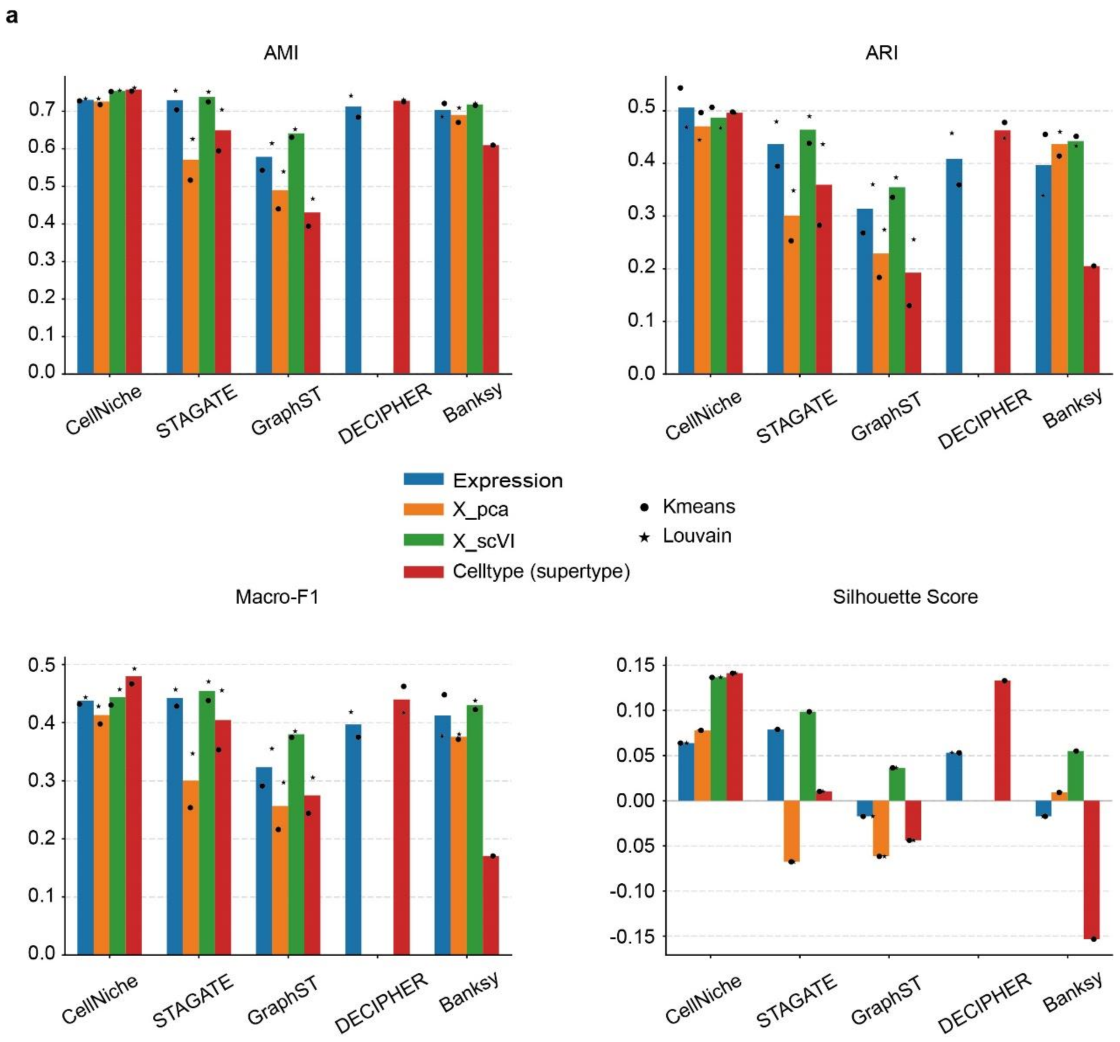

Supplementary Fig. 6: Benchmarking the performance of five spatial analysis methods under four distinct molecular identity representations on the "well11" coronal slice of the mouse brain (STARmap).

CellNiche, STAGATE, GraphST, DECIPHER, and Bansky were evaluated using four types of input features: raw expression profiles (blue), PCA embeddings (orange), scVI embeddings (green), and "supertype" cell labels from the original study (red). Performance was assessed using four evaluation metrics—AMI, ARI, Macro-F1, and silhouette score—and two clustering strategies (K-means, black dots; Louvain, black stars).

2. To strengthen the discussion of the CellNiche's scalability, it would be valuable to provide additional details regarding the factors that contribute to its efficiency. Further clarification is needed on whether the observed speedup is due to the loss function, the contrastive learning strategy, architecture, or the batch sampling procedure. Audience may be interested in knowing which term make it so fast. Furthermore, more details are needed for memory consumption in the scalability analysis, specify the number of A6000 GPUs employed during training.

Response:

We thank the reviewer for this important question and we fully agree that clarifying the sources of CellNiche's scalability and efficiency will help readers better interpret the runtime results. Below we summarize the main design choices and their contribution to the observed speedup and provide the additional clarifications newly added to the revision.

1 Main factors contributing to efficiency

CellNiche's efficiency is not due to a single component, but rather to the combination of its training objective, model architecture, and microenvironment-based batch sampling strategy:

1.1 Training objective without reconstruction of molecular profiles

As discussed in our response to Comment 1, CellNiche treats molecular identity as an input space rather than a learning target. Consequently, the loss function operates on cell–cell relational structure instead of reconstructing high-dimensional gene expression vectors. This avoids the gene-level reconstruction step used in many encoder–decoder frameworks and allows CellNiche to obtain informative microenvironment representations with comparatively fewer training epochs.

1.2 Lightweight graph encoder architecture

CellNiche employs a relatively shallow graph encoder (based on GraphSAGE-style message passing), rather than deep attention-based architectures. This design keeps both the per-epoch computational cost and the parameter count low, which directly contributes to the reduced runtime in our benchmarks.

1.3 Microenvironment-level batch sampling

During training, CellNiche processes batches of local micro-environment subgraphs rather than complete organizational graphs. This strategy can handle graph-scale datasets and fully utilize the acceleration provided by multi-batch parallel computing.

2 Clarifications on memory usage and GPU configuration

To further quantify resource usage, we measured peak CPU and GPU memory during training on the *"sagittal3"* STARmap slice of the mouse brain, which contains 196,416 cells and thus

provides a representative mid–large–scale setting. CellNiche required a peak CPU resident memory (RSS) of 6.73 GB and a peak GPU reserved memory of 4.47 GB during training.

All GPU-based experiments reported in the manuscript were performed using a single NVIDIA A6000 GPU, and no multi-GPU parallelization was used.

Supplementary Fig. 10 (Additions):

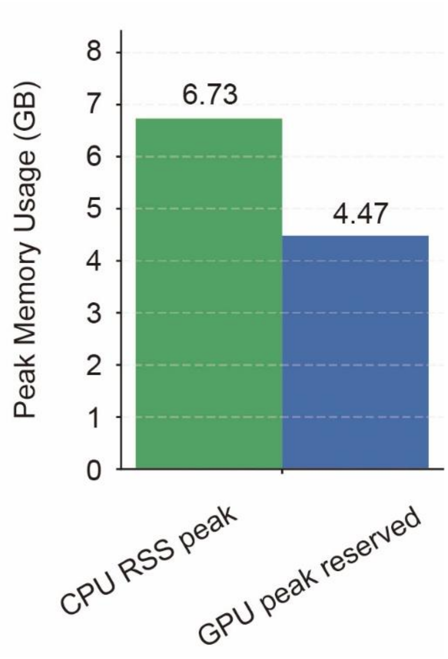

Supplementary Fig. 10: Peak memory usage during CellNiche training on a STARmap PLUS dataset.

Peak CPU resident memory (RSS) and peak GPU reserved memory recorded during training on the "sagittal3" mouse brain slice (196,416 cells). Measurements were obtained under a single NVIDIA A6000 GPU configuration.

3. In reference to analyzing the efficiency/scalability of CellNiche, I guess CellNiche_pheno model is used in the scalability section. If it is the case, it may not be appropriate when compared to other methods since CellNiche_pheno takes cell labels as inputs and other methods do not.

Response:

We thank the reviewer for this insightful comment. In the scalability experiments, reviewer is correct that CellNiche takes cell labels as input and this seems to be unfair compared to other methods which do not use. However as shown in *Supplementary Fig. 6,* we compared the cell type labels input as other molecular identity representation in CellNiche, (e.g., expression, PCA, scVI used by other methods), The CellNiche_pheno's performance is quite close to others thus our comparison results still hold. In addition, we followed the original recommended settings of each method to ensure that the comparison reflects realistic usage scenarios, rather than an artificial "matched-input" setup that may not be representative of how these tools are typically applied.

For CellNiche specifically, using cell labels (CellNiche_pheno) versus expression-based features mainly affects the dimensionality of the input representation, rather than the overall algorithmic complexity. In our runtime experiments (**Supplementary Fig. 9**), the one-hot–encoded cell labels have a dimensionality of roughly ~1,000 (depending on the number of cell types per slice), whereas the expression-based input has 1,022 features. These dimensions are of the same order of magnitude, so the impact on computational cost is minimal. Thus, the observed scalability and efficiency of CellNiche are not driven by a special advantage of using phenotype inputs, but are instead attributable to the underlying model design and training strategy.

4. The use of cell labels for CellNiche_pheno in benchmarking accuracy may not be appropriate, as it could artificially inflate accuracy and does not fully reflect the model's performance compared to other methods which do not use methods. Also, a clear description of what "cell phenotype information" is and how is it obtain is required.

Response:

We thank the reviewer for raising this point. We agree that the use of cell labels for CellNiche_pheno in benchmarking accuracy may artificially inflate accuracy. We did extra experiments shown in *Supplementary Fig. 6,* and we found that using the cell type labels input as other molecular identity representation (e.g., expression, PCA, scVI used by other methods) has very similar performance. CellNiche_pheno didn't artificially inflate accuracy and our results largely reflect the model's performance compared to other methods which do not use

methods. The detailed explanations are as follows.

1 Default input setting follows each method's original design

Throughout the manuscript, unless otherwise specified, CellNiche was run using cell-type labels as its input representation.

For the baseline methods, we followed each tool's native design with respect to input features. Cytocommunity is originally designed to operate on cell-type labels, and therefore used the same input representation as CellNiche in the benchmarking experiments. In contrast, other baseline methods were explicitly developed to process raw gene expression matrices, and we adhered to their published implementations without altering their input formats.

This approach ensures that all methods are evaluated under their intended usage conditions, rather than under imposed or modified input settings.

2 Using cell-type labels does not introduce supervision or task-specific advantage

Although cell-type labels are used as input features, CellNiche does not use labels as training targets. The model remains fully self-supervised, and the objective does not rely on ground-truth annotations. Labels serve only as a molecular identity representation, analogous to other fixed embeddings (e.g., PCA, scVI), rather than as supervisory information.

3 CellNiche is compatible with diverse molecular representations

As shown in **Supplementary Fig. 6** (provided in response to Comment 1), CellNiche maintains stable performance when provided with widely different forms of cellular identity features, including raw expression profiles, PCA embeddings, scVI embeddings, and cell-type labels. In contrast, baseline methods exhibited varying degrees of performance fluctuation under different input representations.

These results indicate that CellNiche's behavior is not tied to a specific molecular feature space, but remains effective across heterogeneous identity formats. This reflects a key design principle of the framework rather than reliance on cell-type labels.

4 Rationale for highlighting the cell-type input in the manuscript

We emphasize the cell-type representation in the manuscript because we observed that using cell-type labels as the molecular identity feature not only achieved comparatively strong performance in cellular microenvironment characterization but also provided a more practical and flexible foundation for downstream cross-slice and cross-atlas integration tasks.

Regarding the definition of "cell phenotypic information," we agree that clarification is necessary. For the benchmark dataset and NSCLC dataset used in the manuscript, the cell type

information we used comes from the original research, and the source of each dataset can be found in the **Data Availability section**. For cross-atlas mouse brain integration data, cell types are uniformly annotated, detailed descriptions can be found in the **Integration and processing of cross-atlas mouse brain data section** of **Methods**.

5. The osmFish spatial transcriptomics dataset used has only 33 genes for performance evaluation. To more rigorously assess the generalizability and robustness of the proposed method, it would be important to evaluate its performance on larger-scale spatial transcriptomics datasets with higher gene coverage.

Response:

We appreciate the reviewer's suggestion regarding the need to assess performance on datasets with higher gene coverage.

First, we selected the osmFISH dataset not merely based on gene count, but because it profiles the mouse somatosensory cortex, a tissue with a well-characterized laminar organization. This dataset provides a biologically meaningful benchmark for evaluating whether a method can recover canonical microenvironmental organization, even when gene coverage is limited. In this context, osmFISH serves as a valuable test of a model's ability to extract spatial structure under constrained molecular information.

Second, in the revised manuscript we have added performance evaluations on a spatial transcriptomics dataset generated using the Stereo-seq platform (mouse embryo dataset; **Supplementary Fig. 7**). This dataset is generated using a whole-transcriptome profiling protocol and therefore includes substantially more genes. Across this dataset, methods such as STAGATE and GraphST—which adopt encoder–decoder architectures with expression reconstruction losses to model high-dimensional gene expression profiles—showed competitive performance to CellNiche on AMI, ARI, and Macro-F1. This suggests that reconstruction-based methods that extract latent structure from the original expression profiles are particularly advantageous when the cellular identity feature are high-dimensional and noisy.

Notably, CellNiche achieved the highest Silhouette scores among the compared methods, suggesting that its contrastive, microenvironment-focused design yields more compact and well-separated niche embeddings, even when accuracy-based metrics are comparable. This aligns with CellNiche's aim of modeling spatial microenvironments directly rather than reconstructing expression profiles.

Supplementary Fig. 7 (Additions):

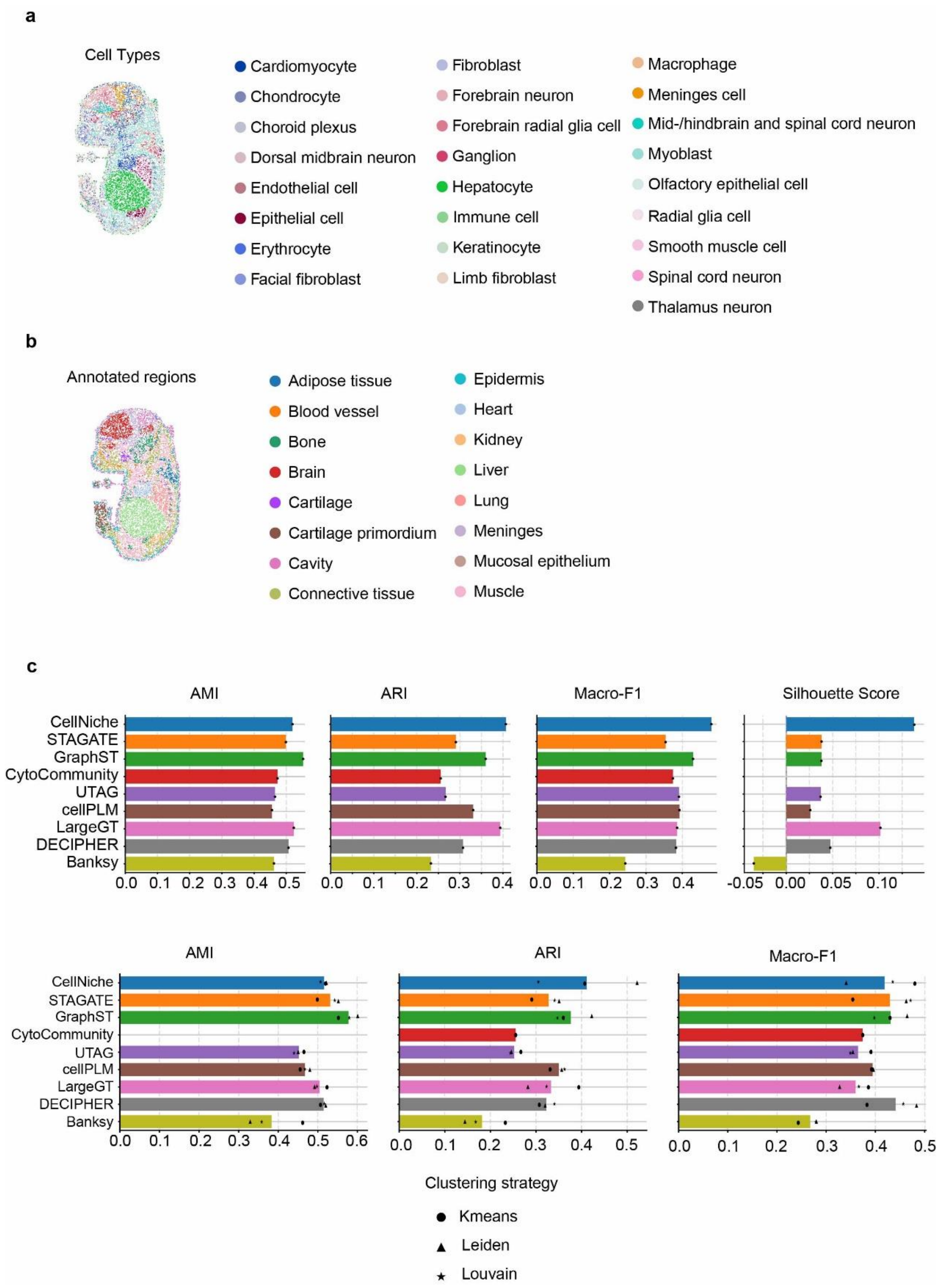

Supplementary Fig. 7: Benchmark performance under different clustering strategies on the Stereo-seq mouse embryo dataset (slice "E16.5_E2S4").

a, Spatial map colored by annotated cell types. b, Spatial map colored by annotated tissue regions. c, Benchmark comparison across methods using AMI, ARI, Macro-F1, and Silhouette score. Top row: scores obtained using K-means clustering, consistent with the main text settings.

Bottom row: mean scores under three clustering strategies, and marker symbols indicate the scores obtained under each clustering strategy (circle: K-means; triangle: Leiden; star: Louvain).

6. Relying solely on k-means–based evaluation may be problematic, as it is not a direct evaluation of the embedding quality and it also depend on the clustering. A more comprehensive assessment could be achieved by comparing the embeddings directly against the ground-truth cell labels using metrics such as the silhouette index.

Response:

We appreciate the reviewer's suggestion. We agree that relying solely on K-means–based evaluation may not fully capture embedding quality.

First, in the revised manuscript we have added the Silhouette score as an additional evaluation metric for all benchmarking datasets (**Fig 2e, 2j, Fig 3e, 3j**). Unlike clustering-dependent accuracy metrics, the Silhouette score directly quantifies the compactness and separation of the model's output embeddings with respect to ground-truth labels and is independent of the choice of clustering algorithm.

Second, to reduce dependence on a single clustering algorithm, we further evaluated each method under three clustering strategies—K-means, Leiden, and Louvain. For the four benchmark datasets presented in the manuscript, we selected one representative slice from each (osmFISH: full dataset; CODEX: BALB/c-1; STARmap: well11; CODEX: reg007_A) and compared performance across all methods under the three clustering strategies (**Supplementary Fig 2, 3, 4, 5**).

Supplementary Fig. 2-5 (Additions):

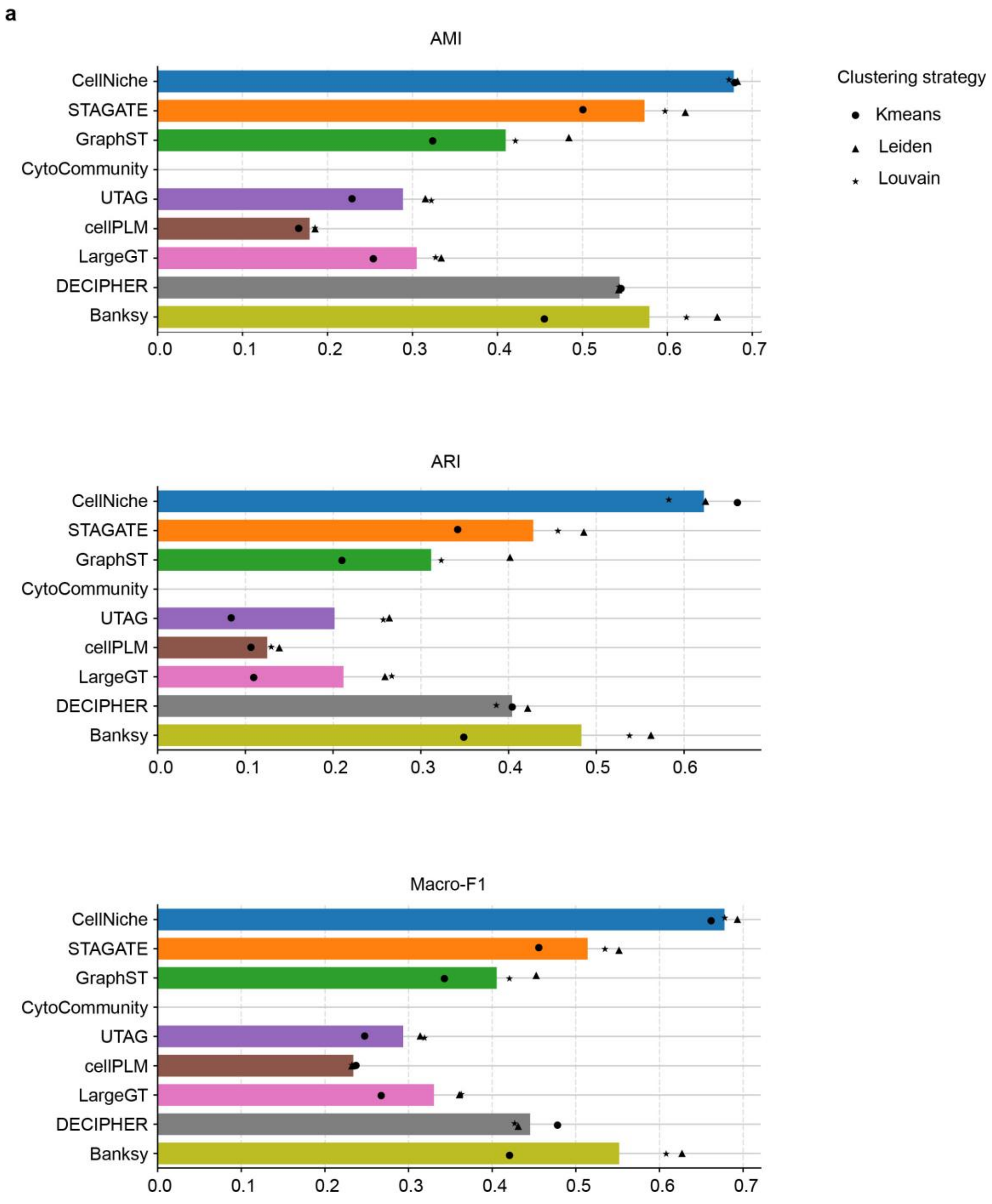

Supplementary Fig. 2: Benchmark performance under different clustering strategies on the mouse somatosensory cortex osmFISH dataset.

Bar plots show the average agreement with the reference region annotations across three clustering strategies (K-means, Leiden, and Louvain) for each method. Marker symbols indicate the score obtained under each clustering strategy (circle: K-means; triangle: Leiden; star: Louvain). Metrics include AMI, ARI, and Macro-F1.

a

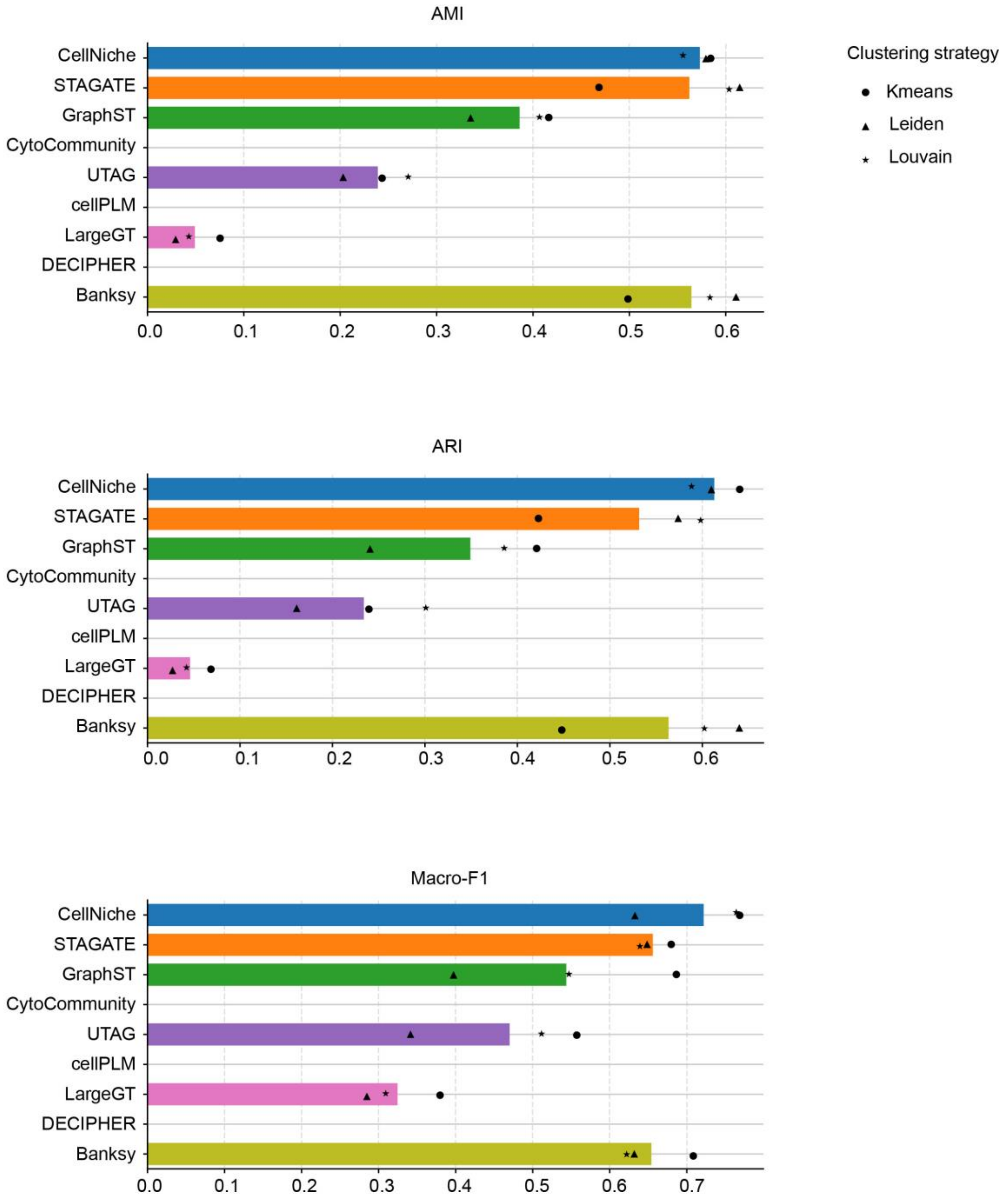

Supplementary Fig. 3: Benchmark performance under different clustering strategies on the mouse spleen CODEX dataset (slice "BALB/c-1").

Bar plots show the average agreement with the reference region annotations across three clustering strategies (K-means, Leiden, and Louvain) for each method. Marker symbols indicate the score obtained under each clustering strategy (circle: K-means; triangle: Leiden; star: Louvain). Metrics include AMI, ARI, and Macro-F1.

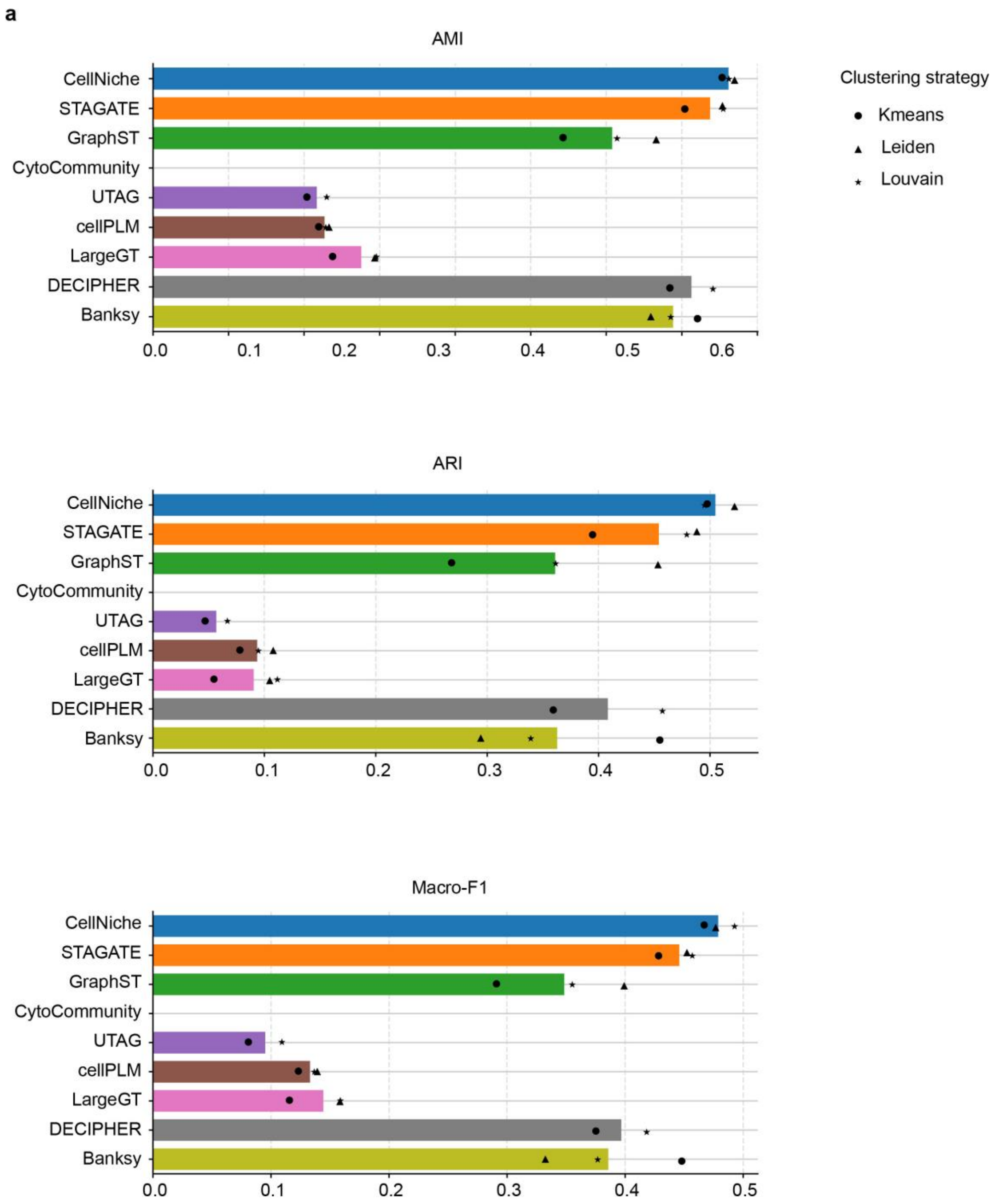

Supplementary Fig. 4: Benchmark performance under different clustering strategies on the STARmap mouse brain dataset (coronal slice "well11").

Bar plots show the average agreement with the reference region annotations across three clustering strategies (K-means, Leiden, and Louvain) for each method. Marker symbols indicate the score obtained under each clustering strategy (circle: K-means; triangle: Leiden; star: Louvain). Metrics include AMI, ARI, and Macro-F1.

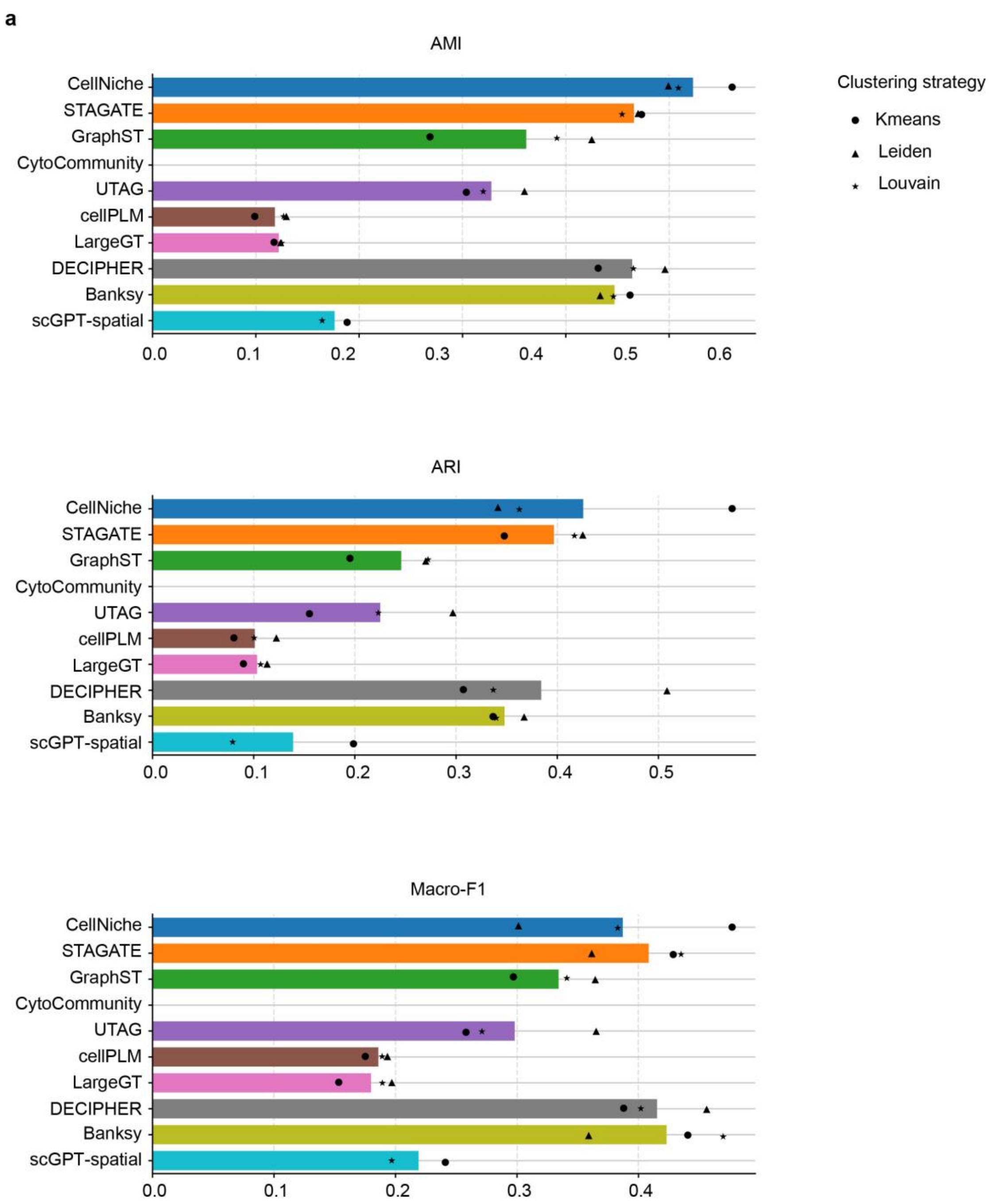

Supplementary Fig. 5: Benchmark performance under different clustering strategies on the human colorectal cancer CODEX dataset (slice "reg007_A").

Bar plots show the average agreement with the reference region annotations across three clustering strategies (K-means, Leiden, and Louvain) for each method. Marker symbols indicate the score obtained under each clustering strategy (circle: K-means; triangle: Leiden; star: Louvain). Metrics include AMI, ARI, and Macro-F1.

7. A clear description of the model's output and the procedure by which spatial microenvironments (cell niches) are identified is needed, particularly in the overview of the Results section.

Response:

We appreciate the reviewer's request for greater clarity regarding the model output and the procedure for identifying spatial microenvironments.

First, CellNiche produces a low-dimensional embedding vector for each individual cell. This embedding reflects the cell's spatial microenvironmental context as learned through the contrastive objective, and constitutes the direct output of the model. No cluster labels or predefined niche categories are generated during training.

Second, spatial microenvironments (cell niches) are identified post hoc by applying a clustering algorithm (e.g., K-means, Leiden, or Louvain) to these cell-level embeddings. The resulting clusters represent groups of cells sharing similar microenvironmental signatures and are treated as discrete niche assignments for downstream analyses.

To improve clarity, we have now added this point in **Results section 1**.

Results section 1 (Additions) (Line 170):

"

After training, CellNiche generates a low-dimensional embedding vector for each cell. This embedding vector reflects the cellular microenvironment learned through contrastive targets and is a direct output of the model. Specific cellular microenvironments can be identified by clustering these cell vectors.

"

8. The section on NSCLC where cell-cell communication is used is not explained clearly, and the analysis of tertiary lymphoid structures (TLSs) on C0 requires further elaboration. It is not clear what is the consequence of two niche was predominantly present in two samples. For C4 analysis, it is unclear why only two cell type labels (B cell and Cd4) are emphasized and how this choice reflects biological realism. Moreover, the description does not fully align with the corresponding figure (Fig. 4e), which creates confusion.

Response:

We thank the reviewer for pointing out that our description of the TLS-related communication analysis in NSCLC needed clearer motivation and tighter alignment with the figures. In the revised manuscript, we (i) clarify that the purpose of highlighting niche C0 is to demonstrate a

conserved, lymphoid-enriched microenvironment consistent with TLS-like regions; (ii) explicitly motivate our focus on the antigen-presentation axis (MHC-I) as a contact-dependent interaction mode relevant to TLS biology; (iii) correct the niche identifier and revise the text to match Fig. 4d–e; and (iv) explain that the emphasized cell types are those showing the strongest spatial proximity to CD8 naïve T cells within C0, thereby illustrating how spatial niche structure can contextualize expression-based communication inference.

We have now added this point to **Results section 7** and **Fig. 4** of our revised manuscript.

Results section 7 (Additions) (Line 276):

"

In contrast, several immune–stromal niches exhibited conserved patterns across samples, suggesting shared immune programs recurring across independent patients. A prominent example was niche C0, which was strongly enriched for lymphoid-lineage cells, including B cells, T CD4 memory, and T CD8 memory[38]*, and was predominantly present in LUAD-5 and LUAD-12 (**Fig. 4c**). This composition and spatial pattern were consistent with tertiary lymphoid structures (TLSs) annotated in the original study (**Supplementary Fig. 12b**). TLSs are ectopic, organized lymphoid aggregates that arise in chronically inflamed tissues and tumors, often featuring compartmentalized B- and T-cell zones and serving as local sites for antigen presentation and adaptive immune activation. To illustrate how spatial niche structure can contextualize expression-based communication inference, we focused on antigen presentation (MHC-I)*[39,40]*—a hallmark TLS function and an interaction mode that is inherently contact-dependent*[41]*. CellChat*[42] *inferred a broad set of transcriptionally putative MHC-I interactions involving CD8 naïve T cells and multiple additional cell types within C0 (**Fig. 4d**). By contrast, spatial co-localization analysis within the same niche showed that the strongest proximity signals for CD8 naïve T cells were concentrated in B cells and CD4 naïve T cells, among the cell types present in C0 (**Fig. 4e**). Given that effective MHC-I–mediated antigen presentation requires close cell–cell proximity*[41]*, these results suggest that incorporating spatial niche structure can help refine interaction hypotheses derived from expression-only inference and yield interaction patterns that better reflect local tissue organization.*

"

Fig. 4 (Additions):

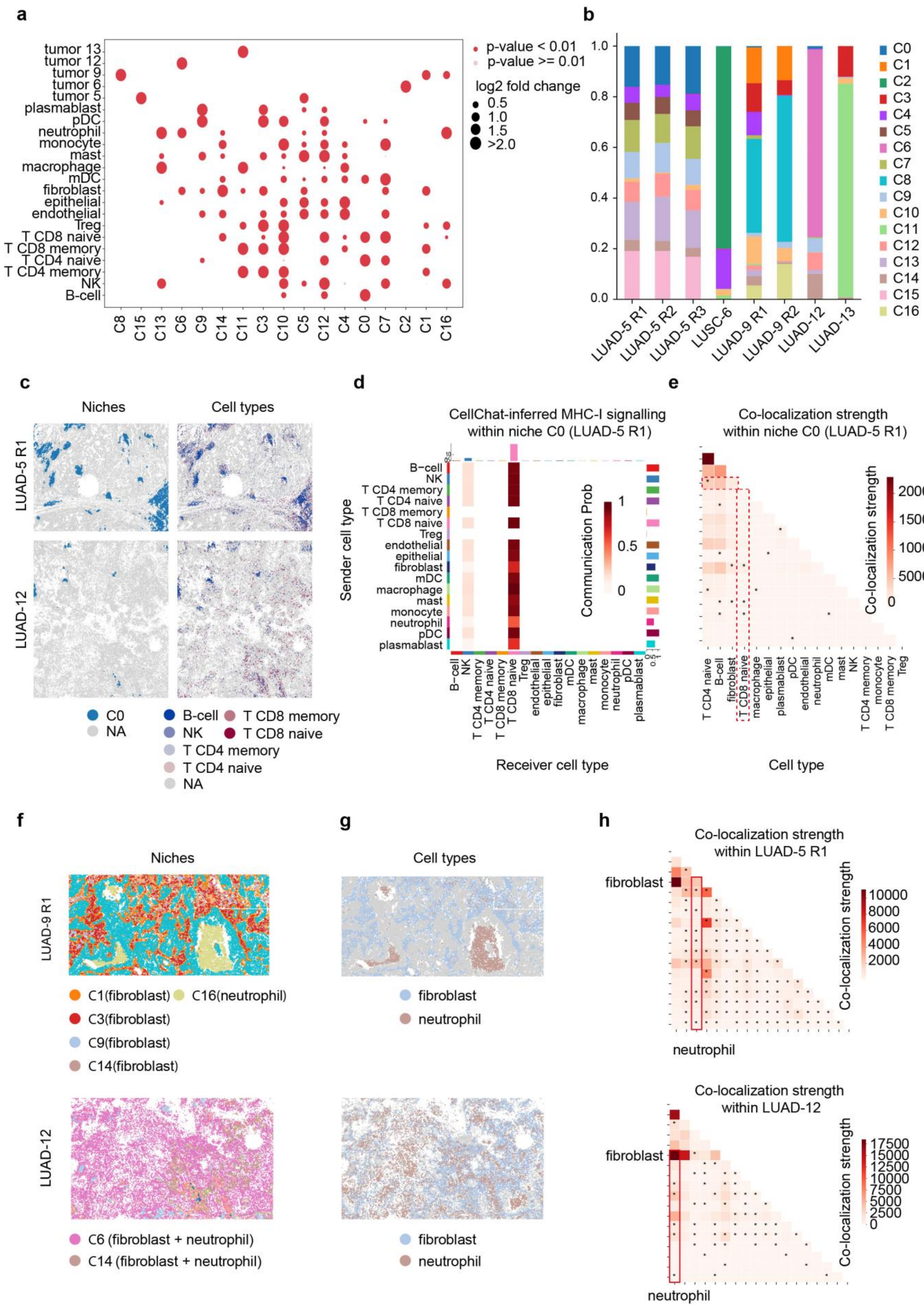
a
b
c
d
e
f
g
h
p-value < 0.01
p-value >= 0.01
log2 fold change
Niches
Cell types
LUAD-5 R1
LUAD-12
LUAD-9 R1
CellChat-inferred MHC-I signalling within niche C0 (LUAD-5 R1)
Co-localization strength within niche C0 (LUAD-5 R1)
Sender cell type
Receiver cell type
Communication Prob
Co-localization strength
Cell type
C1(fibroblast)
C16(neutrophil)
C3(fibroblast)
C9(fibroblast)
C14(fibroblast)
C6 (fibroblast + neutrophil)
C14 (fibroblast + neutrophil)
fibroblast
neutrophil
Co-localization strength within LUAD-5 R1
Co-localization strength within LUAD-12

*Fig. 4 CellNiche deciphers cellular microenvironments in NSCLC. a, Cell-type enrichment across the 17 CellNiche microenvironments (niches). Dot size denotes log2 fold change and color indicates significance ($P < 0.01$). b, CellNiche's spatial niche proportions in each sample. c, Spatial maps highlighting the lymphoid-enriched niche C0 (left) and the corresponding lymphoid cell types (right) in LUAD-5 R1 (top) and LUAD-12 (bottom). d, CellChat-inferred MHC-I signaling communication probabilities among cell types within niche C0 in LUAD-5 R1. e, Proximal cell-cell interactions between different cell types within C0. Color bars indicate the interaction strength between cell type i and cell type j. Permutation test (n = 1,000): * $p < 0.05$. f, g, h, Spatial distribution of fibroblast-enriched and neutrophil-enriched niches (f), spatial distribution of fibroblast and neutrophil (g), and cell-cell interaction strengths within slices (h), visualized for LUAD-9 R1 (top) and LUAD-12 (bottom).*

9. In the final results section, further details are needed to explain why additional Leiden clustering was performed and how it supports the claim of subregion refinement of slice Zhuang-ABCA-1.069 (Atlas 3). The rationale for applying this extra clustering step should be clearly articulated. Additionally, what is referred to as Figure 8 appears to correspond to Figure 7b.

Response:

We thank the reviewer for highlighting the need to clarify the purpose of the additional Leiden clustering and its relation to the proposed subregion refinement. In the revised manuscript, we now explicitly state that Leiden clustering is performed on the CellNiche learned embeddings as an independent, unsupervised test of whether the refinement signal is robust in the continuous representation space, rather than being dependent on a particular discretization or a transferred label assignment.

Specifically, for the region refinement example (FbTrt), we show that the cells reassigned to FbTrt consistently form a spatially coherent and contiguous domain across a wide range of Leiden resolutions (e.g., resolution 0.1 – 2; 11 – 57 clusters), demonstrating that this refinement is stable in embedding space and not sensitive to the clustering granularity (**Supplementary Fig. 16a**).

For the subregion refinement example (CNU_13), we clarify that the bipartition is first supported within Atlas 1 well03 by embedding separation and marker evidence, and then we use Leiden clustering across multiple resolutions (e.g., resolution 2→0.1; 41→6 clusters) to show that the two CNU_13 subdomains remain separable even under coarse clustering, indicating that the subdivision reflects a stable feature of the learned embedding geometry (**Supplementary Fig. 16b**).

We have now added this point in **Results section 8** and **Supplementary Fig. 16** of our revised manuscript.

Results section 8 (Additions) (Line 413):

"

*Importantly, unsupervised Leiden clustering on the CellNiche embeddings showed that the putative FbTrt cells consistently formed a spatially coherent domain across a wide range of clustering resolutions, indicating that this refinement is robust in the learned representation space (**Supplementary Fig. 16a**).*

"

Results section 8 (Additions) (Line 421):

“

*To assess whether this subdivision reflects a stable feature of the learned representation—rather than an artefact of a particular clustering resolution or discretization—we performed Leiden clustering on the CellNiche embeddings and evaluated the partition across multiple resolutions. Notably, the two CNU_13 subdomains remained separable even under coarse clustering (i.e., a small number of global clusters) and persisted across decreasing resolutions, indicating that the partition is supported by the continuous embedding geometry rather than a single discretization (**Supplementary Fig. 16b**).*

”

Supplementary Fig. 16 (Additions):

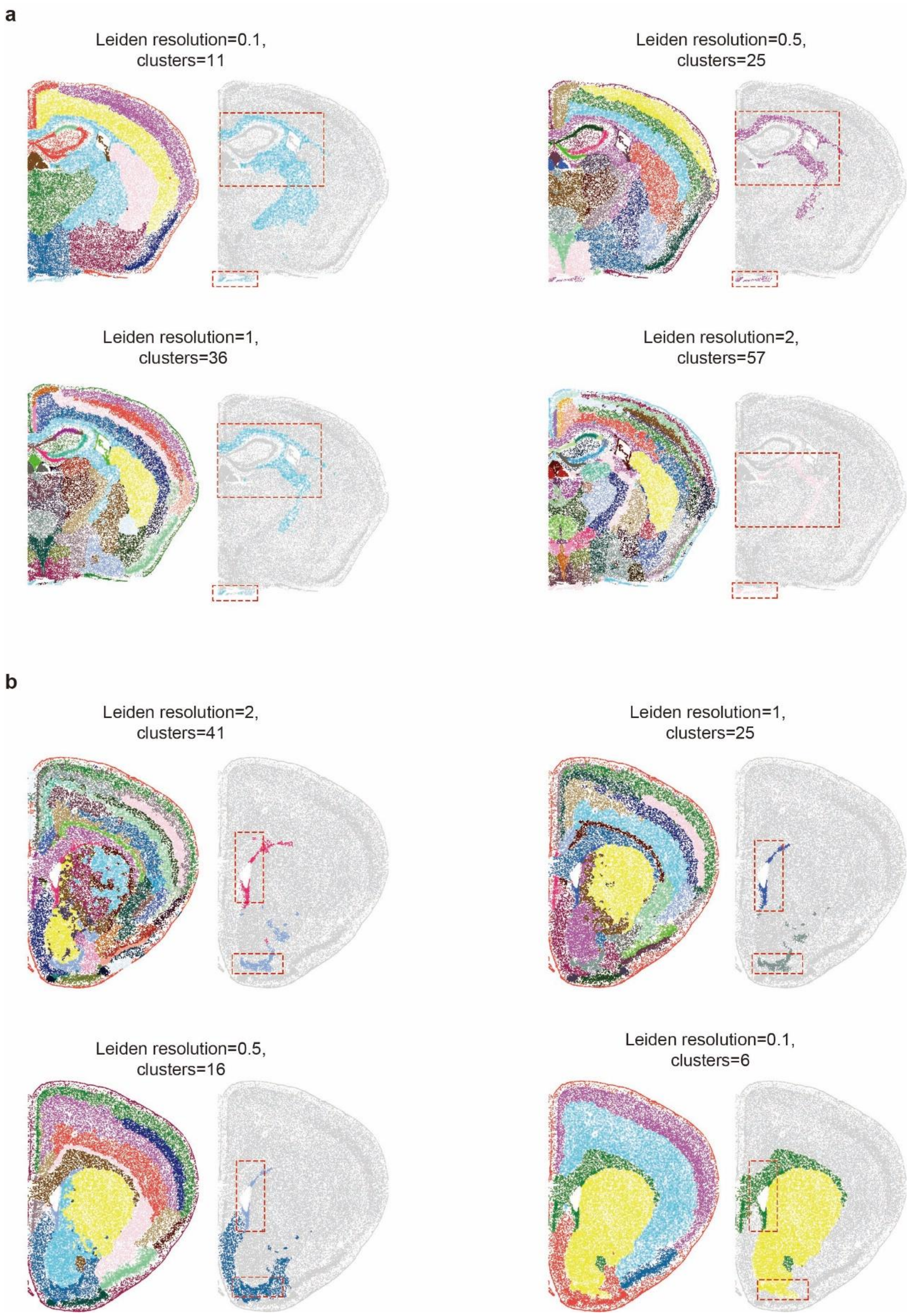

Supplementary Fig. 16: Robustness of the FbTrt refinement and the CNU_13 bipartition across Leiden resolutions.

a, Leiden clustering was performed on CellNiche embeddings for the Atlas 1 well05 section, using a range of resolution parameters (as indicated). Left panels show the full Leiden partition;

right panels highlight the cells assigned to FbTrt by cross-atlas label transfer. Across resolutions (from coarse to fine partitions), FbTrt-labeled cells consistently form a coherent and spatially contiguous domain. b, Leiden clustering on CellNiche embeddings was evaluated across multiple resolutions for the Atlas 1 well03 section to test the stability of the proposed CNU_13 subdivision. Even under coarse clustering (few global clusters), the two CNU_13 subdomains remain separable in the embedding-derived partitions.

Minor Comments:

1. In Methods section, additional details are needed regarding how Gene Set Variation Analysis (GSVA) and Differential Gene Expression (DGE) were performed.

Response:

We thank the reviewer for pointing out the need for clearer methodological details regarding the Gene Set Variation Analysis (GSVA) and differential gene expression (DGE) analyses. We have now expanded the corresponding descriptions in the revised **Methods section**.

Methods section (Additions) (Line 826):

"

Gene Set Variation Analysis (GSVA)

To quantify pathway-level activity across the cellular microenvironments identified by CellNiche, we performed GSVA on pseudo-bulk expression profiles. For each niche, log-normalized gene expression values were averaged across all cells to obtain a cluster-by-gene matrix. GSVA was performed using the gseapy (v.1.1.11) implementation of the original GSVA method, with the MSigDB Hallmark gene set collection (h.all.v2024.1). Enrichment scores were computed using the default Gaussian kernel (kcdf="Gaussian"). The resulting GSVA matrix (pathway × niche) was used to evaluate biological programs associated with immune activation, stromal remodeling, and metabolic transitions.

"

Methods section (Additions) (Line 835):

"

Differential Gene Expression Analysis

Differentially expressed genes for each CellNiche microenvironment were identified using the Wilcoxon rank-sum test implemented in scanpy.tl.rank_genes_groups. Each microenvironment was compared against all remaining cells ("rest"), and P-values were adjusted using the Benjamini–Hochberg false discovery rate (FDR). Log-normalized expression matrices were used as input. For each microenvironment, the top-ranked genes were selected for visualization and downstream interpretation.

"

2. The implementation details for Harmony and FuseMap should be added to the Methods.

Response:

We have now added this point in **Methods section.**

Methods section (Additions) (Line 843):

"

Batch Correction with Harmony

Batch correction across samples was performed using Harmony via the sce.pp.harmony_integrate function. Log-normalized expression values were first reduced using principal component analysis (sc.pp.pca). Harmony integration was then applied with the sample identifier specified as the batch covariate (sce.pp.harmony_integrate(adata, key='sample')), using default parameters. The corrected PCA embeddings were used for downstream analysis.

"

Methods section (Additions) (Line 850):

"

Integration with FuseMap

FuseMap was applied following the official implementation and default settings provided by the authors. No hyperparameters or preprocessing procedures were modified. The method was used as released, ensuring consistency with the original design of FuseMap.

"

3. Information on cluster stability assessment should be provided rather than solely citing previous work.

Response:

We have now added this point in **Methods section.**

Methods section (Additions) (Line 818):

"

Determination of the Number of Cellular Microenvironments in the NSCLC Dataset

The number of cellular microenvironments was determined using a clustering stability procedure proposed by Varrone et al. This method evaluates the robustness of clustering results at adjacent K values. Briefly, Gaussian mixture models (GMMs) are fitted at K-1, K, and K+1, respectively, and the similarity between microenvironments is quantified using the Fowlkes-Mallows index (FMI). K values that consistently maintain a high FMI value across multiple runs are determined as stable K values. For the selected K value, the GMM model with the highest marginal likelihood is retained.

”

4. Figure 4.d,e, and h need proper x-axis and y-axis labeling. Additionally, the description of Figure 4h in the figure legend should be clarified.

Response:

We appreciate the reviewer's careful examination of the figures. In the revised manuscript, we have updated the axis labels for Fig. 4d, 4e, and 4h and refined the legend of Fig. 4h to ensure that the contents and interpretations of these panels are clearly stated **(Fig. 4)**.

5. GraphSAGE should be cited in the training module section of Methods.

Response:

Thank you for pointing this out. We have now added the appropriate citation to GraphSAGE in the training module section of the **Methods** in the revised manuscript.

6. Figure 7e appears to be missing.

Response:

We apologize for this oversight. In the revised version, we have corrected the figure citation and removed the incorrect reference to "Fig. 7e".

Results section (Additions) (Line402):

“

*Similarly, we successfully transferred 107 subregion annotations with 91.3% accuracy (**Supplementary Fig. 15a, b**).*

”

7. Figure S3 may correspond to Figure S6; this should be clarified.

Response:

We thank the reviewer for catching this inconsistency. We have carefully checked all supplementary figure references and clarified the correspondence in the **Results section**.

Results section (Additions) (Line356):

“

*To balance resolution and performance, we chose the "supertype" level as the cell phenotype representation (**Supplementary Fig. 8**).*

”

8. A clearer definition of tertiary lymphoid structures (TLSs) is needed, along with an explanation of their relevance to this analysis.

Response:

This concern is addressed in detail in our response to Major Comment #8 (see above). Briefly, in the revised manuscript we have added a clear definition of TLSs in the Results section, together with an explanation of their immunological relevance and why they serve as a biologically meaningful context for the analysis of niche C0.

9. Figure 3d is not referenced and the information on evaluation of the STARmap dataset using ARI, NMI, Macro-F1, and Micro-F1 metrics is missing.

Response:

We thank the reviewer for highlighting this omission. In the revised manuscript, we now explicitly reference Fig. 3d in the **Results section** and clarify that it reports the quantitative evaluation on the STARmap dataset.

Results section (Additions) (Line 239):

"

*In contrast, other methods produced clusters with substantial regional mixing, failing to delineate the fine spatial organization of brain substructures (**Fig. 3d**).*

"

10. The introduction should include the application of the NSCLC dataset and describe how the biological validation is planned.

Response:

We thank the reviewer for this helpful suggestion. In the revised manuscript, we keep the Introduction primarily focused on positioning CellNiche within existing work and explaining the conceptual and methodological design, which is standard for a methods-oriented study. The application to the NSCLC cohort and the associated biological validation are now: (i) briefly highlighted in the Abstract, and (ii) described in detail in the dedicated NSCLC Results subsection and corresponding Methods section.

11. The term "walk" used in the methodology should be clearly explained for clarity and reproducibility.

Response:

We agree that the term "walk" required a clearer definition for reproducibility. In the revised manuscript, we have now clarified this point in **Methods section.**

Methods section (Additions) (Line 625):

"

A walk is a Markovian node sequence $(v_0, v_1, \ldots, v_L)$ generated by repeatedly sampling the next node from the current node's neighbors according to a transition distribution $P(v_{t+1} \mid v_t)$. In the default setting, we use a uniform transition over neighbors, i.e. $P(j \mid u) = 1/\mid \mathcal{N}(u) \mid$ for $j \in \mathcal{N}(u)$, where $\mathcal{N}(u)$ is the neighbor set of node u.

"

Reviewer #2 (Remarks to the Author):

The authors propose a neural-network approach based on contrast learning to identify and characterize cellular microenvironments from spatial omics data. The authors show their approach performs better and faster compared to current approaches for numerous datasets. A few comments:

1. Although it's infeasible to compare with every single other method, the authors should try to compare with the most popular recent methods, and those identified to be high-performing in benchmark papers. E.g., BANKSY (https://www.nature.com/articles/s41588-024-01664-3) and top methods in https://link.springer.com/article/10.1186/s13059-024-03361-0 – "Benchmarking clustering, alignment, and integration methods for spatial transcriptomics". The last link also details methods which have good scalability, including BANKSY, Dr.SC, SpatialPCA, Spaceflow and PRECAST. Don't have to implement all, but perhaps the ones which have both good scalability and high performing, e.g., BANKSY and SpatialPCA. It would also be good if the authors use the same datasets in the BANKSY/Spatial PCA and comparison papers, for fair comparison.
A similar comment applies to the integration methods chosen for Figure 6 – the authors only chose Harmony and FuseMap. It will be good if the authors either justified their choice (e.g., is there a recent review paper which showed FuseMap or Harmony was a top performing method?) or consider other recent methods, e.g., STaligner (https://www.nature.com/articles/s43588-023-00528-w), SPIRAL (https://link.springer.com/article/10.1186/s13059-023-03078-6), graphST, etc..

Response:

We thank the reviewer for the thoughtful suggestions. In the revised manuscript, we expanded both our benchmarking analyses and our evaluation of integration tools in direct response to comments from all reviewers.

1 Expanded benchmarking with additional high-performing methods

Incorporating feedback from all reviewers, we added three representative spatial omics methods

to our benchmarking comparisons: DECIPHER, Banksy and scGPT-spatial. DECIPHER and BANKSY are tools introduced in recent years, demonstrating strong scalability and excellent performance in large-scale spatial transcriptomics benchmarks. scGPT-spatial represents a spatial omics approach based on a fundamental model.

2 Evaluation of additional integration methods in the cross-atlas mouse brain task

To further strengthen the cross-atlas integration analysis, we considered three state-of-the-art integration frameworks: STaligner, SPIRAL, and GraphST.

STaligner was able to run on this task, but consistently failed to learn meaningful embeddings This behavior is consistent with the examples shown in the STaligner publication, where the datasets used all contained large overlapping gene sets across slices, such as: "4 slices from human dorsolateral prefrontal cortex (DLPFC) (47,681 cells, 800 genes)", "mouse olfactory bulb (Stereo-seq + Slide-seqV2) (39,248 cells, 1,506 genes)", "4 mouse embryo slices profiled by Stereo-seq (E9.5–E12.5) (95,896 cells, 693 genes)", "mouse hippocampus slices from Slide-seqV1 (171,645 cells, 1,032 genes)" In our setup, the four mouse atlases shared only 52 intersecting genes, which may have prevented them from fully learning meaningful information.

Regarding the SPIRAL algorithm, training on a GPU for the same cross-map dataset (approximately 270,000 cells) is expected to take 80 hours. Given that this is only a relatively small subset of the complete map (our complete map analysis involves over 10 million cells), the SPIRAL algorithm does not meet the scalability requirements of our research and was therefore not trained.

We note that GraphST had already been evaluated in our initial benchmarking tests. In those experiments, the method failed to run on datasets exceeding ~80,000 cells, indicating that it is not suitable for the large-scale datasets considered in our study.

2. The authors indicate that batch effects is mitigated because the input are cell type labels, which makes sense and is fine. However, it's not clear whether there will be batch effects when the input is expression profiles, which is discussed in the loss function module.

Response:

We thank the reviewer for this important point. The question raised here is closely aligned with comment #1 of reviewer #1 regarding the conceptual motivation behind decoupling molecular identity representations from spatial modeling. We summarize the key clarification below and refer the reviewer to our detailed response to Reviewer #1 (Major Comment 1).

In CellNiche, molecular identity is treated as a fixed input space rather than a feature that is re-learned or transformed within the model. As a result, CellNiche does not couple expression-

level feature learning with spatial modeling. This design has two implications for batch effects:

1. Batch handling for expression-based inputs can be performed entirely upstream.

Because CellNiche does not perform encoder–decoder–style representation learning, it does not propagate or amplify batch variation present in raw expression profiles. Instead, users may flexibly apply any standardized batch-correction strategy—such as scVI-derived latent embeddings, Harmony-corrected PCA, unified cell-type annotations, or future foundation model based reference embeddings—before training. CellNiche directly ingests these corrected representations without requiring model-level modifications.

2. When cell-type labels are used as input, batch effects are naturally mitigated.

Cell-type annotations define a unified identity space across samples or platforms. In our manuscript, this design enabled: (i) joint modeling of a multi-sample NSCLC cohort, and (ii) cross-platform integration of mouse brain atlases profiled by different technologies. In both cases, CellNiche operated effectively because batch variation was resolved at the identity level before spatial modeling.

While these results do not cover every possible batch scenario, they provide empirical support for the flexibility and practicality of the decoupled design in mitigating batch effects.

3. Parts of the text can be made more clear/grammatical mistakes, e.g.,

• The "strategies" in Figure 1a was a bit confusing, as I didn't see how it matched with those in the text. E.g. the text talks about "batch sampling" "constrastive learning" and other uses of the word "strategy".

• Line 70 – can't a cells' function be determined by other omics, e.g., proteins?, not just gene expression

• Line 83 – the connection between hypothesis-drive and data-drive paradigms, and spatial coordinates/structural constraints and neural networks can be made more clear. Similar comment for line 114 – the "hypothesis drive modelling basd on spatial.." wasn't that clear to me. Also first letter of "hypothesis" should be capitalized.

• Page 1, line 37, "microenvironment, Inspired"

• Is Figure 8b a typo? (line 401)

Response:

We thank the reviewer for these detailed suggestions on improving clarity and correcting grammatical issues. We have carefully revised the manuscript to address each point, ensuring better alignment between text and figures, more precise language, and grammatical accuracy. Specifically:

1. To resolve the confusion regarding "strategies" in Figure 1a, we have revised the

corresponding text in the **Results section** ("CellNiche computationally reconstructs cell niche...") to explicitly label and describe the components. This ensures direct matching between the figure and text, avoiding ambiguity in terminology.

Results section (Additions) (Line 146):

"

*Combined with a cell-centric microenvironment batch sampling strategy (**Fig. 1a**, strategy 1)*

"

Results section (Additions) (Line 153):

"

*..., CellNiche can take alternative predefined identity features (e.g., expression-derived features, learned embeddings, or standardized cell-type annotations), allowing batch effects to be handled flexibly upstream without modifying the spatial model (**Fig. 1a**, strategy 2).*

"

Results section (Additions) (Line 158):

"

*..., CellNiche employs a microenvironment-context-based contrastive learning strategy (**Fig. 1a**, strategy 3).*

"

2. We agree that cellular function can involve multiple omics layers beyond gene expression. We have updated the sentence in **Introduction section**.

Introduction section (Additions) (Line 70):

"

The function of a cell is determined by its molecular state (e.g., gene expression or protein profiles) ...

"

3. To clarify the connection in line 83 (now revised), we have rephrased in **Introduction section**. Similarly, for line 114 (now revised), we have capitalized "Hypothesis-driven" and clarified in **Introduction section**.

Introduction section (Additions) (Line 82):

“

These methods combine hypothesis-driven (leveraging spatial coordinates as explicit structural constraints to define graph topology, where the model does not further modify the graph structure derived from physical coordinates) and data-driven (instead of relying on a predefined spatial graph structure based on physical coordinates, the model infers the interaction patterns through data) modelling.

”

Introduction section (Additions) (Line 120):

“

Hypothesis-driven modeling based on spatial adjacency is inherently suited for capturing local microenvironmental organization, providing an inductive prior that guides the learning process.

”

4. The capitalization error on page 1, line 37 (in the Abstract) has been corrected.

   Abstract section (Additions) (Line 36):

   “

   Within each localized microenvironment, inspired by the one-dimensional co-occurrence modeling in language models, …

   ”

5. The reference to Figure 8b on line 401 (in the **Results section**, "CellNiche enables harmonized integration of tissue regions across atlases") was indeed a typographical error; it has been corrected to **Fig. 7b**.

4. It will be useful if the authors give more detail of the different cellNiche variants (perhaps in the Methods), and which scenarios should they be used. E.g., the authors indicate that there are scenarios when CellNiche_expr is the best-performing.

Response:

We appreciate the reviewer’s suggestion. Rather than defining multiple fixed “variants,” CellNiche was intentionally designed around a representation-agnostic modeling principle: molecular identity is treated as a given input space, not a feature to be re-learned, compressed, or reconstructed by the model. As clarified in our response to Reviewer #1 (Major Comment

1), CellNiche does not bind its performance to any specific form of molecular representation. Instead, it can directly operate on diverse identity spaces—including raw expression profiles, PCA/scVI embeddings, protein features, or cell-type annotations—without modifying the model architecture or training objective.

This design contrasts with reconstruction-based encoder–decoder frameworks, which implicitly assume a specific representation type and jointly re-learn both molecular and spatial information. In CellNiche, spatial microenvironment structure—not molecular identity—is the sole learning target. This allows the method to flexibly inherit whatever identity representation is most appropriate for a given dataset.

In the main manuscript, we primarily showcased results using cell-type annotations as the input identity features for two reasons: 1) Cell-type labels provide a denoised and biologically interpretable representation, which often yields stronger and more stable microenvironment delineation than raw expression profiles, particularly in noisy or lower-coverage spatial assays. 2) For integration tasks, such as the NSCLC cohort and the cross-atlas mouse brain analysis, a unified cell-type identity space offers a practical and highly flexible way to mitigate batch effects across samples and technologies.

While we also evaluated alternative input representations in the extended benchmarking experiments (**Supplementary Fig. 6, Supplementary Fig. 8**), discussing the impact of different molecular identity features on microenvironment representation is not the focus of this.

Reviewer #2 (Remarks on code availability):

Haven't installed, but their Github page seems to have detailed instructions for installing and running the application. There are also numerous tutorials.

Response:

Thank you for this summary!

Reviewer #3 (Remarks to the Author):

Summary:
This manuscript presents CellNiche, a scalable contrast learning framework for identifying and characterizing cellular microenvironments from spatial omics data. When applied to over 10 million cells spanning 6 distinct spatial omics platforms, the framework demonstrated superior performance compared to state-of-the-art methods, alongside robust atlas-scale scalability and batch-correction capabilities. While CellNiche holds promise for advancing spatial microenvironment analysis, the manuscript requires improvements in several aspects, including

methodological contextualization, result validation, and content accuracy. Detailed comments are as follows:

Response:

Thanks for the nice summary!

Major comments:

1. The manuscript claims that the tool enables the analysis of large-scale heterogeneous spatial multi-omics data—a functionality already offered by existing tools such as DECIPHER (also published in Nature Communications). Notably, key capabilities highlighted for the proposed tool, including "model large-scale heterogeneous spatial data in silico effectively and efficiently", "a novel cross-scale contrast learning strategy", "enable delineating cell environment interaction across multiple scales" and "capable of handling spatial atlases with millions of cells" are also core features of DECIPHER. However, the manuscript lacks a systematic comparison between the two tools. Please supplement this critical contextualization:

- A clear comparison of core features (eg: core functions and details for model construction) to clarify its novelty;
- Quantitative evaluations of "runtime efficiency", "performance" and "biological interpretation in real-world data" across datasets of varying sizes and types, to empirically demonstrate your tool's performance advantages;
- A detailed discussion of model design differences. Both your tool and DECIPHER use contrastive learning to reduce runtime and propose its ability on large-scale datasets, but the manuscript fails to elaborate on distinctions in the model design—and how these differences impact efficiency and analytical outcomes.

Response:

We appreciate the reviewers' suggestions. We will explain the following aspects.

1. Model and contrastive learning strategy

CellNiche employs an architecture that does not rely on learning cell-intrinsic molecular identities. The model does not re-learn or reconstruct molecular features; instead, any upstream representation (raw expression, PCA embeddings, scVI embeddings, protein markers, cell-type labels, etc.) is treated directly as a fixed molecular descriptor. The contrastive learning module in CellNiche is dedicated to constructing cellular microenvironment representations. Specifically, positive and negative pairs are defined based on co-localization or co-expression within the spatial neighborhood, allowing the model to pull together cells that share similar microenvironments and push apart those from distinct ones. This contrastive learning operates between cells and does not involve self-contrastive augmentation of the same cell. This design reduces model complexity and training time, improves scalability, and allows flexible

integration with a wide range of upstream single-cell feature representations.

In contrast, although DECIPHER also adopts a contrastive learning framework, its formulation is fundamentally different. In DECIPHER, positive pairs are defined as two augmented views of the same cell obtained through random dropout ("Two augmented views are generated for each cell through random dropout."). During training, DECIPHER maximizes the similarity between views of the same cell while minimizing similarity across different cells ("During training, the model maximizes the similarity between the two views of the same cell while minimizing the similarity between the views of different cells."). Thus, DECIPHER performs self-contrastive learning on molecular identity, whereas CellNiche performs environment-level contrastive learning.

2. Performance

We evaluated DECIPHER alongside other models across four datasets in the revised manuscript (**Fig. 2, 3**) and an additional mouse embryo stereo-seq dataset in the revised supplementary information (**Supplementary Fig. 7**).

3. Compatibility with diverse molecular input representations

In supplementary experiments (**Supplementary Fig. 6**), we compared CellNiche with STAGATE, GraphST, DECIPHER, and BANKSY under four different input representations. We found that DECIPHER failed to produce meaningful embeddings when using PCA or scVI embeddings as input and similarly failed on the mouse spleen CODEX proteomics dataset (29 protein markers). We speculate that this may relate to its reliance on dropout-based augmented views: when molecular representations are low-dimensional, information-poor, or noisy (e.g., PCA with 50 dimensions, scVI with 10 dimensions, or protein markers with 29 dimensions), the dropout operation may remove too much information, leading to inadequate augmented views and failure of contrastive learning.

4. Time cost

We compared runtime cost for DECIPHER in the revised manuscript (**Fig. 1c**) and in the revised supplementary information (**Supplementary Fig. 9**)

2. Also, in the benchmark analysis, the manuscript only includes GNN methods (e.g., STAGATE, GraphST) and some Transformer-based methods (e.g., CellPLM, LargeGT), but omits other large-model studies in the current spatial omics field (such as scGPT-spatial, the spatial extension of scGPT mentioned in the manuscript). Such large models typically exhibit stronger cross-dataset generalization and feature extraction capabilities, making them important competitors to CellNiche for atlas-level data analysis. Without comparisons with these methods, the performance advantages of CellNiche in large-scale, heterogeneous data cannot be fully

validated. We suggest that the authors supplement comparative experiments with mainstream spatial omics large models like scGPT-spatial, using consistent evaluation metrics (e.g., ARI, AMI, Macro-F1) and datasets. This will verify CellNiche's competitiveness in the context of large models while analyzing its relative advantages in computational efficiency and microenvironment identification accuracy.

Response:

We thank the reviewer for highlighting the importance of comparing CellNiche against recent spatial omics large-model frameworks. In the revised manuscript, we have added benchmarking results for scGPT-spatial, the spatial extension of scGPT (**Fig. 3f-j**).

Because scGPT-spatial (as well as scGPT itself) is pretrained exclusively on human single-cell datasets, it cannot be applied to the original three benchmark datasets in our manuscript. To enable a fair comparison, we incorporated an additional human spatial transcriptomics dataset into our benchmarking panel and evaluated scGPT-spatial using the same metrics and evaluation protocols as other methods.

3. The manuscript presents three versions of CellNiche (CellNiche_pheno, CellNiche_expr, CellNiche_decoder), but their performance lacks consistent superiority (e.g., the default CellNiche_pheno performs best on the osmFISH dataset, while CellNiche_expr performs best on the CODEX dataset). We recommend that the authors conduct an in-depth analysis or discussions of the reasons for performance differences across versions and provide clear guidelines for version selection (e.g., which version is preferred for gene expression data, and which is suitable for phenotypic annotation data).

Response:

We thank the reviewer for the insightful comment. We would like to clarify that CellNiche is not designed as three independent "versions," but rather as a single contrastive-learning framework that can flexibly accept different forms of cell-level molecular representations as input. This design principle—consistent with the responses provided to Reviewer #1 (Major Comment 1) and Reviewer #2 (Major Comment 4)—is central to our method: CellNiche treats molecular identity as an external representation space rather than a target to be re-learned. As a result, different molecular features (cell-type labels, raw expression, PCA/scVI embeddings, protein markers, etc.) can all serve as compatible inputs without modifying the model architecture.

Performance differences observed across datasets do not reflect multiple CellNiche "variants," but rather the biological properties of the input feature spaces: raw expression profiles in single-cell data are typically high-dimensional, sparse, and noisy. In microenvironment-scale analyses, training with raw expression profiles can sometimes introduce spatially irrelevant noise. In

contrast, gene expression profiles are often well-interpreted using discrete cell type or cell state identifiers, which can be obtained through expert annotation or computational methods, and the quality of these works is improving with advancements in the field. Therefore, high-quality cell molecular identity labels provide a denoised, compact representation that may be more conducive to directly capturing the biological granularity most relevant to spatial niche structure.

Other modalities (e.g., ATAC-based embeddings, multi-omics signatures) are expected to emphasize yet different biological aspects of the niche. Systematically characterizing such modalities is beyond the scope of the present study but remains an interesting future direction.

Thus, observed performance differences across datasets arise from the properties of the molecular feature space, rather than from architectural changes in CellNiche.

Importantly, CellNiche itself remains a single model, and its core contribution lies precisely in allowing such flexibility—rather than prescribing a specific input modality. Users may therefore choose the molecular feature representation that best matches their data characteristics and scientific goals.

4. Clustering is primarily conducted using the K-means algorithm, where the number of clusters is arbitrarily set to match the number of annotated regions. No justification is provided for this choice—for instance, a rigorous biological prior hypothesis is required and how should the clustering number be determined for samples without prior knowledge (also the number is related with different biological label resolutions, how to determine the exact number of clustering)? We suggest:
- Testing alternative clustering algorithms widely used in single-cell/spatial omics (e.g., Leiden, Louvain, just like the results present in Fig. 7);
- Comparing cluster quality across methods using metrics like silhouette score or adjusted Rand index (ARI) to validate the reliability of region segmentation and ensure downstream analysis is robust.

Response:

We thank the reviewer for this constructive suggestion. In the revised manuscript, we have clarified the rationale behind our clustering choices and expanded our evaluation accordingly.

1. Inclusion of multiple clustering algorithms

As recommended, we have supplemented our analysis with results obtained using two widely adopted graph-based clustering methods—Leiden and Louvain. These results have been added to the revised supplementary information (**Supplementary Fig. 2, 3, 4, 5, 7**).

2. Addition of clustering-independent quality metrics

To assess embedding quality independently of the clustering method, we now include Silhouette scores for all benchmark datasets (**Fig. 2,3, Supplementary Fig. 7**). This intrinsic metric provides a direct evaluation of embedding structure and further supports the robustness of CellNiche representations.

3. Rationale for using K-means in the main benchmark evaluation

Our benchmark analyses are designed to evaluate how well each model captures known biological structures, where ground-truth labels are provided by the original studies. In this setting, we chose K-means primarily due to its computational efficiency and neutrality: 1) K-means provides a fast and consistent clustering baseline across datasets of varying sizes, avoiding the substantial runtime overhead associated with Leiden or Louvain—both of which require repeated runs under different resolution parameters. 2) Since these evaluations do not involve biological discovery, but instead quantify alignment to established reference labels, K-means offers a simple and practical approach that avoids introducing additional methodological variability. 3) In contrast, for analyses driven by biological exploration, such as the NSCLC cohort and mouse brain atlas integration, we extensively utilize other clustering strategies, as illustrated in the Results section of this paper.

5. Figure 4 presents inter-patient tumor heterogeneity, but it fails to distinguish whether this heterogeneity arises from "tissue section sampling differences" (e.g., spatial location within the tumor) or "intrinsic sample differences" (e.g., patient-specific genetic background or tumor stage). R1-R3 from the same sample LUAD5 may be adjacent sections and tend to have similar distributions of niches. Meanwhile, the current analysis relies on a small number of samples from the same type of cancer and without pan-cancer coverage, making it difficult to support the claim that the tool "reflecting interpatient heterogeneity in tumor composition." We suggest:
- Incorporating more samples from the same type of cancer/pan-cancer datasets (e.g., breast cancer, colorectal cancer) to expand sample diversity and discussing the potential explanations of the heterogeneity between location differences and sample differences;
- Conducting analyses (if have, e.g., comparing samples from different patients with matched sampling locations, or comparing multiple sections from the same patient) to clarify the source of heterogeneity and enhance the generalizability of results.

Response:

We thank the reviewer for raising this important point. To further clarify the source of tumor heterogeneity, we performed additional analyses on two independent datasets—NSCLC and human colorectal cancer (CRC)—focusing exclusively on tumor cells and evaluating their relative similarity across different spatial or clinical groupings within each embedding space.

1. NSCLC: tumor-cell differences across sections and patients

For the NSCLC cohort, we extracted tumor cells from all sections and computed section-level centroids in four embedding spaces (raw expression, CellNiche_expr, CellNiche_scVI, and CellNiche_pheno). LUAD-5 contains three sections sampled from spatially adjacent regions, whereas LUAD-9 contains two sections with clearly distinct spatial locations (**Supplementary Fig. 13a**), which allowed us to compare: 1) within-patient, spatially adjacent sections (LUAD-5); 2) within-patient, spatially separated sections (LUAD-9); 3) between-patient comparisons.

Table 1. Mean cosine distances between tumor-cell centroids across sections and patients in the NSCLC cohort.

Embedding	Within LUAD-5 (n=3)	Within LUAD-9 (n=1)	Between patients (n=24)
Expression	0.0048	0.0306	0.1686
CellNiche_expr	0.0500	0.8581	1.129
CellNiche_scVI	0.0207	0.5893	1.050
CellNiche_pheno	0.000091	0.00486	1.129

Across all four embeddings, the same qualitative hierarchy was observed: within LUAD-5 < within LUAD-9 < between patients **(Supplementary Fig. 13b)**.

This pattern is consistent with the underlying tissue sampling: LUAD-5 sections originate from a more coherent anatomical region, whereas LUAD-9 sections differ more strongly in spatial context. Because the within-patient sample sizes are extremely small (e.g., within LUAD-9, n = 1 pair), we explicitly treat these NSCLC results as descriptive rather than as formal statistical evidence.

2. CRC: tumor microenvironment differences across region, patient, and risk groups

To complement the limited sample size of the NSCLC cohort, we performed an analogous but more statistically powered analysis in the human colorectal cancer (CRC) CODEX dataset, which includes 35 patients, 138 tumor-containing regions, and two clinical risk groups (CLR/DII). For each embedding, we computed tumor-cell centroids per region and evaluated four levels of comparison: 1) region A/B pairs (same region ID, different TMA); 2) within-patient, different regions; 3) between patients with the same risk group; 4) between patients with different risk groups.

Table 2. Mean cosine distances between tumor-cell centroids across regions, patients and risk groups in the CRC cohort.

Embedding	Region A/B (n=68)	Within patient (n=136)	Between patients, same risk (n=4497)	Between patients, different risk (n=4752)
Expression	0.1209	0.1240	0.1855	0.2029
CellNiche_expr	0.7388	0.6612	0.9094	0.9730
CellNiche_scVI	0.7847	0.6633	0.9149	0.9882
CellNiche_pheno	0.6216	0.6327	0.7534	0.8219

Across all four embeddings, a consistent internal hierarchy emerged: region A/B ≈ within-patient < between-patient (same risk) < between-risk **(Supplementary Fig. 14b)**.

Mann–Whitney U tests confirmed that distances between patients (same risk) were significantly larger than within-patient distances, and that distances between different risk groups were further increased, for all four embeddings. Although A/B pairs share region IDs, tissue images reveal they are not strictly co-localized **(Supplementary Fig. 14a)**, explaining the lack of significant difference between Region A/B and within-patient.

3. Overall summary and limitations

In both datasets, the results reveal a stable hierarchy (within-region/patient < across patients < across clinical strata), consistent with biological expectations of tumor heterogeneity. NSCLC findings are descriptive due to limited within-patient replicates and spatial asymmetry. CRC provides stronger support for the order, robust across embeddings. However, neither dataset allows full disentanglement of sampling-location effects from intrinsic differences, owing to limited section numbers (NSCLC) and non-aligned A/B regions (CRC). Expanding to pan-cancer datasets (e.g., breast or colorectal cancer cohorts with matched sampling) could address this in future work, but is beyond the current study's scope focused on demonstrating CellNiche's scalability in spatial omics. These analyses reinforce our qualitative claim of inter-patient heterogeneity while acknowledging sampling limitations.

Supplementary Fig 13 (Additions):

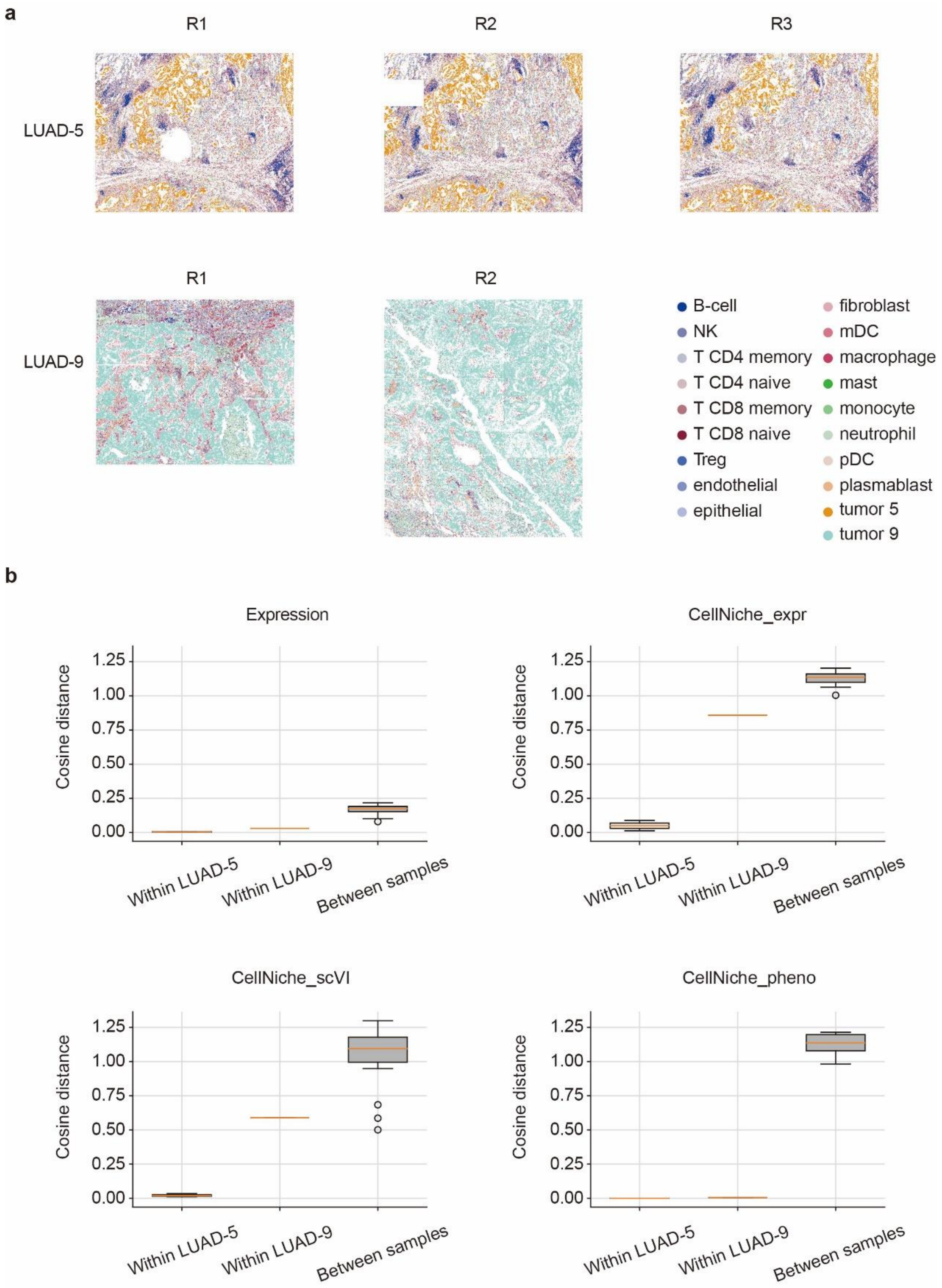

Supplementary Fig. 13: Spatial distribution and tumor microenvironment heterogeneity in NSCLC datasets.

a, Cell-type maps for LUAD-5 (three sections: R1–R3) and LUAD-9 (two sections: R1–R2). Each point represents a single cell, colored by its cell-type annotation (legend shown on right). LUAD-5 sections exhibit highly consistent spatial tissue composition, while LUAD-9 sections

display noticeable spatial divergence. b, Cosine distances between tumor-cell centroid embeddings across sections, evaluated in four representation spaces: Raw expression, CellNiche trained with expression input (CellNiche_expr), CellNiche trained with scVI embeddings (CellNiche_scVI), and CellNiche trained with cell-type labels (CellNiche_pheno). For each embedding space, distances were computed for three groups: within LUAD-5 (three technical sections), within LUAD-9 (two technical sections), and between samples. Boxplots summarize the distribution of pairwise cosine distances based on tumor-cell centroids in the corresponding embedding space.

Supplementary Fig 14 (Additions):

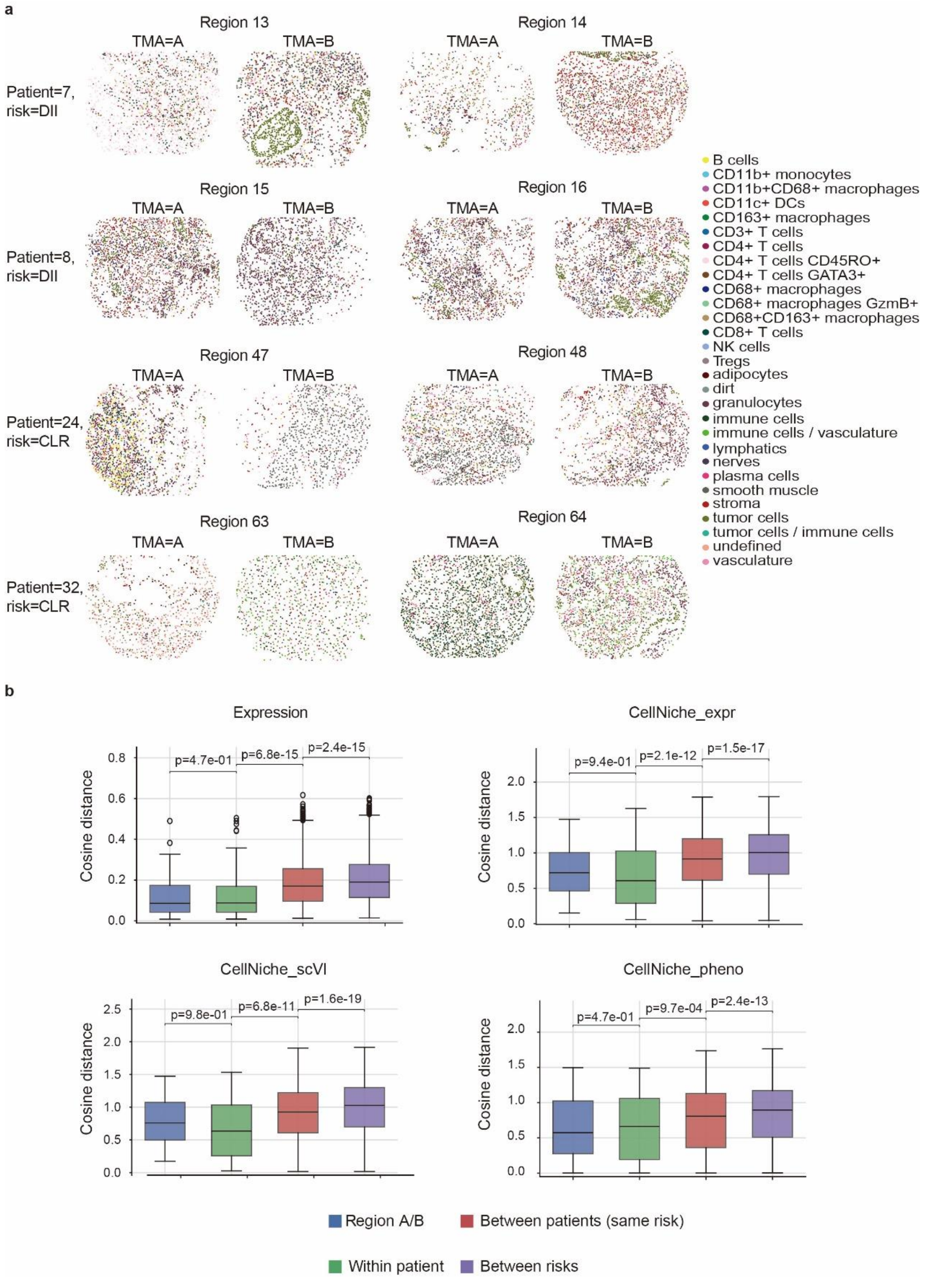

Supplementary Fig. 14: Spatial distribution and tumor microenvironment heterogeneity in CRC datasets.

a, Spatial cell-type maps for representative tumor regions from CLR and DII patients. For each

region, two adjacent sections (TMA-A and TMA-B) are shown. Each point denotes a single cell, colored by its supervised cell-type annotation (legend shown on right). Although sections share the same region identifier (e.g., reg013_A vs. reg013_B), their spatial organization can differ substantially. b, Cosine distances between region-level tumor-cell centroid embeddings across four representation spaces: Raw expression (RawX), CellNiche trained with expression input (CellNiche_expr), CellNiche trained with scVI embeddings (CellNiche_scVI), and CellNiche trained with cell-type labels (CellNiche_pheno). Distances were computed across four groups: (i) region A/B pairs from the same region_base, (ii) different regions within the same patient, (iii) different patients with the same CLR/DII risk classification, and (iv) patients with different risk classifications. Boxplots summarize the distribution of pairwise cosine distances in each embedding space.

6. The manuscript provides no explanation of how the tool analyzes or compares data at different granularities (e.g., cell subtype-level, cluster-level). Nor does it interpret the connections or differences between results at these granularities. Please supplement:

- Strategies for converting data between granularities, especially when addressing and comparing datasets with different label resolutions (which resolution should we choose and how to maintain the robustness of comparisons);
- A comparative interpretation of analytical outcomes across granularities for the same dataset, to demonstrate the tool's flexibility in multi-scale analysis.

Response:

We thank the reviewer for raising the important question regarding *granularity*, including how CellNiche handles inputs defined at different molecular or annotation resolutions, and how analytical outcomes at different granularities should be interpreted.

A key conceptual feature of CellNiche—consistent with the responses provided to Reviewer #1 (Major Comment 1) and Reviewer #2 (Major Comment 4)—is that the model does not re-learn cellular molecular identity internally. Instead, CellNiche directly operates on externally supplied cell-level representations, which can come from: raw gene expression、

This design means that CellNiche naturally treats these inputs as *alternative molecular descriptors on the same cells*, without imposing constraints on the granularity (fine-grained cell states vs. coarse cell types).

Because the model only learns spatial microenvironment embeddings, not the identity embeddings themselves, it is inherently agnostic to the granularity of the upstream representation. Thus, no special conversion, harmonization, or alignment between granularities is required at the model level.

While we also evaluated alternative input representations in the extended benchmarking experiments (**Supplementary Fig. 6, Supplementary Fig. 8**), discussing the impact of different molecular identity features on microenvironment representation is not the focus of this work.

7. When comparing different methods, the manuscript should use the original datasets reported in the respective methods. This introduces bias, as dataset characteristics can influence method performance. We recommend adhering to standard benchmarking practices:

- Use the "native validation datasets" from each compared method's original study;
- Alternatively, well-recognized public spatial multi-omics benchmark datasets are also acceptable (e.g., human non-small cell lung cancer dataset) to ensure fair comparisons, while more recent methods should be included.

Response:

We thank the reviewer for emphasizing the importance of fair and transparent benchmarking. Rather than selecting datasets arbitrarily, we deliberately chose publicly available datasets that are widely used, biologically meaningful, and technically representative of the main regimes in which spatial models are typically evaluated.

1. Mouse somatosensory cortex osmFISH

This dataset represents a canonical spatial structure benchmark with high-quality cell-type labels and moderate cell numbers. It has been extensively used to evaluate spatial clustering and niche segmentation. Because of its clear laminar architecture and low technical noise, osmFISH is well suited as a foundation benchmark for assessing all methods.

2. Mouse spleen CODEX

This dataset characterizes a typical spleen structure and provides a test of modality robustness, evaluating whether methods generalize beyond transcriptomics. Importantly, this dataset was also used in the CytoCommunity study, providing consistency with prior benchmarking.

3. Mouse brain STARmap PLUS

Earlier benchmarks often rely on coarser mouse brain labels; given the improved resolution and data quality of mouse brain STARmap PLUS dataset, we consider it necessary to update evaluations to reflect modern datasets and better assess scalability and fine-scale niche discrimination.

4. Human colorectal cancer (CRC) CODEX

The CRC dataset is a supplementary dataset in this revised version. Firstly, it is a human cell dataset (which can be evaluated against the supplementary method scgpt-spatial). Secondly, its pathological state is tumor-based, reflecting the model's ability to characterize the cellular microenvironment under different pathophysiological states.

5. Mouse embryo Stereo-seq

We additionally include a whole-transcriptome Stereo-seq mouse embryo datasets in the revised supplementary information. This is used to represent spatial genomics data with whole transcriptome sequencing technology.

Our intention is not to bias performance toward any particular method, but to ensure that the benchmarking framework captures the diversity of real spatial omics applications—across species, modalities, physiological and pathological states, annotation granularities, and data scales. All datasets are fully public and widely used in the community, ensuring transparency, reproducibility, and methodological fairness.

8. There are many typos or wrong statements in the current manuscript:

- The claim that "One notable microenvironment, C0, showed strong enrichment for lymphoid-like cells... and was predominantly present in LUAD-5 and LUSC-6 (Fig. 4b)" is inconsistent with the data in Figure 4b. Please verify and correct this. Not LUSC-6?
- "CellChat analysis predicted strong MHC-I signaling interactions between CD8 naïve T cells and other cell types within cluster C4 (Fig. 4d)" is incorrect; the interactions actually occur in cluster C0. Correct the cluster ID and figure annotation.
- The statement "Similar trends were observed under coarser clustering resolutions... (Fig. 5a, 5d, 5e)" cannot be validated, as no corresponding results exist in Figure 5! Either add the missing figures or revise the conclusion.
- "Similarly, we successfully transferred 107 subregion annotations with 91.3% accuracy (Fig. 7e, Fig. S6a, b)" contains an incorrect figure citation; Figure 7e does not exist. Verify and update the reference.
- "Additional Leiden clustering (41 clusters) further confirmed the subregion refinement (Fig. 8b)" is wrong; the relevant result is in Figure 7b, not 8b. Correct the figure number.
- Thoroughly check and revise the entire manuscript, including but not limited to the errors above.

Response:

We thank the reviewer for identifying these typographical errors, inconsistencies, and incorrect statements. We appreciate the detailed feedback, as it helps ensure the manuscript's accuracy and clarity. We have carefully verified each point against the data and figures, made the necessary corrections in the revised manuscript, Below, we outline the specific revisions for each item raised:

1. Regarding the claim that "One notable microenvironment, C0, showed strong enrichment for lymphoid-like cells... and was predominantly present in LUAD-5 and LUSC-6 (Fig. 4b)": Upon verification, this was a labeling inconsistency; Figure 4b shows C0 predominance in LUAD-5 and LUAD-12 (not LUSC-6, which was a typographical error in the text). We have corrected the text to: "... and was predominantly present in LUAD-5 and LUAD-12 (**Fig. 4c**)."
2. For the statement "CellChat analysis predicted strong MHC-I signaling interactions between CD8 naïve T cells and other cell types within cluster C4 (Fig. 4d)": This was an error in cluster identification; the interactions occur in C0, as shown in the data. We have revised the text to: "CellChat[42] inferred a broad set of transcriptionally putative MHC-I interactions involving CD8 naïve T cells and multiple additional cell types within C0 (**Fig. 4d**)." Additionally, we have updated the figure legend and annotations in Fig. 4d to explicitly reference C0 for consistency.
3. Concerning "Similar trends were observed under coarser clustering resolutions... (Fig. 5a, 5d, 5e)": Upon review, this was an incorrect cross-reference. We have revised the

text to: “Similar trends were observed under coarser clustering resolutions, where neutrophils and fibroblasts in LUAD-12 co-localized within C7, while those in LUAD-9 mapped to distinct niches (C9 and C10, respectively) (**Supplementary Fig. 12a, d, e**).”

4. For “Similarly, we successfully transferred 107 subregion annotations with 91.3% accuracy (Fig. 7e, Fig. S6a, b)”: Figure 7.e does not exist; this was a typographical error. We have corrected the text to: “Similarly, we successfully transferred 107 subregion annotations with 91.3% accuracy (**Supplementary Fig. 15a, b**)”.
5. Regarding “The additional Leiden clustering (41 clusters) further confirmed the refinement of the subregions (Figure 8b)”: This is an incorrect figure number. We have refined this part of the experiment and revised the corresponding descriptions and figures.

We appreciate the reviewer's careful reading and drawing our attention to these inaccuracies. We have corrected all the issues mentioned and apologize for these oversights. In the revised version, we have corrected all listed errors and systematically re-checked the entire manuscript (including figure references and annotations) to ensure consistency and accuracy.

9. Figures 7a and 7b show marker gene expressions across different tissue sections, but no explanation is provided for why "different marker genes are used for similar anatomical regions across sections". If these regions are intended to be similar, justify the use of distinct markers, and clarify the biological rationale for marker selection.

Response:

We thank the reviewer for pointing out the need to clarify why different marker genes were used for anatomically related regions across sections. In the revised manuscript, we now explicitly explain both the technical constraints and the biological rationale underlying our marker selection.

First, the four mouse brain atlases we integrate were generated using different experimental platforms and gene panels, leading to only partial overlap in the measured gene sets. As a result, it is generally not possible to apply an identical set of marker genes to the same anatomical region across all sections. Instead, for each atlas and slice, we select marker genes according to two criteria: (i) the gene must be present in the corresponding panel, and (ii) it must be biologically appropriate for the region or subregion of interest based on prior literature. This rationale is now described in the Results text accompanying Figure 7.

Second, in the revised version we explicitly document, in the Figure 7 legend and main text, the biological basis of each marker set (e.g. Cldn11/Lpar1/Gjc3 for FbTrt; Foxp2/Sox4 and Tshz1/Sox11 for the two CNU_13 subdomains), together with citations supporting their regional or laminar specificity.

Finally, to demonstrate that these refinements do not solely reflect a single, discrete clustering choice, we performed Leiden clustering on the CellNiche embeddings across a range of resolutions. For the FbTrt refinement, the putative FbTrt cells consistently form a coherent, spatially contiguous domain across resolution settings; for the CNU_13 subdivision, the two subregions remain separable even under coarse clustering (few global clusters) and persist as distinct groups as the resolution is decreased (**Supplementary Fig 16a, b**). These analyses support that the proposed refinements are robust features of the continuous embedding space and are consistent with the marker-based anatomical interpretation.

Supplementary Fig 16 (Additions):

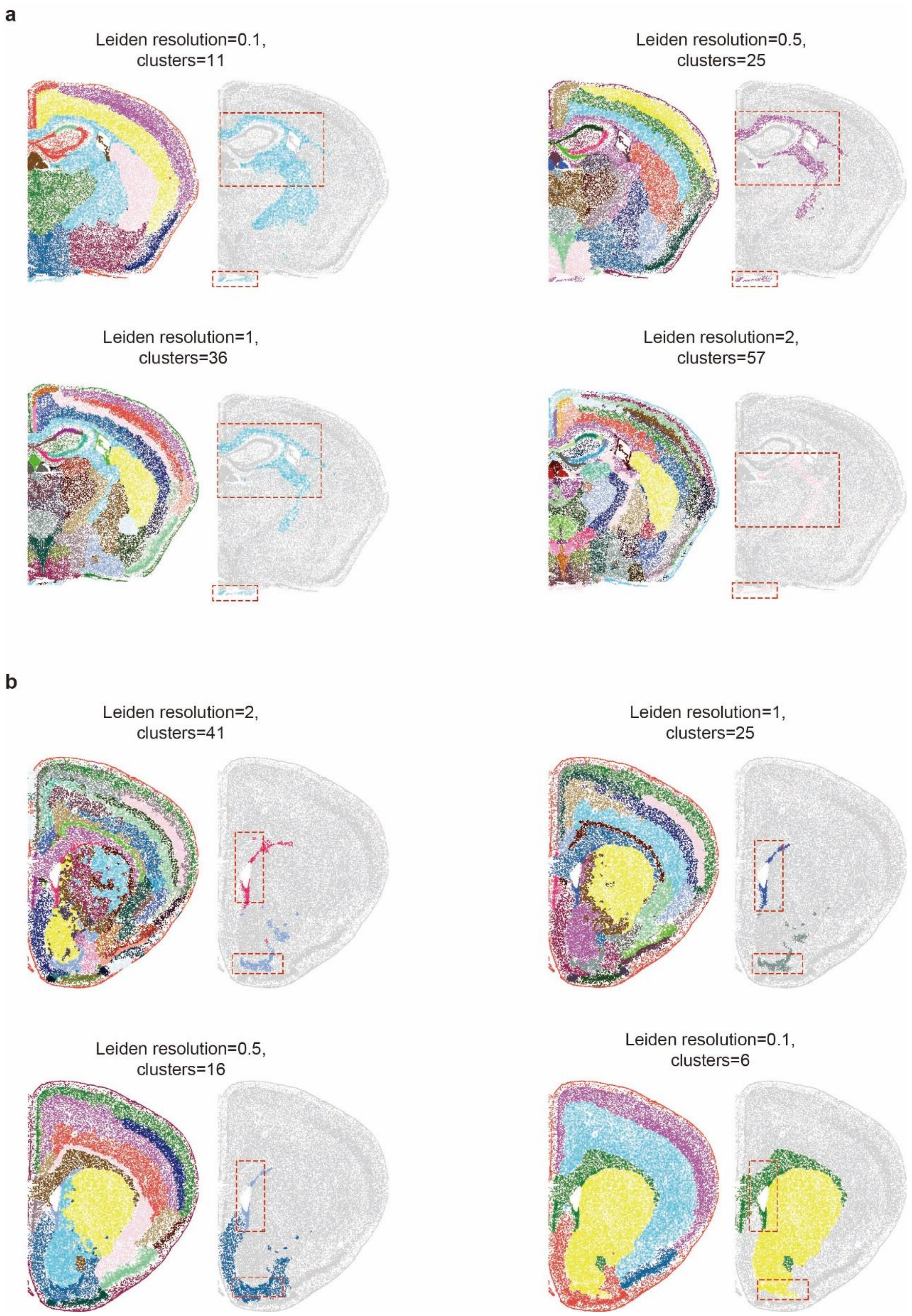

Supplementary Fig. 16: Robustness of the FbTrt refinement and the CNU_13 bipartition across Leiden resolutions.

a, Leiden clustering was performed on CellNiche embeddings for the Atlas 1 well05 section, using a range of resolution parameters (as indicated). Left panels show the full Leiden partition;

right panels highlight the cells assigned to FbTrt by cross-atlas label transfer. Across resolutions (from coarse to fine partitions), FbTrt-labelled cells consistently form a coherent and spatially contiguous domain. b, Leiden clustering on CellNiche embeddings was evaluated across multiple resolutions for the Atlas 1 well03 section to test the stability of the proposed CNU_13 subdivision. Even under coarse clustering (few global clusters), the two CNU_13 subdomains remain separable in the embedding-derived partitions

Reviewer #3 (Remarks on code availability):

The code provides a detailed README file with instructions for installing and running the application. I can easily install the code.

Response:

Thank you for this summary!

Point-by-point response

Reviewer #1 (Remarks to the Author):

The additional analyses have significantly improved the manuscript, and the authors have addressed most of the questions. I have a few comments.

• The statement that CellNiche "decouples molecular identity representations from spatial proximity modeling" may be overly definitive. It would be more precise to describe how these two components are integrated or parameterized. Complete decoupling could lead to biologically inconsistent results; for instance, if two spatially proximal spots exhibit distinct expression profiles, their classification into the same or different niches should logically depend on their molecular identities. A model that strictly decouples these features might fail to capture the nuanced interdependence required to define a niche accurately.

Response:

We sincerely thank the reviewer for this insightful clarification. We agree that the term "decouples" could be misinterpreted as implying a complete functional separation. In CellNiche, molecular identity is not removed from niche inference. Rather, we separate the modeling roles of identity features and spatial proximity learning.

Specifically, CellNiche treats cellular identity (e.g., expression profiles or cell-type annotations) as predefined inputs (fixed features) rather than targets to be reconstructed. This architectural choice focuses the model's learnable capacity on capturing spatial proximity and organization while inherently respecting the provided molecular context. Consequently, if two spatially proximal cells have distinct molecular identities, their niche representations will naturally differ because the model operates on these distinct input features.

We have revised the manuscript text to reflect this more precise description and to avoid an overly definitive claim of "complete decoupling." Specifically, we now describe CellNiche as separating identity feature specification from spatial proximity learning, and emphasize that identity features are treated as predefined inputs rather than reconstruction targets, enabling spatial characterization while preserving the provided identity information

Abstract section (Line 41):

"

Importantly, CellNiche separates the modeling roles of molecular identity and spatial context by treating cellular identity features (e.g., expression-derived features, learned embeddings, or standardized cell-type annotations) as fixed inputs rather than reconstruction targets, enabling spatial characterization of cell–cell relationships while preserving identity information in the

input features.

”

Introduction section (Line 127):

“

CellNiche avoids redundant reconstruction of molecular identity within the spatial module by treating cellular identity features as predefined inputs (e.g., expression-derived features, learned embeddings, or standardized cell-type annotations), rather than reconstruction targets.

”

Discussion section (Line 466):

“

Distinct from previous methods, CellNiche improves scalability to the atlas level by mimicking the cellular niche concept, placing greater emphasis on learning spatial proximity patterns through a cell-centric and batch-efficient strategy, and separating identity feature specification from spatial proximity learning.

”

Discussion section (Line 478):

“

Furthermore, CellNiche treats molecular identity as a fixed input and focuses the learnable component on spatial proximity modeling, enabling the characterization of cell–cell relationships within the context of a given molecular identity representation.

”

- The manuscript does not mention the use of Louvain clustering method in the main text, although these analyses are presented in Supplementary Figures 2 – 5. It is highly recommended to add it in Methods section.

Response:

We appreciate the reviewer's careful reading and the suggestion to include the clustering methodology. Following this advice, we have explicitly incorporated the description of the Louvain clustering method into the **Methods** section

Methods section (Line 748):

“

Furthermore, to assess the robustness of each model across clustering strategies, we conducted supplementary comparisons on representative slices from five datasets: one slice from the cortical osmFISH dataset, the “BALB/c-1” slice from the mouse spleen CODEX dataset, the coronal “well11” slice from the mouse brain STARmap PLUS dataset, the “reg007_A” slice from the human colorectal cancer CODEX dataset, and the “E16.5_E2S4” slice from the mouse embryo Stereo-seq dataset. We evaluated all methods using three clustering approaches—K-means, Leiden, and Louvain. For K-means, we set the number of clusters to match the number of label categories in each dataset. For Leiden and Louvain, we used the Scanpy implementations (scanpy.tl.leiden and scanpy.tl.louvain) and tuned the resolution parameter so that the resulting number of clusters matched the number of label categories.

”

- The analyses for supplementary figures 6 and 7 has not been described or integrated into the manuscript, despite the presence of related results.

Response:

We thank the reviewer for pointing out this omission. To better align with the presentation flow of the main text, we have reorganized the sequence of the Supplementary Information. Accordingly, the analyses originally presented as Supplementary Figures 6 and 7 have been repositioned as Supplementary Figures 8 and 3, respectively, and are now seamlessly integrated into the relevant subsections of the **Results** section.

Results section (Line 258):

“

Moreover, consistent with the design of CellNiche, we observed that CellNiche maintained competitive and stable advantages over other methods when using molecular identity features from different sources as inputs (raw expression, PCA/scVI-derived embeddings, or cell-type features) (Supplementary Fig. 8).

”

Results section (Line 215):

“

In addition to targeted gene-panel datasets, we also evaluated CellNiche on a whole-transcriptome spatial transcriptomics dataset (mouse E16.5 whole embryo Stereo-seq data, comprising 12,215 cells and 15,120 genes) and obtained competitive results with consistent

performance trends (Supplementary Fig. 3).

"

- Several other subfigures and supplementary figures are not explicitly referenced in the main text. All figures and supplementary materials should be cited and discussed to guide the reader through the results.

Response:

Thank you for this helpful suggestion. In the revised manuscript, we systematically checked the full set of main and supplementary figures and ensured that all subfigures and supplementary figures are now explicitly cited in the main text at the appropriate locations, with brief accompanying descriptions to guide the reader through the results.

- Further clarification of the CRC dataset (around line 275) would be beneficial.

Response:

Thank you for the suggestion. We have added additional clarification for the CRC dataset description in the revised manuscript.

Results section (Line 271):

"

we evaluated CellNiche on a human colorectal cancer (CRC) spatial proteomics dataset generated using CODEX. Specifically, we applied CellNiche to the section "reg007_A" from the diffuse inflammatory infiltration (DII) risk group, which contains 3,077 spatially resolved cells profiled with 58 protein markers. The original study provided annotations for 17 cell types and 8 regions (Fig. 3f, g, Supplementary Fig. 1d).

"

- In Figure 4f, cluster C17 is missing. Please verify whether this is a typographical or labeling error and correct it accordingly.

Response:

Thank you for catching this. We verified the labeling in Fig. 4f and confirmed that the missing "C17" was a typographical issue—this cluster should be "C16." We have updated the manuscript files to ensure consistency throughout.

Results section (Line 323):

“

For instance, neutrophils and fibroblasts had similar proportions in LUAD-9 and LUAD-12 (Supplementary Fig. 11c) but were enriched in different microenvironments: in LUAD-12, both were co-enriched in C6 and C14, whereas in LUAD-9, fibroblasts were scattered across C1, C3, C9, and C14, and neutrophils were concentrated in C16 (Fig. 4f).

”

- Figure 4h lacks x- and y-axis tick labels. These should be added to improve interpretability.

Response:

Thank you for the suggestion. We have updated Fig. 4h to include both x- and y-axis tick labels in the revised version.

Reviewer #2 (Remarks to the Author):

I thank the authors for the response.

1) I thank the authors for including the additional spatial omics methods, but I couldn’t get an indication of how these methods compare with their method based on their response. In addition, as previously suggested, to ensure the authors are not cherry-picking data, it would be useful to also show the performance of their method on datasets which previous methods have shown to be best-performing.

Response:

We thank the reviewer for this constructive critique. To address the concern regarding potential data cherry-picking, we have provided further explanations and clarifications, as well as a supplementary experiment to illustrate our comparison of previous state-of-the-art (SOTA) methods on their dominant datasets.

1) CytoCommunity. CytoCommunity uses the mouse spleen CODEX dataset for demonstration, and its paper reports AMI and Macro-F1. In our independent experiments on the same dataset, we obtained scores comparable to those reported in their original study. Notably, on this dataset, CytoCommunity consistently emerged as one of the strongest baselines, typically ranking second only to CellNiche across multiple metrics.
2) GraphST. While the original GraphST paper applied their method to the mouse embryo dataset without providing explicit quantitative metrics, our benchmarking shows that GraphST is indeed highly competitive on this platform. Under our evaluation protocol, GraphST performed comparably to CellNiche and outperformed the majority of other baseline methods.
3) Banksy and STAGATE. Both Banksy and STAGATE were evaluated on the STARmap mouse visual cortex dataset (comprising 1,207 cells, 1,020 genes, and 7 annotated regions; accessed via http://clarityresourcecenter.org), with the Banksy paper reporting their ARI scores as the top two among compared methods. To ensure our evaluation was not influenced by dataset preference, we benchmarked CellNiche, Banksy, and STAGATE on this exact dataset using extended metrics and clustering strategies. Under Leiden clustering, our obtained ARI values for Banksy and STAGATE are broadly consistent with those in the Banksy paper. CellNiche achieved comparable Macro-F1 to these methods while demonstrating superior competitiveness on the remaining metrics. (see figure below).
   It is worth noting that because this validation focused only on the superior methods (Banksy and STAGATE) for the STARmap mouse visual cortex dataset and did not cover all the baseline models introduced in our main text and supplementary materials, we did not include these results in the formal manuscript to maintain consistency and conciseness

in the benchmarking scope.

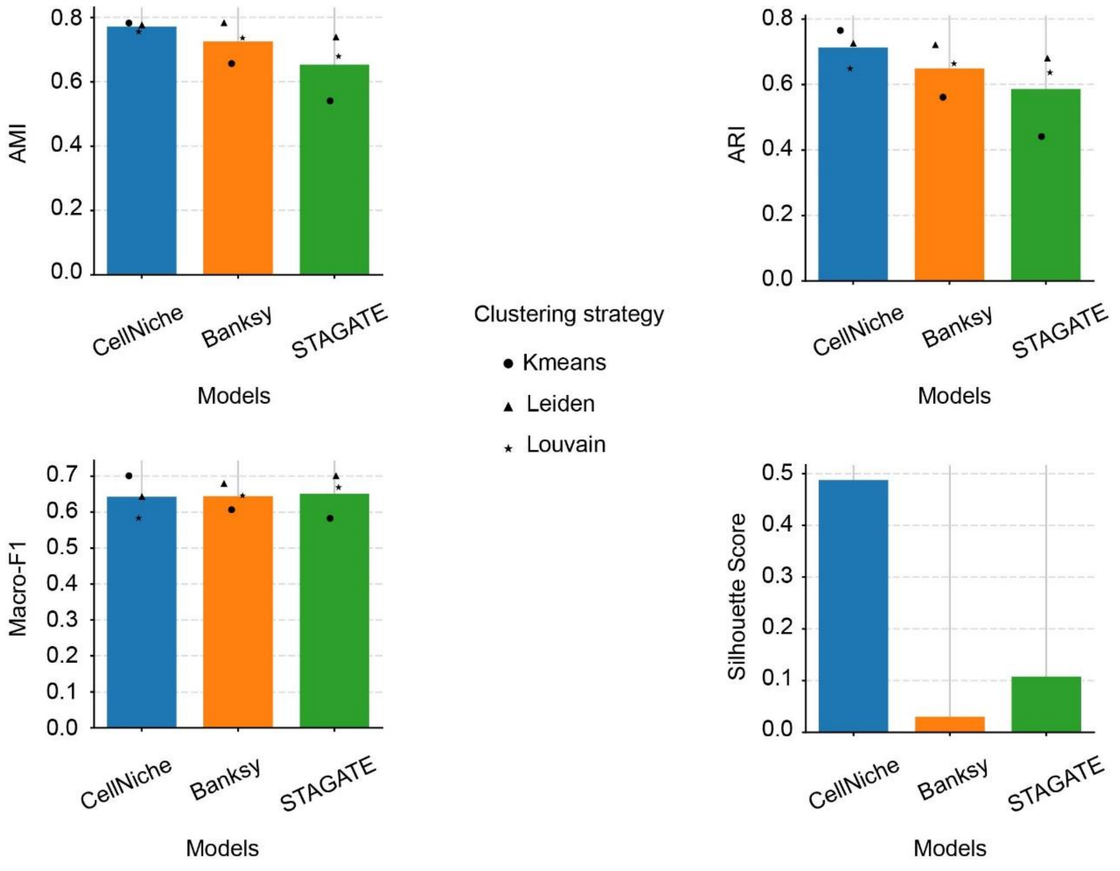

4) DECIPHER. When attempting to reproduce evaluations on the two real-world datasets highlighted in the DECIPHER paper (MERFISH mouse brain and Xenium breast tumor), we noted discrepancies in annotation granularity between the publicly available data and the figures in their publication (e.g., the public MERFISH annotations contain 8 categories, while the DECIPHER figure shows 4). As these differences likely stem from custom post-processing or disparate dataset versions not aligned with public releases, we excluded these specific instances from our benchmark to avoid an inequitable comparison. Furthermore, our application section incorporates MERFISH mouse brain datasets derived from the same technology but from independent batches. This demonstrates that CellNiche remains competitive on datasets closely aligned with its primary application setting.

2) Thanks for clarifying the batch effects issue. First, the manuscript would be more clear if there is consistency with how "batch" is used, as the definition of "batch" in the paper seems confusing, as the authors talk about "batch sampling", "batch processing", "microenvironment batch", and "batch effects"? Does "batch" mean the same thing in all these cases? It'll be good if the authors make it explicitly clear somewhere. Second, the

authors should indicate in the response what changes were made from the previous version (e.g., old version vs new version). Eg., in the previous version they talk about Cell Niche "effectively mitigating batch effects for cross-dataset analysis", and now they talk about "batch effects… handled flexibly upstream". This seems like a conceptual shift, and it's not clear if the authors have changed their method in the revised version to accommodate this change, or are simply now presenting a different interpretation of batch effects in relation to their method.

Response:

We greatly appreciate your suggestions.

First, to eliminate any ambiguity, we have systematically standardized the usage of "batch" throughout the revised manuscript. We now distinguish between two fundamentally different concepts:

- Mini-batch: Refers exclusively to the computational subsets used during stochastic gradient descent optimization.
- Batch effects: Refers to the unwanted technical variations arising from different samples, platforms, or experimental conditions.

We have updated related sections to ensure these terms are used consistently, removing confusing variants such as "batch processing" or "microenvironment batch."

Second, in the first revised version, we did not make any adjustments to the model itself. The refinements in our description and results presentation were made after considering all the reviewers' comments, to emphasize our design philosophy and avoid unnecessary misunderstandings. Regarding your question about "addressing batch effects," we did not specifically emphasize its relationship to our design in the submitted version, but in the first revised version, we more specifically pointed out the flexibility in addressing it in our implementation.

3) Regarding the cellNiche variants, as with the previous comment, it would be useful (and appropriate) if the authors mention any changes they made from the original submitted manuscript in the reviewer response. For example, the previous Figure 2 had figures showing different variants of celNiche (eg., pheno, expr, decoder), but now they are not shown in the current updated figure. It's not clear what the reason for this and what the authors are now showing in their paper (Eg., pheno, expr or decoder?), making it hard to judge whether they have addressed the comments.

Response:

Regarding the CellNiche variants, we appreciate the reviewer's request for clarity about changes from the original submission.

In the original version, we presented three variants of CellNiche: CellNiche_pheno, CellNiche_expr, and CellNiche_decoder. CellNiche_pheno was our default implementation. CellNiche_expr differs from CellNiche_pheno only in the choice of input identity features, while the model architecture is the same. In contrast, CellNiche_decoder extends the CellNiche framework by adding an additional decoder and an expression reconstruction loss; we initially included it to explore the potential impact of architectural/loss-function choices. However, we realized that presenting CellNiche_decoder as a "variant" could inadvertently confuse readers (e.g., suggesting it is an alternative version of CellNiche), and a rigorous treatment of this extension would require additional analyses that are beyond the scope and focus of the current work.

Therefore, in the revised manuscript, we report results using CellNiche_pheno only (referred to as "CellNiche" throughout the main text to avoid ambiguity). To avoid the impression that "each input modality constitutes a different model variant," we moved the evaluation of input compatibility (e.g., expression, PCA/scVI-derived embeddings, etc.) to the Supplementary Information. We also removed the CellNiche_decoder results from the revision. Overall, the results shown in the revised manuscript correspond to CellNiche_pheno from the original submission, and this re-organization is intended to reduce potential confusion and better emphasize the main contributions of the study.

Reviewer #3 (Remarks to the Author):

The authors have addressed all major concerns raised in my initial review. A few minor suggestions for further improvement are noted below:

1. Related to my previous comment #5, I could not find the CellNiche’s spatial niche proportions for samples of CRC cohort. Whether the results are consistent with the tumor microenvironment differences across region, patient, and risk groups.

Response:

We thank the reviewer for this suggestion. To address this, we have included a detailed analysis of the niches inferred by CellNiche within the CRC cohort in the revised Supplementary Information.

We calculated the proportion of cells assigned to each CellNiche cluster (C0–C12) for each patient. These compositions are visualized using stacked bar plots, grouped by clinical risk (CLR vs. DII), which reveal both inter-patient heterogeneity and group-specific trends.

To further quantify these patterns, we compared niche-composition vectors using cosine distance across four comparison strata. The observed distance gradient (within region/patient < between patients < between risk groups) is highly consistent with the patterns identified in our tumor-cell embedding analysis, validating the biological relevance of the inferred niches.

To facilitate biological interpretation, we summarized risk-associated niche shifts using the $\log_2$ fold change of proportions between DII and CLR groups. Combined with cell-type enrichment analysis, we found that C1 is significantly enriched in tumor cells and C12 is enriched in granulocytes, both of which are prominently associated with the high-risk (DII) microenvironment. Conversely, C6 and C9 are more representative of the immune/stromal environments, showing relative over-representation in the CLR group.

Supplementary Fig. 14 (Additions):

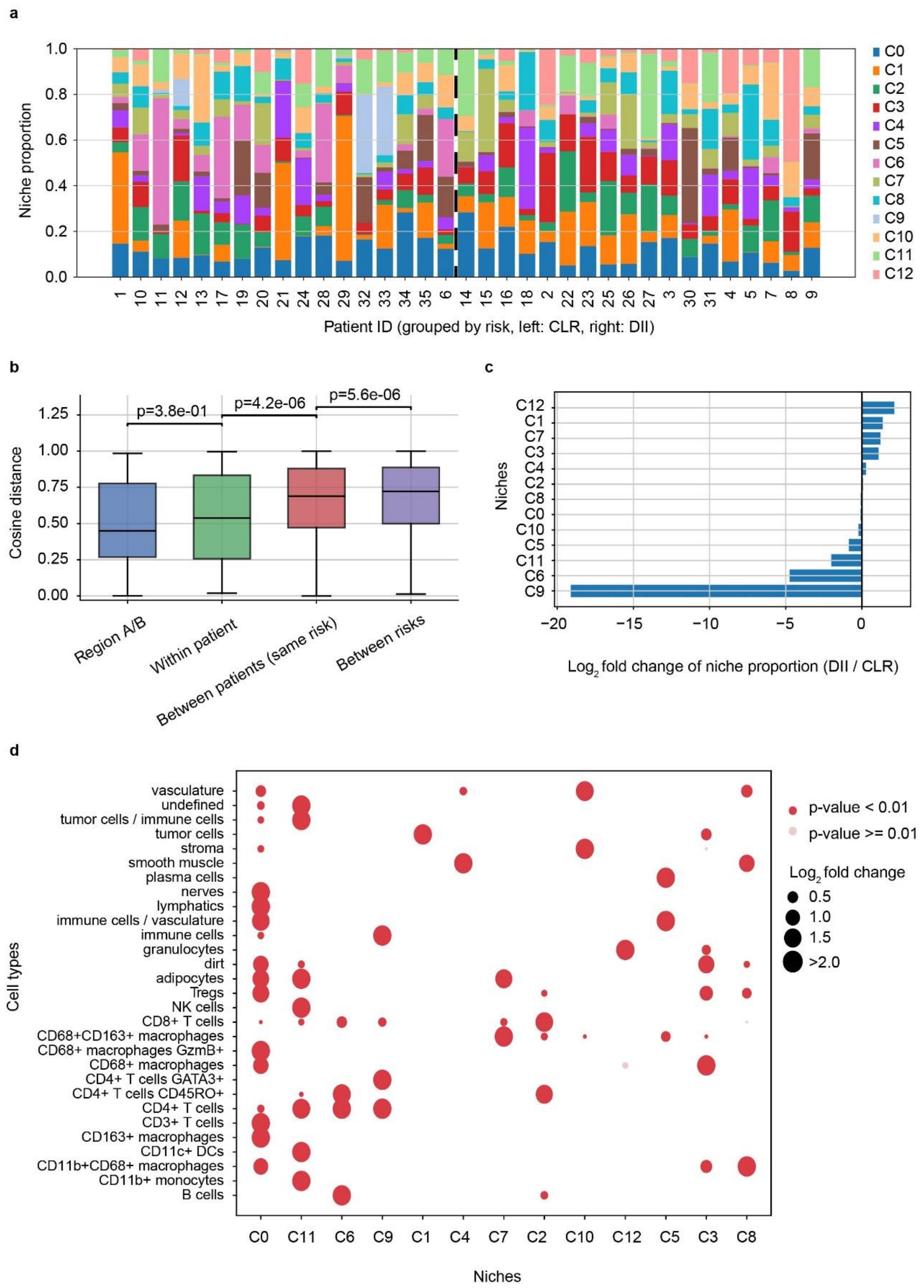

Supplementary Fig. 14: Characterization of CellNiche-defined microenvironmental niches in the human CRC cohort.

a, *Patient-level niche compositions visualized as stacked bar plots (C0–C12). Patients are grouped by clinical risk (left: CLR; right: DII), with the dashed line demarcating the two risk*

groups. ***b,*** *Cosine distances between patient/region niche-composition profiles derived from CellNiche in the CRC cohort. Each profile is a vector of niche proportions (C0–C12) for a given region or patient, and cosine distances were computed across four groups: (i) region A/B pairs from the same region ID, (ii) different regions within the same patient, (iii) different patients with the same CLR/DII risk classification, and (iv) patients with different risk classifications. Boxplots summarize the distribution of pairwise cosine distances for each comparison group.* ***c,*** *Risk-associated niche shifts summarized by the* $\log_2$ *fold change of patient-level niche proportion (DII/CLR) for each niche (C0–C12), highlighting niches preferentially represented in DII or CLR patients.* ***d,*** *Cell type enrichment in each niche (n = 13 niches).*

2. Please carefully check the order and citations of all supplementary figures in the main text.

Response:

We thank the reviewer for this helpful reminder. We have conducted a comprehensive audit of the Supplementary Information and the main text. Based on the logical flow of the results, we have reorganized the supplementary figures to ensure they strictly align with their mentions and citations in the main manuscript. This restructuring provides a more intuitive and streamlined experience for the reader.

3. Missing titles for supplementary figures 12 and 15.

Response:

We appreciate the reviewers' feedback. We have added to the revised version to ensure that each figure has good guidance.

Point-by-point response

Reviewer #1 (Remarks to the Author):

All my comments are addressed, and I have no further comments.

Response:

We thank the reviewer for their careful evaluation of our work and for the constructive feedback provided during the review process. We are pleased that all comments have been satisfactorily addressed in the revised manuscript.

Reviewer #2 (Remarks to the Author):

I thank the authors for the detailed response, and am happy to recommend the paper for publication.

Response:

We sincerely thank the reviewer for their thoughtful assessment and for recommending our manuscript for publication. We appreciate the reviewer’s time and helpful input throughout the review process.

Reviewer #3 (Remarks to the Author):

The authors have addressed most of the questions. A few minor comments for further improvement are noted below:

The ordering of Supplementary Figure in the main text is incorrect: subfigure 11a is positioned before subfigure 10, and subfigure 14b precedes subfigure 14a.

Response:

Thank you for pointing out this issue and for the time and assistance provided during the review process. We have corrected the ordering of Supplementary Figure citations in the main text.
